# Microbial diversity and adaptation Strategies in the nunataks of Western Dronning Maud Land, Antarctica

**DOI:** 10.3389/fmicb.2026.1921961

**Published:** 2026-10-22

**Authors:** Purnima Singh, Shiv Mohan Singh

**Affiliations:** 1 Indian Institute of Technology, Banaras Hindu University (IIT-BHU), Varanasi, India; 2 Department of Botany, Banaras Hindu University, Varanasi, India

**Keywords:** 16sRNA, Antarctica, bacterial diversity, adaptation, biogeography, FAME, MALDI-TOF MS

## Abstract

Antarctic soil ecosystems harbor unique microbial communities that survive under prolonged low temperatures, high UV radiation, desiccation, oligotrophic conditions and other environmental stresses. This study aimed to characterize the culturable microbial communities and explore potential adaptation strategies of microorganisms inhabiting the unique inland nunataks of the Jutulsessen and Ahlmannryggen ranges in Western Dronning Maud Land (WDML), Antarctica. Bacterial and fungal isolates representing colony morphotypes were isolated from soil samples using different growth media. Bacterial isolates were identified and characterized using matrix-assisted laser desorption ionization time-of-flight mass spectrometry (MALDI-TOF MS) with the MALDI BioTyper, 16S rRNA gene sequencing, and fatty acid methyl ester (FAME) analysis. Of the 96 bacterial isolates analyzed by MALDI-TOF MS, 75 produced good-quality spectral profiles, including 13 of isolates identified down to the species level, while the remaining 62 high-quality spectral profiles, represent an important contribution to the currently limited MALDI-TOF MS reference database of Antarctic bacteria. 16S rRNA sequencing identified 37 species of bacteria belonging to several ecologically diverse genera, namely *Acinetobacter, Arthrobacter, Bacillus, Brevundimonas, Brachybacterium, Curtobacterium, Dermacoccus, Dietzia, Janibacter, Knoellia, Kocuria, Kytococcus, Lysinibacillus, Microbacterium, Micrococcus, Methylobacterium, Moraxella, Paracoccus, Pontibacter, Pseudomonas, Pseudarthrobacter, Rhodococcus, Rothia*, *Sphingomonas, Stenotrophomonas,* and *Staphylococcus*. This bacterial assemblage is not only taxonomically diverse but also contains taxa with established ecological links to cold and extreme environments. Internal transcribed spacer (ITS) domain sequencing indicates the presence of four fungal genera, namely *Gelatinofungus, Leuconeurospora, Pseudogymnoascus,* and *Tolypocladium,* with ecological affinity for polar environments. FAME analysis revealed a predominance of branched-chain and unsaturated fatty acids, which help maintain cellular membrane fluidity as an adaptation strategy for the harsh Antarctic environment. In conclusion, the results demonstrate that the nunatak soils of Western Dronning Maud Land harbor a diverse culturable microbial assemblage closely related to cold and extreme environments, together with fatty-acid characteristics consistent with physiological adaptation to Antarctic conditions. To the best of our knowledge, this study provides the first comprehensive account of the culturable microbial communities inhabiting the nunataks of the Jutulsessen and Ahlmannryggen ranges in Western Dronning Maud Land, Antarctica.

## 1 Introduction

The biogeographical distribution of organisms across space and through time reflects the complex interplay between environmental conditions and evolutionary history (Terauds et al., 2012). Environmental, stochastic processes, and evolutionary drift also control the biogeography of microorganisms (Green et al., 2008; Niederberger et al., 2008; Terauds et al., 2012; Chong et al. 2015). The pattern of microbial diversity can be characterized both on fine spatial and on continental scales, both of which are influenced by landscape, climate, and geomorphology (Ferrari et al., 2015; Staebe et al., 2019; van Goethem et al., 2016; van Dorst et al., 2017; Zumsteg et al., 2013). Spatial distribution patterns of microorganisms have been suggested to be more evident in polar environments, and are still a focus area of investigation (Niederberger et al., 2008; Staebe et al., 2019).

Although polar regions encompass vast areas, only approximately 2% of the terrestrial surface is ice-free during the summer. These ice-free regions constitute highly discontinuous and spatially heterogeneous habitats, encompassing diverse soil types, varying degrees of cryoturbation, and complex topographic features (Cary et al., 2010; Fernández-Martínez et al., 2021; Ferrari et al., 2015; Ji et al., 2015). Antarctic soil habitats are exposed to harsh climatic and oligotrophic conditions (Chown et al., 2015), resulting in the evolution of microbes with unique survival mechanisms for dealing with extremely low temperatures, desiccation, strong winds, hypersalinity, and variable UV radiation flux (Dong et al., 2015). In particular, Antarctica’s soils are oligotrophic and face rapid and wide-ranging fluctuations in temperature. As a result of these physico-chemical pressures, Antarctic soils typically exhibit extremely low microbial biodiversity (Cowan et al., 2015; Niederberger et al., 2008; O’Neill et al., 2013). Furthermore, substantial differences exist between coastal ice-free ornithogenic soils and the oligotrophic soils of inland ice-free nunataks (Hofstee et al., 2006; Michel et al., 2006; Brinkmann et al., 2007; Pires et al., 2017; Staebe et al., 2019). Edaphic factors are predominantly responsible for the spatial heterogeneity of microbes in continental Antarctica (Smith et al., 2010; Lee et al., 2012; Bottos et al., 2020; Severgnini et al., 2021) as well as in maritime Antarctica (Chong et al., 2010). Furthermore, some microbial taxa appear to be endemic to Antarctica, whereas others exhibit broad, potentially cosmopolitan distributions across geographically distant environments (Vyverman et al., 2010; Jung et al., 2019; Gutt et al., 2021; Velichko et al., 2021; Cong et al., 2020). Climate change is increasingly altering ecological equilibria and microbial community dynamics (Gutt et al., 2021). Changes in environmental conditions may affect microbial colonization, succession, dispersal, and evolutionary processes, thereby reshaping microbial community composition and ecosystem functioning (van Dorst et al., 2017). Understanding microbial diversity and adaptation in isolated Antarctic habitats is therefore particularly important for assessing their responses to ongoing environmental change.

Current knowledge of the temporal and spatial distribution of microorganisms in Antarctic soils is limited (Fernández-Martínez et al., 2021; Yergeau and Kowalchuk, 2008; Yergeau et al., 2012; Varliero et al., 2024). The majority of microbial studies conducted in Antarctica have focused on the Antarctic Peninsula and accessible regions of continental Antarctica using metagenomic approaches. Previous studies on culture-independent approaches of Antarctic soils have frequently identified Actinobacteria, Acidobacteria, Bacteroidetes, Proteobacteria, and Firmicutes among the major bacterial groups, although their relative abundance varies between coastal, maritime, and inland continental environments (Aislabie et al., 2006; Cary et al., 2010; Chown et al., 2015; Ji et al., 2015; Savaglia et al., 2024; Varliero et al., 2024). Teixeira et al. (2010) described the phylum Firmicutes as dominant, along with *Bifidobacterium*, *Arcobacter,* and *Faecalibacterium* from the rhizospheric soil of Admiralty Bay. Freckman and Virginia (1997) reported Proteobacteria as dominant bacteria from the ice-free area of the Keller Peninsula. In addition to cyanobacteria, ribulose-1,5-bisphosphate carboxylase/oxygenase (Rubisco) has been reported across diverse bacterial phyla, including Proteobacteria (Alpha-, Beta-, and Gammaproteobacteria), Firmicutes, Chloroflexi, Actinobacteria, and Verrucomicrobia (Berg, 2011; Khadem et al., 2011; Grostern and Alvarez-Cohen, 2013; Tahon et al., 2016). The detection of genes associated with carbon fixation and phototrophic processes in diverse bacterial lineages further suggests that Antarctic bacteria can occupy functionally important niches despite the severe environmental limitations of these habitats. Huang et al. (2013) reported 12 bacterial phyla and 110 genera using NGS from Schirmacher Oasis. Targeted amplification of the *cbbL* and *pufLM* genes in inland terrestrial Antarctic soils has revealed the presence of microorganisms harboring genetic markers associated with carbon fixation and phototrophic metabolism (Tahon et al., 2016; Tahon and Willems, 2017; Staebe et al., 2019). Phototrophic and potentially autotrophic bacteria were detected in inland Antarctic soils, indicating that microbial primary production may contribute to nutrient cycling in nutrient-poor terrestrial ecosystems. Recently, Varliero et al. (2024) investigated the continental-scale biogeography of Antarctic soil bacterial communities using 16S rRNA gene amplicon datasets and the Antarctic Conservation Biogeographic Regions (ACBR) framework. Their study provided valuable insights into the spatial structuring of bacterial communities across geographically distinct Antarctic regions. The contrasting patterns indicate that Antarctic bacterial communities are structured by strong environmental filtering and that different terrestrial habitats may select for bacteria with distinct physiological and ecological characteristics. Beyond taxonomic diversity, Antarctic bacteria are ecologically important because many possess physiological and metabolic traits that facilitate survival under low temperature, desiccation, nutrient limitation, high salinity, strong winds, and fluctuating ultraviolet radiation.

Though culture-independent techniques were widely used, culturable approaches (culturomics) are rare despite being important for understanding the phenotypic characteristics of these microbes (Pham and Kim, 2012; Vartoukian et al., 2010). Focusing on culturable microorganisms is particularly advantageous because their isolation and characterization enable direct assessment of their physiological properties and biotechnological potential. Antarctic microorganisms, in particular, may represent a valuable source of novel natural products and biomolecules with potential applications in the food, industrial, and pharmaceutical sectors (Stafsnes et al., 2013). The culturable bacterial genera reported across Antarctic environments encompassed five major phyla, with Pseudomonadota (Proteobacteria) representing the largest group (29 genera), followed by Actinomycetota (17), Bacteroidota (7), Bacillota (4), and Deinococcota (1). The predominance of Pseudomonadota and Actinomycetota reflects the broad ecological distribution and physiological versatility of these bacterial groups in Antarctic terrestrial, freshwater, marine, and glacial environments (Peeters et al., 2011; Bajerski and Wagner, 2013; Kirby et al., 2012; Zdanowski et al., 2013; González-Rocha et al., 2017; Rizzo et al., 2020). The recurring occurrence of the genera *Arthrobacter, Pseudomonas, Psychrobacter, Sphingomonas, Hymenobacter, Cryobacterium, Polaromonas, Janthinobacterium, Flavobacterium, Chryseobacterium, Pedobacter, Brevundimonas* and *Rhodococcus* across geographically distinct Antarctic habitats demonstrates considerable taxonomic diversity and suggests their broad ecological competence and ability to persist under low temperature, limited nutrient availability, desiccation, and other environmental stresses. The culturable fungal community reported from Antarctic soils comprised predominantly members of the phylum Ascomycota, including *Geomyces*, *Pseudeurotium*, *Rhizoscyphus*, *Lecythophora* and *Aspergillus*, with additional representatives of Basidiomycota (*Rhodotorula*) and Mucoromycota (*Mortierella*), highlighting the taxonomic breadth of fungi capable of persisting in Antarctic terrestrial environments (Cong et al., 2020). The previous studies indicate that Antarctic culturable microbiota are not represented by a single characteristic community, but comprise habitat-specific assemblages enriched in taxa with diverse physiological capabilities for survival under cold, saline, oligotrophic, and other extreme conditions (Bajerski and Wagner 2013; Zdanowski et al., 2013; Zhao et al., 2016; González-Rocha et al., 2017; Rizzo et al., 2020; Cong et al., 2020). Much of the available microbial diversity data have been generated using culture-independent approaches from relatively well-studied regions such as the Antarctic Peninsula and accessible regions of continental Antarctica. In contrast, the culturable microbial communities inhabiting geographically isolated inland nunataks of continental Antarctica remain comparatively poorly characterized or unexplored. The limited information available on the spatial distribution and adaptive characteristics of microorganisms in these environments highlights the need for further investigation of their culturable bacterial communities.

Matrix-assisted laser desorption ionization time-of-flight mass spectrometry (MALDI-TOF MS) has emerged as an important tool for microbial protein fingerprinting and rapid taxonomic identification, with considerable potential for applications in microbial ecological and diversity studies. The principle of microbial identification by MALDI-TOF MS is based on the characteristic protein composition of individual microorganisms, which generates reproducible mass spectral fingerprints. MALDI-TOF MS profiling of abundant cellular proteins, particularly ribosomal proteins and other low-molecular-weight proteins, enables comparison of these fingerprints with reference spectra and thereby facilitates rapid microbial identification and assessment of taxonomic affiliation. MALDI-TOF MS, coupled with dedicated software and reference databases containing protein profiles derived from whole bacterial cells, provides a rapid, cost-effective, and reliable approach for microbial identification (Seng et al., 2009; Alispahic et al., 2010; Emonet et al., 2010). Consequently, this technique has been increasingly adopted in microbial culturomics for the rapid screening and identification of large numbers of cultured microbial isolates (Lagier et al., 2016).

Although MALDI-TOF MS is a powerful and widely used tool for rapid microbial identification, its application to protein profiling of microorganisms from polar regions remains comparatively limited. Pantucek et al. (2018) and Sedláček et al. (2020) characterized novel *Hymenobacter* and *Staphylococcus* species isolated from James Ross Island, Antarctica, using a polyphasic approach that included molecular methods, MALDI-TOF MS, and lipid and cellular fatty acid analyses. More recently, He et al. (2022) employed MALDI-TOF MS to identify 33 yeast species from Arctic and Antarctic environments. Although 16S rRNA gene sequencing remains one of the most widely used approaches for bacterial identification, MALDI Biotyper (MB), supported by dedicated reference databases, has increasingly been applied for high-throughput microbial identification and complementary phenotypic characterization (Davies et al., 2012; Pham et al., 2024). Nevertheless, the identification of microorganisms from extreme environments remains challenging because polar and other environmental taxa are underrepresented in existing MALDI-TOF MS reference databases, which can limit the accuracy and reliability of identification (Kopcakova et al., 2014; Timperio et al., 2017).

Hassan et al. (2020) reported temperature-dependent alterations in the fatty acid profiles of cold-adapted bacteria, highlighting the role of membrane lipid composition in adaptation to low temperatures. Similarly, Prakash et al. (2014) demonstrated that environmental stresses can influence cellular fatty acid composition in members of the genus *Micrococcus*. Previous studies from polar environments have also highlighted the importance of fatty acid composition in maintaining membrane fluidity, flexibility, and permeability under extreme conditions (Akulava et al., 2024; Singh et al., 2024). In general, increased proportions of saturated and cyclopropane fatty acids enhance membrane rigidity and reduce permeability, whereas higher proportions of cis-unsaturated fatty acids increase membrane fluidity and permeability. In contrast, an increased proportion of anteiso-fatty acids generally promotes membrane fluidity, particularly at low temperatures (Mező et al., 2022). These changes in membrane fatty acid composition represent an important adaptive mechanism that enables microorganisms to maintain membrane functionality under cold and other environmental stresses.

The primary objective of the present study was to investigate the culturable bacterial and fungal communities inhabiting soil samples collected from geographically isolated nunataks of Western Dronning Maud Land (WDML), Antarctica. Microbial isolates were investigated using MALDI Biotyper-based MALDI-TOF MS protein profiling, 16S rRNA gene and ITS sequence analysis for taxonomic identification, while fatty acid profiling was performed to assess potential physiological adaptations to the isolated and extreme conditions prevailing in Antarctic nunatak habitats. This work contributes to the very few published data on culturable microbial diversity of Antarctica. Characterization of culturable microorganisms from relatively unexplored WDML Antarctic habitats remains important for identifying ecologically adapted bacterial and fungal taxa and for understanding the functional attributes that enable microbial survival in extreme polar environments.

## 2 Methods and materials

### 2.1 Sampling and site description

Soil samples were collected from ice-free areas of nine geographically isolated nunataks in Western Dronning Maud Land (WDML), Antarctica: Flårjuven (72°01′S, 3°22′W), Valterkulten (71°54′S, 3°14′W), Slettfjel (72°08′S, 3°19′W), Schumacherfjellet (71°55′S, 2°58′W), Grunehogna (72°03′S, 2°43′W), Robertskollen (71°29′S, 3°13′W), Lorentzenpiggen (71°46′S, 2°49′W), Vesleskarvet (71°40′22″S, 2°50′26″W), and Vassdalen in the Jutulsessen area (72°00′39″S, 2°32′03″E) (Figure 1). In addition, soil samples were collected from a penguin rookery located near the continental margin (70°31′26.7″S, 8°04′70.6″W). Soil replicates were collected from the different nunataks and adjacent areas for subsequent microbial and soil physicochemical analyses. Prior to sampling, the uppermost 1–2 cm of soil was carefully removed to minimize the influence of surface contaminants, and subsurface soil was collected from the underlying layer. All sampling procedures were performed aseptically to minimize potential contamination. Sterile spatulas, disposable gloves, alcohol, sterile Falcon tubes, and clean plastic bags were used during sample collection and handling. Each replicate comprised multiple soil subsamples collected aseptically within a 1-m radius and then samples were immediately stored at −20 °C until microbiological analyses were performed. Soil organic carbon, total available nitrogen, total available phosphorus, and metal concentrations were determined as part of a previous study and have been reported together with metagenomic data (Staebe et al., 2019). These previously published physicochemical data were used for comparison and ecological interpretation of the microbial diversity observed in the present culture-based study.

**Figure 1:**
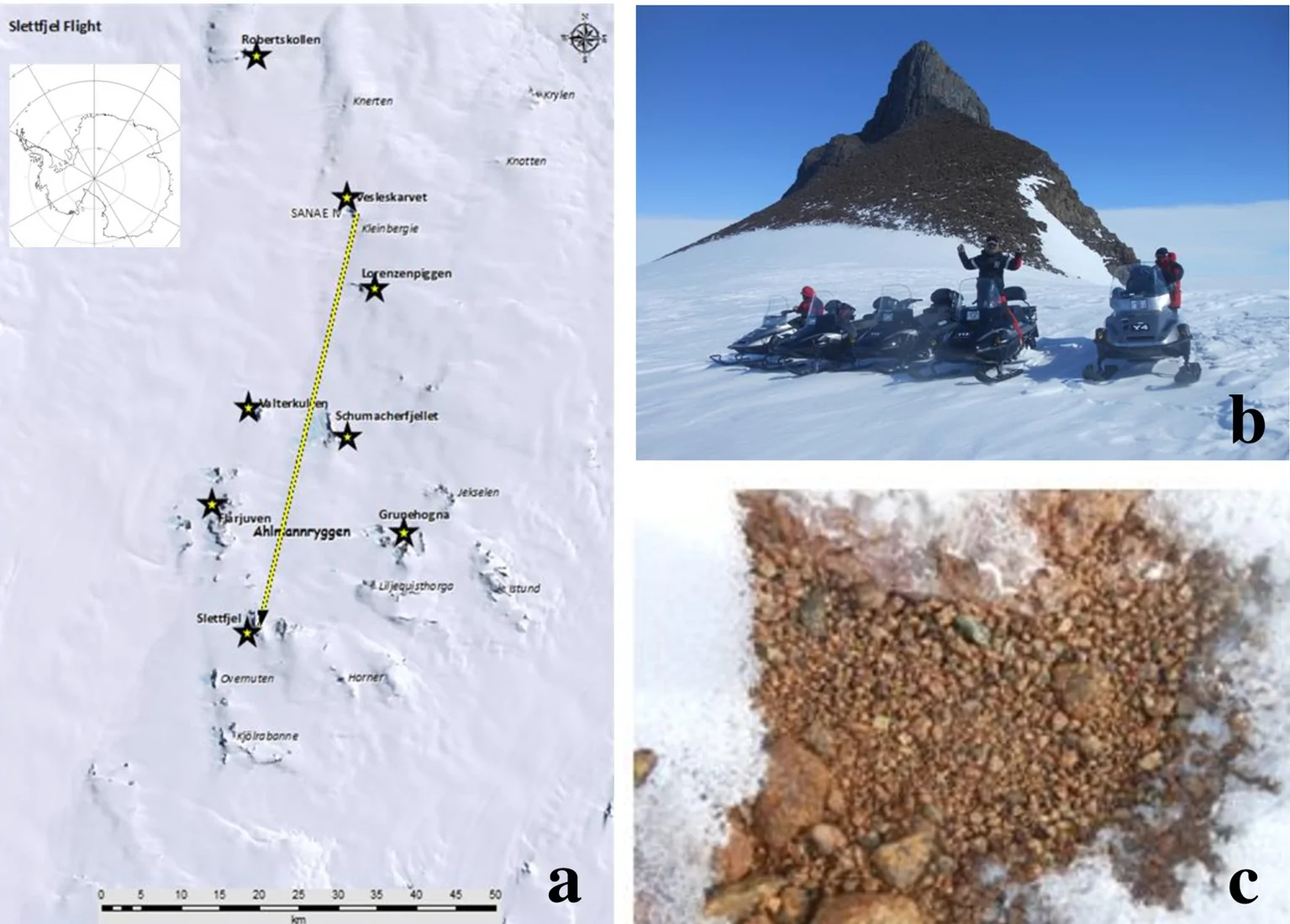
**(a)** Map showing sampling locations in WDML, Antarctica **(b)** Landscape of a nunatak highlighting huge ice area with a rocky, snow-dusted hill under a clear blue sky, **(c)** Exposed soil with reddish-brown gravel and rocks after melting of snow and ice.

### 2.2 Isolation and enumeration of culturable microorganisms

For microbial isolation, 1 g of soil was suspended in 9 mL of saline solution and serially diluted (10^−1^, 10^−2^ and 10^−3^). In order to maximize the recovery of culturable microorganisms, and account for differences in nutritional requirements, a range of bacteriological and fungal media, including media of reduced nutrient strength, was used. Culturable bacteria were enumerated using the spread-plate method by inoculating 0.1 mL of each appropriate dilution onto Nutrient Agar (NA), 1/10-strength NA, Marine Broth (MB), 1/10-strength MB, Antarctic Bacterial Medium (ABM), 1/10-strength ABM, and R2A agar, as described by Singh et al. (2014). For fungal isolation, 100 μL of the appropriate soil suspension was inoculated onto Rose Bengal Agar (RBA), Potato Dextrose Agar (PDA), and Malt Extract Agar (MEA) using spread- and pour-plate techniques. Inoculated plates were incubated at 1, 4, and 15 °C for 2–4 weeks to facilitate the recovery of psychrophilic and psychrotolerant microorganisms. Colony morphology, pigmentation and representative colony characteristics were examined using a stereo-zoom microscope (Nikon SMZ1500). Bacterial isolates were examined by Gram staining to determine their cellular and staining characteristics. To assess the physiological tolerance and growth characteristics of the bacterial isolates, growth experiments were performed on ABM plates under different temperatures (1, 4, 15, 22, and 30 °C), pH conditions (3, 5, 7, 9, and 11), and NaCl concentrations (0, 2, 5, 7, and 15%) following Singh et al. (2014). Isolates were selected from individual plates on the basis of distinct colony morphotypes were purified. The pure isolates obtained were stored in glycerol stock at −70 °C, and unique cultures were submitted to National Centre for Microbial Resource (NCMR; MCC), Pune, India.

### 2.3 DNA extraction, polymerase chain reaction (PCR), and sequencing

Genomic DNA from bacterial isolates was extracted using the standard phenol–chloroform extraction method (Sambrook et al., 1989), followed by DNA purification and PCR amplification of the 16S rRNA gene using the universal primers 16F27 (5′-CCAGAGTTTGATCMTGGCTCAG-3′) and 16R1492 (5′-TACGGYTA CCTTGTT ACGA CTT-3′). Fungal genomic DNA was similarly extracted and purified, followed by PCR amplification of the internal transcribed spacer (ITS) region using the universal fungal primers ITS1 (5′-TCCGTAGGTGAACCTGCGG-3′) and ITS4 (5′-TCCTCCGCTTATTGATATGC-3′). The amplified 16S rRNA gene and ITS regions were purified by PEG–NaCl precipitation and directly sequenced in both directions using an ABI 3730XL automated DNA sequencer (Applied Biosystems, Foster City, CA, United States). The resulting sequences were assembled using the Lasergene software package (Boratyn et al., 2013). Bacterial sequences were subjected to similarity searches using NCBI BLASTn (Altschul et al., 1997) and the EzTaxon-e database (Kim et al., 2012), whereas fungal sequences were analyzed using BLASTn and the EzFungi database (Kim et al., 2012). Taxonomic assignments were based on sequence similarity, query coverage, and the quality of the corresponding reference sequences. High-quality nucleotide sequences from 81 bacterial isolates were deposited in GenBank under accession numbers MF467808–MF467852, MF541526–MF541542, MF952724–MF952732, and MK202346–MK202355. Similarly, sequences from 10 fungal isolates were deposited in GenBank under accession numbers MF467853–MF467862. For phylogenetic analysis, the 16S rRNA gene sequences of the bacterial isolates were aligned using the ClustalW. The evolutionary history was inferred using the Neighbor-Joining method (Saitou and Nei, 1987). The tree is drawn to scale, with branch lengths in the same units as those of the evolutionary distances used to infer the phylogenetic tree. The evolutionary distances were computed using the Maximum Composite Likelihood method (Tamura et al., 2004) and Evolutionary analyses were conducted in MEGA7 (Kumar et al., 2016). The resulting phylogenetic trees were used to assess the evolutionary relationships between the Antarctic bacterial isolates and their closely related reference taxa.

### 2.4 MALDI-TOF MS analysis

#### 2.4.1 Sample preparation, MALDI-TOF MS measurement, and MALDI BioTyper identification

A total of 96 bacterial cultures (pure isolates) were selected for MALDI-TOF MS analysis. Fresh cultures were grown on Nutrient Agar (NA), Potato Dextrose Agar (PDA), Marine Broth (MB), or Antarctic Bacterial Medium (ABM), depending on the medium from which the respective isolate was originally recovered. Sample preparation for MALDI-TOF MS analysis was performed using both the direct-smear and protein-extraction methods following the standard protocol described by Alatoom et al. (2011). For the direct-smear method, a loopful of fresh, actively growing bacterial culture was applied as a thin film onto a MALDI target plate. The bacterial biomass was overlaid with 1 μL of MALDI matrix solution consisting of a saturated solution of α-cyano-4-hydroxycinnamic acid (HCCA) prepared in 50% acetonitrile and 2.5% trifluoroacetic acid (TFA), and the preparation was allowed to dry at room temperature before analysis. When reproducible spectra could not be obtained using the direct-smear method, a protein-extraction procedure was employed. Briefly, a loopful of actively growing bacterial biomass was suspended in molecular-grade water and vortexed thoroughly. The suspension was mixed with 70% (v/v) ethanol and centrifuged at 10,000 rpm for 2 min. The supernatant was carefully discarded, and the resulting pellet was air-dried at room temperature. The dried pellet was subsequently resuspended in 70% (v/v) formic acid and acetonitrile, mixed thoroughly, and centrifuged at 10,000 rpm for 2 min to remove cellular debris. One microlitre of the resulting supernatant was spotted onto the MALDI target plate and allowed to dry at room temperature, followed by the addition of 1 μL of HCCA matrix solution. The MALDI-TOF MS instrument was calibrated using a bacterial test standard (Bruker Daltonics), and instrument performance was validated prior to sample analysis. Bacterial samples were analyzed using an Autoflex Speed MALDI-TOF MS system (Bruker Daltonik GmbH, Germany). Mass spectra were acquired over a mass-to-charge ratio (m/z) range of 2,000–20,000 Da using a laser frequency of 1,000 Hz in linear positive-ion mode. The instrument parameters were as follows: ion source 1 voltage, 19.5 kV; ion source 2 voltage, 18.2 kV; lens voltage, 7 kV; and extraction delay time, 240 ns. The acquired bacterial mass spectra were processed and visualized using MALDI Biotyper software version 3.0 (Bruker Daltonik). Spectral quality was assessed on the basis of the intensity, reproducibility, and number of peaks obtained. Spectra showing sufficient quality and reproducibility were classified as good-quality spectra, whereas isolates that repeatedly failed to produce satisfactory spectra after repeated measurements were classified as bad/poor-quality spectra. For taxonomic identification, good-quality spectra were compared with reference Main Spectra Profiles (MSPs) contained in the Bruker MALDI Biotyper reference database using MALDI Biotyper Compass/MBT Compass software. The software calculates a similarity-based score between the experimental spectrum and the reference MSPs, with the resulting score used to assess the taxonomic affiliation of each isolate. Identification was performed according to the score thresholds recommended for the corresponding taxonomic level. For comparative analysis of the MALDI-TOF MS profiles, processed spectra were subjected to principal component analysis (PCA) to visualize overall similarities and differences among the bacterial isolates. PCA was used as an exploratory multivariate approach to determine whether isolates exhibiting similar protein spectral fingerprints formed recognizable clusters. Hierarchical clustering was additionally performed using the MALDI Biotyper software, and dendrograms were generated using the unweighted pair-group method with arithmetic mean (UPGMA) based on spectral similarity. Clusters containing isolates with closely related protein profiles were interpreted as groups exhibiting greater spectral similarity, whereas isolates located on distant branches were considered comparatively dissimilar in their MALDI-TOF MS protein fingerprints. The PCA and dendrogram analyses were used primarily to visualize patterns of similarity among the isolates and were considered complementary to sequence-based taxonomic identification. Figure 2 was generated using Bruker MALDI Biotyper software and incorporates the relevant spectral preprocessing, PCA, identification-score thresholds, and hierarchical clustering procedures.

**Figure 2:**
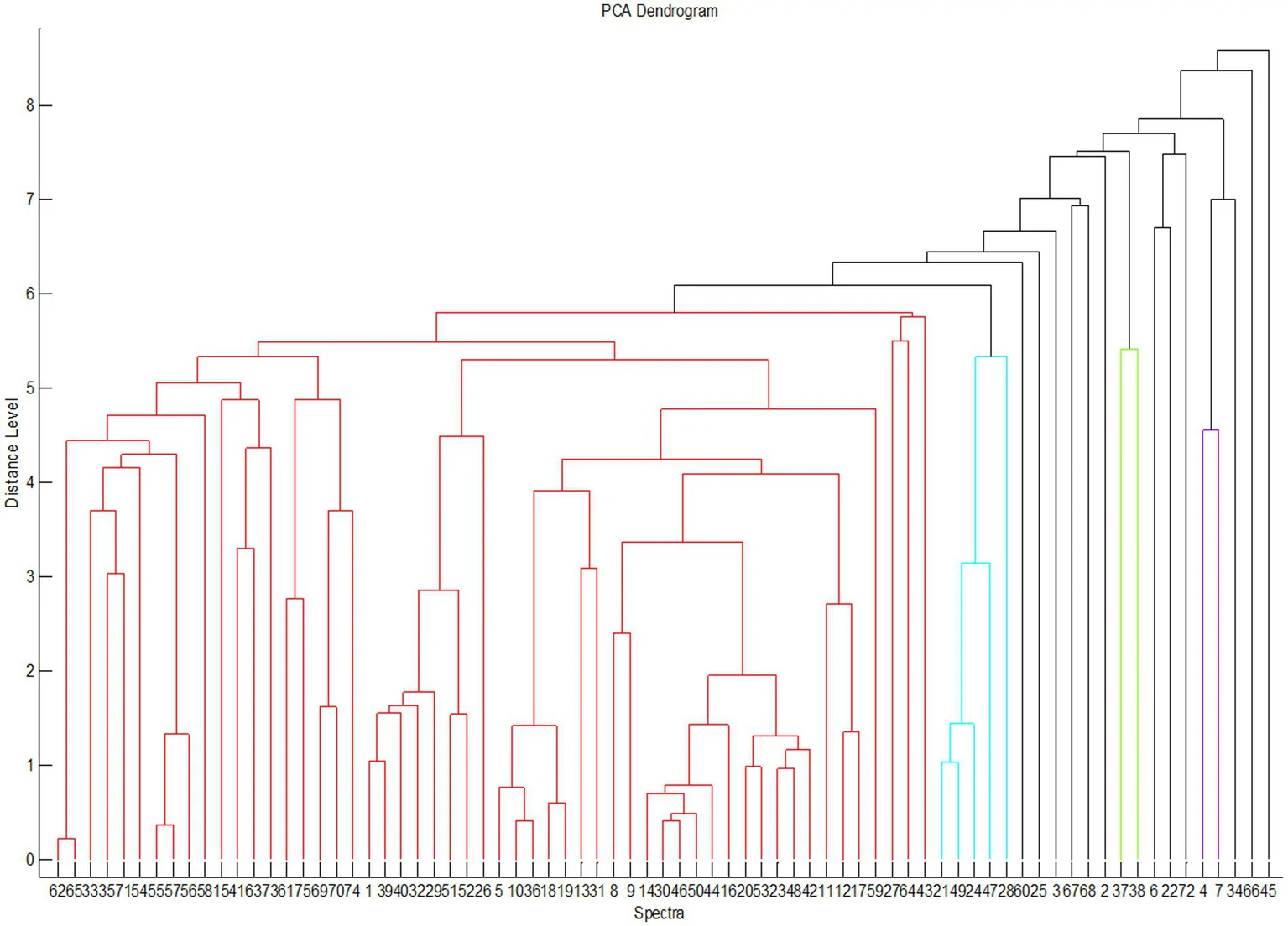
Dendrogram of MALDI TOF MS spectral profiling of bacterial strains.

### 2.5 Fatty acid methyl ester (FAME) analysis

For cellular fatty acid analysis, bacterial isolates were grown on Tryptic Soy Broth Agar (TSBA; HiMedia) and, where applicable, on the respective isolation media under the same temperature conditions used for their initial recovery. Cultures were incubated at 4 and 15 °C for 2–3 weeks. To obtain biological replicates, each bacterial isolate was streaked onto three independent plates containing the same medium and incubated under similar conditions. Extraction and analysis of cellular fatty acid methyl esters (FAMEs) were performed according to the method described by Buyer (2008). FAMEs were analyzed using an Agilent 7890A gas chromatograph (Agilent Technologies, CA, United States) equipped with a flame ionization detector (FID), an autosampler, and an HP-5 capillary column (25 m × 0.20 mm × 0.33 μm). Hydrogen was used as the carrier gas. Prior to sample analysis, the gas chromatographic system was calibrated twice using calibration standard 1300AA (MIDI, USA), and calibration was automatically verified after every 11th sample. The extraction efficiency and authenticity of the detected fatty acid profiles were verified using the type strain *Stenotrophomonas maltophilia* ATCC 13637ᵀ, which has a well-characterized fatty acid profile, as a positive control. Individual FAMEs were identified by comparing their retention times with those of reference standards analyzed under identical chromatographic conditions. Peak identification and fatty acid profiling were performed using the Sherlock Microbial Identification System (MIS) software and the RTSBA6 reference database (MIDI Inc., Newark, DE, United States).

## 3 Results

### 3.1 Physicochemical characteristics of soils and cultures

The elemental and physicochemical characteristics indicate that the nunataks of Western Dronning Maud Land (WDML) represent heterogeneous and generally nutrient-limited terrestrial environments. Differences in altitude, exposure history, substrate characteristics, pH, organic carbon, and nutrient availability are likely to influence microbial establishment, persistence, and community development (Staebe et al., 2019). Across the nine investigated nunataks, nitrogen concentrations ranged from approximately 17.0 to 50.0 ppm, phosphorus from 14.3 to 45.6 ppm, and organic carbon from 0.89 to 1.84%. Valterkulten had the highest organic carbon concentration (1.84%), whereas Flårjuven, Grunehogna, and Schumacherfjellet showed comparatively lower concentrations of approximately 1.12–1.30%; Slettfjell exhibited an intermediate value of 1.55%. Nitrogen concentrations were comparatively high at Grunehogna (49.98 ppm) and Valterkulten (44.78 ppm), whereas Lorentzenpiggen and Slettfjell had lower concentrations of 22.19 and 22.43 ppm, respectively. Overall, the relatively low organic-carbon concentrations and limited nutrient pools are consistent with the nutrient-limited conditions commonly characteristic of Antarctic terrestrial ecosystems.

Soil pH values generally ranged from approximately 6.0 to 7.0, indicating predominantly mildly acidic to near-neutral conditions. Lorentzenpiggen had the lowest measured pH (6.13), followed by Grunehogna (6.20), Flårjuven (6.31), Valterkulten (6.68), and Slettfjell (6.85) (Staebe et al., 2019). These pH conditions are not intrinsically restrictive for many microorganisms and may therefore permit the establishment and persistence of diverse bacterial communities, despite the more pronounced constraints imposed by low temperature, limited liquid-water availability, desiccation, and seasonal environmental fluctuations. Consequently, physicochemical conditions at these sites may provide a relatively permissive chemical environment within an otherwise climatically extreme ecosystem.

The elemental composition further demonstrates pronounced geochemical heterogeneity among the WDML nunataks, with individual sites exhibiting distinct elemental signatures. Such variation may reflect differences in parent material and mineralogy, degree of weathering, sediment development, atmospheric deposition, and local microenvironmental processes. The marked enrichments of Cr, Ni, and Co at Lorentzenpiggen; As at Robertskollen; Cu and Zn at Valterkulten and Robertskollen; and Cd, Tl, Pb, Cs, U, Li, and Fe at Jutulsessen (Vassdalen) indicate strong site-specific geochemical influences. These differences may create localized gradients in micronutrient availability, thereby contributing to the environmental heterogeneity experienced by microbial communities. Such geochemical variation, together with differences in substrate development and climatic exposure, may influence microbial colonization, selection, and community assembly across the isolated nunataks of WDML.

Of the numerous colony-forming units (CFUs) that developed on the culture media plates, 200 morphologically distinct representative isolates were selected and purified for further characterization (Figure 3). The recovered colonies exhibited considerable phenotypic diversity, with predominant pigmentation ranging from white and cream to yellow and orange. Colonies generally displayed entire margins, opaque and smooth surfaces, and convex, pulvinate, or raised elevations. Microscopic examination following Gram staining revealed both coccoid and rod-shaped bacterial cells, ranging from short to long rods. Most isolates exhibited growth over a temperature range of 1–22 °C, with an optimum temperature of approximately 15 °C. These growth characteristics indicate that the majority of the isolates were cold-adapted. The isolates also demonstrated tolerance to NaCl concentrations ranging from 1 to 7%, indicating their ability to withstand moderate osmotic stress. Growth experiments conducted over a range of pH values showed that pH 6.0–7.0 was generally optimal, indicating a preference for mildly acidic to near-neutral conditions. Collectively, these physiological characteristics suggest that the culturable bacterial community possesses traits that may facilitate persistence under the low-temperature, variable moisture, and chemically heterogeneous conditions prevailing in Antarctic nunatak soils.

**Figure 3:**
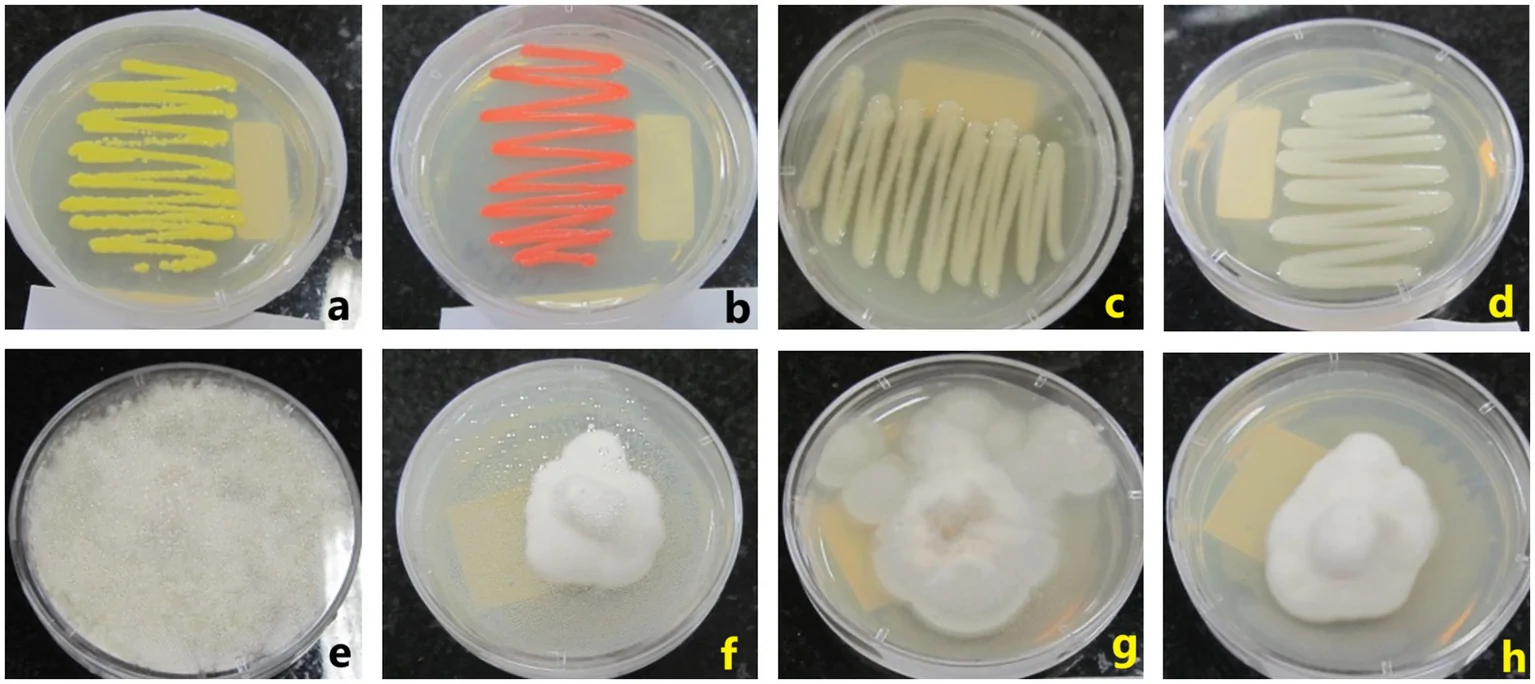
Bacterial and fungal colony morphologies isolated from Western Dronning Maud Land (WDML), Antarctica. Representative bacterial **(a–d)** and fungal **(e–h)** colonies showing distinct morphological characteristics on agar media. Colonies exhibited variation in colour, texture, and growth form, including yellow, orange, smooth white, and fluffy white morphologies.

### 3.2 Bacterial identification by MALDI-TOF MS spectral profiling

Of the 96 bacterial cultures analyzed by MALDI-TOF MS, reproducible, good-quality mass spectra were obtained for 75 isolates, whereas 21 isolates produced poor-quality spectra. A representative good-quality MALDI-TOF MS spectrum is shown in Figure 4. Among the 96 isolates analyzed, 65% produced good-quality spectra but could not be assigned a reliable taxonomic identity using the available reference database, 9% were identified only to the genus level, 4% were identified to the species level, and 22% produced poor-quality spectra (Figure 5). The good-quality spectra were compared with reference main spectral profiles (MSPs) in the MALDI Biotyper 3.0 database (Bruker Daltonik, Germany). Reliable database matches were obtained for 13 isolates, representing seven genera (*Arthrobacter, Brevundimonas, Dermacoccus, Kocuria, Micrococcus, Sphingomonas,* and *Staphylococcus*) and 11 species (Table 1). The remaining 62 isolates generated good-quality spectral profiles but did not obtain reliable matches with the available reference spectra (Supplementary Table 1 and Supplementary Figure 1). Thus, the results reflect the limited representation of Antarctic and other environmental microorganisms in the MALDI Biotyper reference database, highlighting the need for expansion of reference spectral libraries for polar microorganisms.

**Figure 4:**
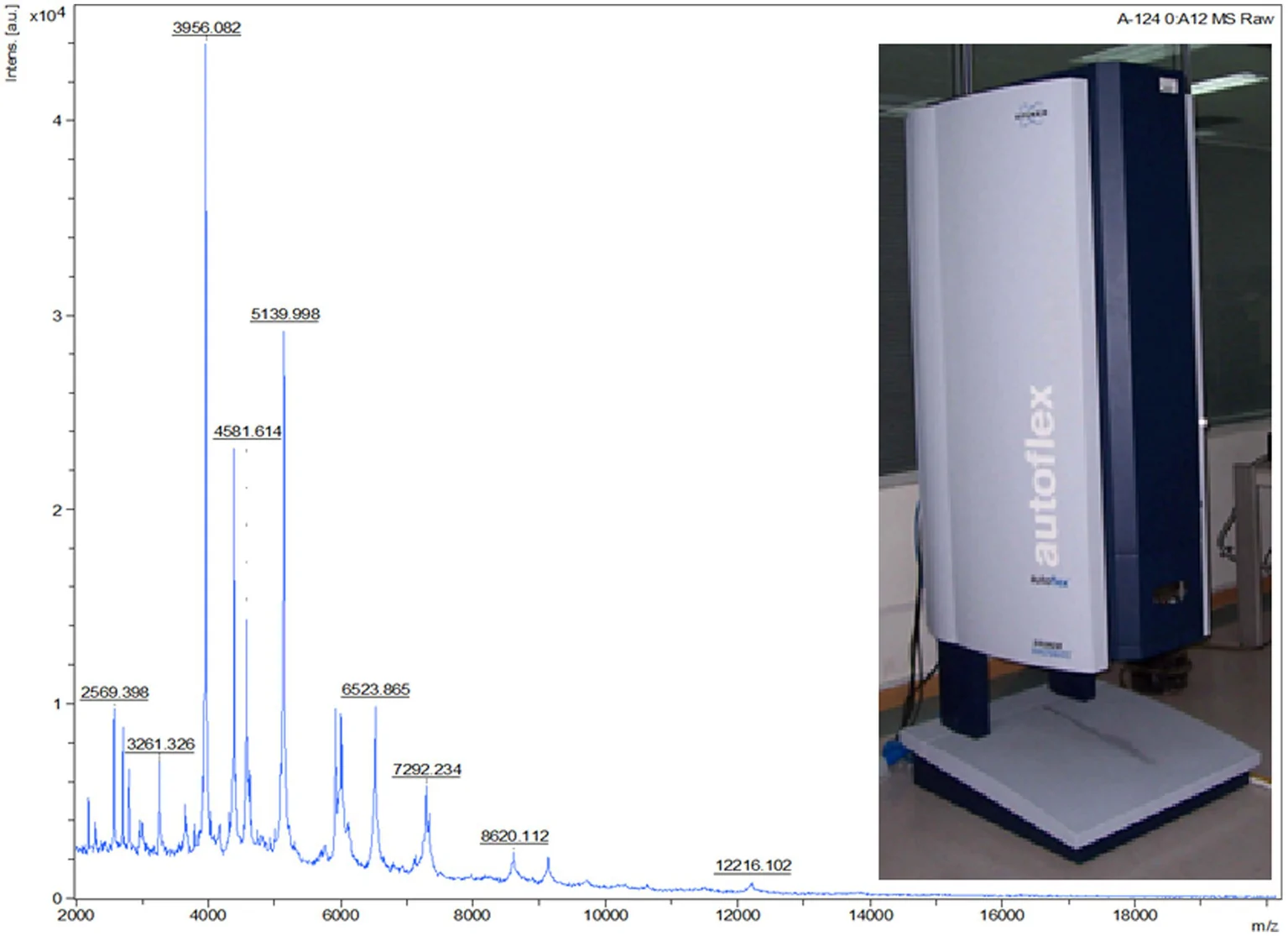
A representative MALDI-TOF mass spectrum showing the characteristic protein fingerprint of a bacterial isolate, with signal intensity plotted against mass-to-charge ratio (m/z). Multiple characteristic peaks are observed across the approximately 2,500–12,200 m/z range. The Bruker Autoflex MALDI-TOF mass spectrometer used for microbial identification is shown on the right.

**Figure 5:**
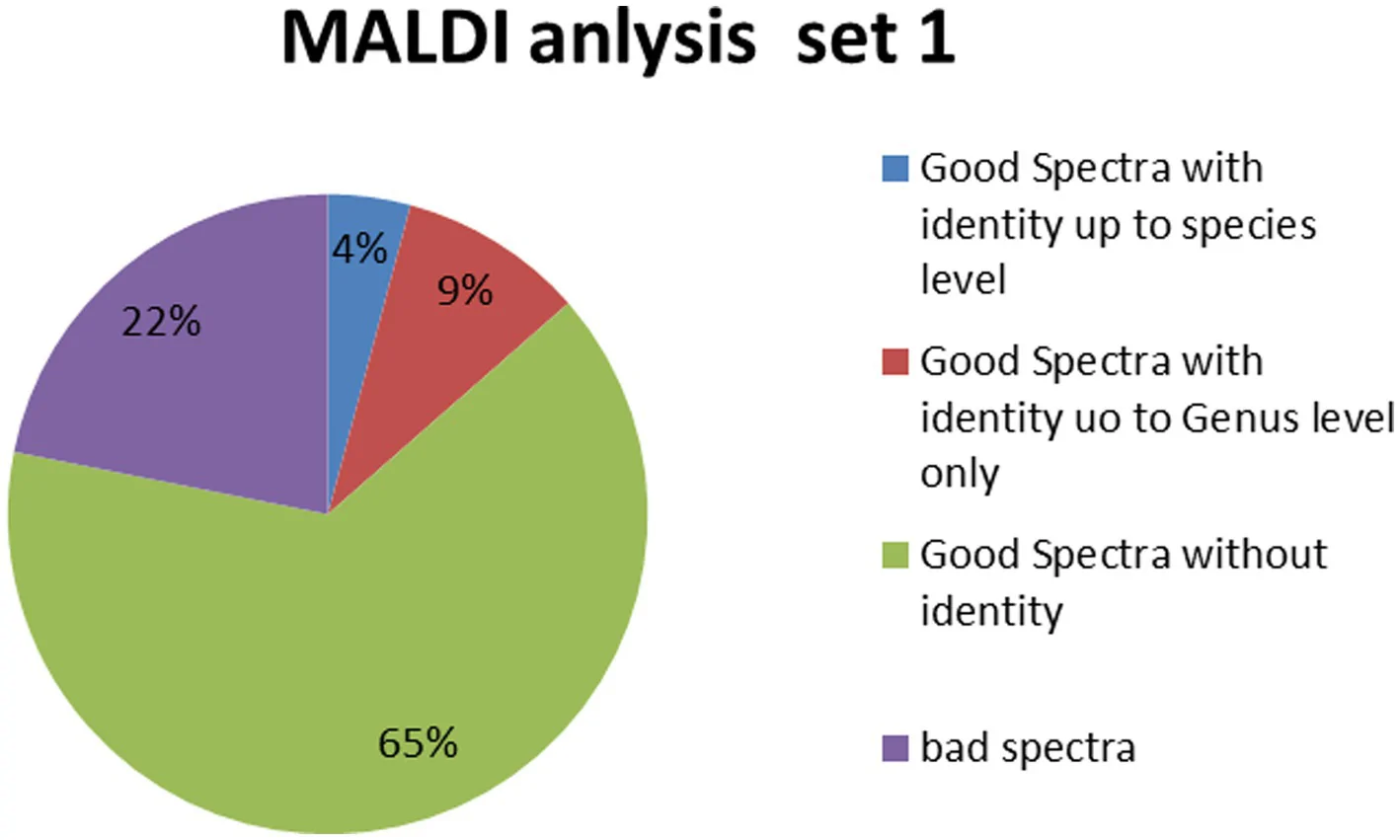
The pie chart shows the distribution of bacterial isolates according to the quality and taxonomic resolution of their MALDI-TOF MS spectra. Good-quality spectra were identified to the species level (4%), to the genus level only (9%); good spectra without identity (65%); and 22% of isolates generated poor-quality/bad spectra.

**Table 1:** Identification of bacterial isolates by MALDI TOF-MS analysis.

S. no.	Bacterial isolate	Bacterial identity matching with MALDI database	Score
1	A-46	*Micrococcus luteus*	2.105
2	PENG-3	*Staphylococcus arlettae*	2.022
3	A-155	*Arthrobacter flavus*	2.022
4	A-33	*Kocuria palustris*	2.004
5	A-08	*Sphingomonas paucimobilis*	1.988
6	A-71	*Brevundimonas vesicularis*	1.871
7	PM-35	*Arthrobacter citreus*	1.827
8	A-10	*Dermacoccus nishinomiyaensis*	1.799
9	A-144	*Staphylococcus hominis*	1.738
10	A-45	*Micrococcus luteus*	1.72
11	A-62	*Arthrobacter sulfonivorans*	1.713
12	A-134	*Arthrobacter oxydans*	1.711
13	A-147	*Arthrobacter oxydans*	1.701

MALDI-TOF MS spectral profiles were further subjected to hierarchical clustering and visualized as a dendrogram (Figure 2). The profiles of the seven (A-8, A-62, A-71, A-10, A-33, A-46, A-144) identified genera were distributed among distinct clusters, while several isolates belonging to the same genus showed relatively close clustering, indicating similarity in their protein fingerprints. For example, four of the five *Arthrobacter* isolates identified in the study clustered within the same major clade at a distance level below 4, suggesting relatively similar protein profiles with some species-level variation. In contrast, isolate PM-35, identified as *Arthrobacter citreus*, clustered separately from the other *Arthrobacter* isolates. This difference may reflect strain-level variation in protein expression or genomic background.

MALDI-TOF MS successfully generated informative protein fingerprints for a substantial proportion of the bacterial isolates and provided database-supported identification for 13 isolates representing seven genera and 11 species (Table 1). The repeated detection of genera such as *Arthrobacter, Sphingomonas, Brevundimonas, Micrococcus,* and *Dermacoccus* is consistent with the occurrence of bacterial taxa previously associated with cold and polar environments and suggests that the culturable assemblage includes microorganisms capable of persistence under Antarctic environmental conditions. The occurrence of *Micrococcus luteus, Kocuria palustris, Dermacoccus nishinomiyaensis,* and *Brevundimonas vesicularis* is compatible with their reported occurrence in cold habitats. In contrast, the recovery of *Staphylococcus* spp. from remote Antarctic soils may potentially result from environmental dispersal, transient colonization, wildlife-associated inputs, or anthropogenic contamination.

Collectively, the MALDI-TOF MS results demonstrate the utility of protein fingerprinting as a rapid screening and complementary identification approach for culturable Antarctic bacteria. The large proportion of good-quality spectra that lacked reliable database matches also emphasizes the current limitations of reference spectral libraries for environmental and polar microorganisms. These unidentified profiles represent valuable resources for future comparative studies and for expanding MALDI-TOF MS reference databases for Antarctic bacterial diversity.

### 3.3 Bacterial identification using 16S rRNA

16S rRNA gene-based identification of the bacterial isolates revealed considerable taxonomic diversity within the sampled nunatak environments, with 37 species representing 27 genera and 19 families (Table 2). The identified taxa belonged to diverse bacterial families, including Bacillaceae, Caulobacteraceae, Dietziaceae, Dermabacteraceae, Dermacoccaceae, Hymenobacteraceae, Kytococcaceae, Intrasporangiaceae, Methylobacteriaceae, Microbacteriaceae, Micrococcaceae, Moraxellaceae, Nocardioidaceae, Nocardiaceae, Pseudomonadaceae, Rhodobacteraceae, Sphingomonadaceae, Staphylococcaceae, and Xanthomonadaceae. The broad phylogenetic representation indicates that Antarctic nunatak soils can support diverse culturable bacterial assemblages despite of extreme environmental conditions of these habitats.

**Table 2:** Culturable bacteria isolated from nunataks and other soils in Jutulsessen and Ahlmannryggen ranges of Western Dronning Maud Land, Antarctica.

Sr. no.	Strain no.	Sampling locality	Color of the colony	Medium	Growth temperature	Accession No.	Closest homolog	Percent similarity
1	A-116	Valterkulten	Cream	R2A	15 °C	MF467830	*Arthrobacter flavus* strain CMS 19Y, (NR_104688.1)	100.0
2	A-117	Valterkulten	Yellow	R2A	15 °C	MF467831	*Arthrobacter flavus* strain CMS 19Y, (NR_104688.1)	99.8
3	A-118	Valterkulten	Yellow	R2A	15 °C	MF467832	*Arthrobacter flavus* strain CMS 19Y, (NR_104688.1)	99.6
4	A-119	Valterkulten	Cream	R2A	15 °C	MF467833	*Arthrobacter flavus* strain CMS 19Y, (NR_104688.1)	99.6
5	A-120	Valterkulten	Cream	R2A	15 °C	MF541537	*Arthrobacter flavus* JCM 11496(T), (AB537168)	99.52
6	A-121	Valterkulten	Yellow	R2A	15 °C	MF467834	*Arthrobacter flavus* strain CMS 19Y, (NR_104688.1)	99.5
7	A-122	Valterkulten	Light Yellow	R2A	15 °C	MF467835	*Arthrobacter flavus* strain CMS 19Y, (NR_104688.1)	99.9
8	A-71	Valterkulten	Orange	1/10PDA	15 °C	MF541532	*Brevundimonas vesicularis* NBRC 12165(T), (BCWM01000033)	100.00
9	A-3	Valterkulten	White	PDA	15 °C	MF467808	*Dermacoccus nishinomiyaensis* strain DSM 20448 (NR_044872.1)	99.4
10	A-6	Valterkulten	Red	RBA	15 °C	MF467809	*Stenotrophomonas pavanii* strain LMG 25348 (NR_118008.1)	99.4
11	A-35	Valterkulten	Cream	YEPDA	15 °C	MF467815	*Brachybacterium rhamnosum* strain H-6S (NR_042109.1)	99.9
12	A-36	Valterkulten	Orangish	YMA	15 °C	MF467816	*Dermacoccus nishinomiyaensis* strain DSM 20448, (NR_044872.1)	99.2
13	A-37	Valterkulten	White	YMA	15 °C	MF952725	*Kocuria palustris* strain TAGA27, (NR_026451.1)	99.8
14	A-131	Valterkulten	Yellow	R2A	15 °C	MF467839	*Arthrobacter flavus* strain CMS 19Y, (NR_104688.1)	99.5
15	A-132	Valterkulten	Greenish	R2A	15 °C	MF467840	*Arthrobacter flavus* strain CMS 19Y, (NR_104688.1)	99.8
16	A-133	Valterkulten	Yellowish	R2A	15 °C	MF467841	*Arthrobacter flavus* strain CMS 19Y, (NR_104688.1)	99.9
17	A-39	Valterkulten	Yellowish	YPMA	15 °C	MF467817	*Arthrobacter flavus* strain CMS 19Y, (NR_104688.1)	100.0
18	A-8	Drill Hole -2 m	Yellow	YPMA	15 °C	MF467810	*Sphingomonas sanguinis* strain NBRC 13937 (NR_113637.1)	99.4
19	A-9	Drill Hole -2 m	Cream	MGYPA	15 °C	MF467811	*Moraxella osloensis* strain NCTC 10465, (KT989843.1)	99.5
20	A-10	Drill Hole -2 m	Yellowish	MGYPA	15 °C	MF541526	*Dermacoccus nishinomiyaensis* DSM 20448(T), (X87757)	99.72
21	A-11	Drill Hole -2 m	Yellow	YEPDA	15 °C	MF467812	*Dermacoccus nishinomiyaensis* strain DSM 20448, (NR_044872.1)	98.8
22	A-28	Flarjuven	Cream	YPMA	15 °C	MF952724	*Dermacoccus nishinomiyaensis* DSM 20448, (NR_044872.1)	99.8
23	A-29	Flarjuven	Orange	YPMA	15 °C	MF467813	*Staphylococcus arlettae* strain ATCC 43957, (NR_024664.1)	99.9
24	A-33	Flarjuven	Cream	MGYPA	15 °C	MF467814	*Kocuria palustris* strain TAGA27 (NR_026451.1)	100.0
25	A-45	Slettjell	Yellow	ABM	15 °C	MF952726	*Micrococcus yunnanensis* YIM 65004 (CP001628.1)	99.1
26	A-47	Slettjell	Orange	YMA	15 °C	MF952727	*Dermacoccus nishinomiyaensis* DSM 20448, (NR_044872.1)	99.6
27	A-49	Slettjell	Orange	ABM	15 °C	MF541527	*Dermacoccus nishinomiyaensis* DSM 20448(T), (X87757)	99.72
28	A-101	Slettjell	Orange	ABM	15 °C	MF541535	*Staphylococcus gallinarum* ATCC 35539(T), D83366	100.00
29	A-50	Schumacherfjellet	White	ABM	1°C	MF467818	*Arthrobacter oxydans* strain DSM 20119, (NR_026236.1)	99.2
30	A-51	Schumacherfjellet	Cream	ABM	1°C	MF467819	*Arthrobacter ginsengisoli* strain DCY81, (KF212463.1)	99.9
31	A-54	Schumacherfjellet	Cream	ABM	15 °C	MF467820	*Arthrobacter oxydans* strain DSM 20119, (NR_026236.1)	98.7
32	A-58	Schumacherfjellet	White	ABM	15 °C	MF467821	*Rothia* sp. 140917_MRSA_09, (KU878871.1)	97.6
33	A-59	Schumacherfjellet	White	YPMA	15 °C	MF541528	*Pseudarthrobacter siccitolerans* 4J27(T), (CAQI01000001)	99.31
34	A-60	Schumacherfjellet	Cream	YEPDA	15 °C	MF467822	*Pseudomonas hunanensis* strain LV (JX545210.1)	99.7
35	A-62	Schumacherfjellet	White	YMA	15 °C	MF467823	*Arthrobacter ginsengisoli* strain DCY81, (KF212463.1)	99.3
36	A-63	Schumacherfjellet	Greenish	ABM	15 °C	MF541529	*Microbacterium phyllosphaerae* DSM 13468(T), AJ277840	99.86
37	A-64	Schumacherfjellet	White	ABM	15 °C	MF467824	*Arthrobacter polychromogenes* strain DSM 20136, (NR_026192.1)	99.0
38	A-65	Schumacherfjellet	Greenish	YEPDA	15 °C	MF541530	*Microbacterium phyllosphaerae* DSM 13468(T), AJ277840	98.55
39	A-66	Schumacherfjellet	White	YEPDA	15 °C	MF952728	*Pseudarthrobacter phenanthrenivorans* Sphe3T, (NR_074770.1)	98.8
40	A-67	Schumacherfjellet	Orange	YEPDA	15 °C	MF467825	*Dermacoccus nishinomiyaensis* strain DSM 20448, (NR_044872.1)	99.5
41	A-68	Schumacherfjellet	Orange	MGYPA	15 °C	MF541531	*Dermacoccus nishinomiyaensis* DSM 20448(T), X87757	99.84
42	A-69	Schumacherfjellet	White	YMA	15 °C	MF467826	*Arthrobacter ginsengisoli* strain DCY81, (KF212463.1)	99.4
43	A-106	Schumacherfjellet	White	1/10PDA	15 °C	MF467829	*Arthrobacter ginsengisoli* strain DCY81, (KF212463.1)	99.8
44	A-107	Schumacherfjellet	White	1/10PDA	15 °C	MF541536	*Acinetobacter baumannii* ATCC 19606(T), (ACQB01000091)	99.93
45	A-134	Schumacherfjellet	Gray	YMA	4 °C	MF467842	*Arthrobacter oxydans* strain DSM 20119, (NR_026236.1)	99.3
46	A-135	Schumacherfjellet	White	YMA	4 °C	MF467843	*Microbacterium phyllosphaerae* strain P 369/06, (NR_025405.1)	99.7
47	A-136	Schumacherfjellet	White	YMA	4 °C	MF467844	*Arthrobacter oxydans* strain DSM 20119, (NR_026236.1)	99.3
48	A-137	Schumacherfjellet	Gray	ABM	4 °C	MF467845	*Arthrobacter oxydans* strain DSM 20119, (NR_026236.1)	98.9
49	A-138	Schumacherfjellet	Yellow	MGYPA	4 °C	MF467846	*Microbacterium phyllosphaerae* strain P 369/06, (NR_025405.1)	99.5
50	A-147	Schumacherfjellet	Cream	R2A	15 °C	MF467849	*Arthrobacter oxydans* strain DSM 20119, (NR_026236.1)	98.9
51	A-72	Grunehogna	White	1/10PDA	15 °C	MF541533	*Aeromicrobium ginsengisoli* Gsoil 098(T), AB245394	99.86
52	A-141	Grunehogna	Pink	R2A	15 °C	MF467847	*Arthrobacter agilis* strain DSM 20550, (NR_026198.1)	99.5
53	A-80	Lorentzenpiggen	White	1/10PDA	15 °C	MF467827	*Dietzia aerolata* strain Sj14a (NR_116932.1)	99.7
54	A-82	Lorentzenpiggen	Cream	PDA	15 °C	MF952729	*Pseudarthrobacter phenanthrenivorans* Sphe3, (NR_074770.1)	97.6
55	A-83	Lorentzenpiggen	Yellow	PDA	15 °C	MF541534	*Rhodococcus sovatensis* H004(T) KU189221	99.08
56	A-145	Lorentzenpiggen	Cream	R2A	15 °C	MF467848	*Knoellia subterranea* strain HKI 0120, (NR_028932.1)	97.9
57	A-146	Lorentzenpiggen	Yellow	R2A	15 °C	MF952730	*Dermacoccus nishinomiyaensis* DSM 20448, (NR_044872.1)	98.7
58	A-90	Robertskollen	Cream	ABM	15 °C	MF467828	*Arthrobacter sulfonivorans* strain ALL (NR_025084.1)	98.6
59	A-123	Robertskollen	White	R2A	15 °C	MF467836	*Arthrobacter ginsengisoli* strain DCY81, (KF212463.1)	99.2
60	A-124	Robertskollen	Cream	R2A	15 °C	MF467837	*Arthrobacter oryzae* strain KV-651, (NR_041545.1)	98.8
61	A-129	Robertskollen	Cream	R2A	15 °C	MF467838	*Arthrobacter sulfonivorans* strain ALL, (NR_025084.1)	98.6
62	A-153	Penguin Rookery	Yellow	R2A	15 °C	MF952731	*Dermacoccus nishinomiyaensis* DSM 20448(T), (X87757)	99.79
63	A-155	Penguin Rookery	Cream	R2A	15 °C	MF952732	*Paracoccus acridae* SCU-M53(T) (KT634253)	98.19
64	Peng-1	Penguin Rookery	Cream	1/10PDA	15 °C	MF541538	*Lysinibacillus sphaericus* KCTC 3346(T), AUOZ01000024	99.80
65	Peng-3	Penguin Rookery	orange	1/10ABM	15 °C	MF467850	*Staphylococcus arlettae* strain ATCC 43957, (NR_024664.1)	99.7
66	Peng-4	Penguin Rookery	Cream	1/10NA	15 °C	MF541539	*Lysinibacillus sphaericus* KCTC 3346(T), (AUOZ01000024)	99.80
67	Peng-6	Penguin Rookery	Cream	1/10NA	15 °C	MF541540	*Acinetobacter baumannii* ATCC 19606(T), ACQB01000091	100.00
68	Peng-7	Penguin Rookery	Yellow	NA	15 °C	MF541541	*Kytococcus aerolatus* 02-St-019/1(T) (FM992368)	99.03
69	Peng-8	Penguin Rookery	Cream	NA	15 °C	MF541542	*Lysinibacillus sphaericus* KCTC 3346(T), AUOZ01000024	99.80
70	Peng-9	Penguin Rookery	Red	NA	15 °C	MF467851	*Methylobacterium rhodesianum* strain DSM 5687, (NR_041028.1)	99.4
71	Peng-10	Penguin Rookery	Yellow	NA	15 °C	MF467852	*Curtobacterium luteum* strain DSM 20542, (NR_026157.1)	99.5
72	NB-1	Vesleskarvet, Northern Buttress	Pink	NA	15 °C	MK202346	*Rhodococcus aerolatus* PAMC 27367(T), (KM044053)	95.66
73	NB-3	Vesleskarvet, Northern Buttress	Yellow	NA	15 °C	MK202347	*Sphingomonas daechungensis* CH15-11(T), (JQ772481)	97.62
74	NB-8	Vesleskarvet, Northern Buttress	Yellow	NA	15 °C	MK202354	*Kocuria palustris* (Y16263)	100.00
75	NB-15	Vesleskarvet, Northern Buttress	Pink	NA	15 °C	MK202348	*Rhodococcus aerolatus* PAMC 27367(T), (KM044053)	95.37
76	VD-1	Vassdalen, Troll	White	NA	15 °C	MK202355	*Bacillus paramycoides* (KJ812444)	99.42
77	VD-3	Vassdalen, Troll	Cream	NA	15 °C	MK202349	*Bacillus altitudinis* 41KF2b(T), (ASJC01000029)	100.00
78	VD-5	Vassdalen, Troll	Yellowish cream	NA	15 °C	MK202351	*Janibacter indicus* 0704P10-1(T), (HM222655)	99.72
79	VD-7	Vassdalen, Troll	White	NA	15 °C	MK202350	*Bacillus cereus* ATCC 14579(T), (AE016877)	100.00
80	VD-15	Vassdalen, Troll	Orange	NA	15 °C	MK202352	*Pontibacter amylolyticus* 9-2(T) (KF660586)	94.66
81	VD-30	Vassdalen, Troll	Orange	NA	15 °C	MK202353	*Dermacoccus nishinomiyaensis* DSM 20448(T), (X87757)	99.69

YPMA, yeast extract, peptone, mannitol; MGYPA, malt extract, yeast extract, peptone, glucose; YEPDA, yeast extract, peptone, dextrose; YMA, peptone, yeast extract, malt extract, dextrose; PDA, potato dextrose Agar; RBA, Rose Bengal Agar. ABM, (Antarctic bacterial medium)-peptone, yeast extract, agar.

Most isolates exhibited high sequence similarity to their closest reference sequences, generally ranging from 98 to 100%, providing strong support for their taxonomic affiliation. In contrast, several isolates showed comparatively lower similarities, including *Rhodococcus aerolatus* (95.37–95.66%), *Pontibacter amylolyticus* (94.66%), *Rothia* sp. (97.6%), and *Pseudarthrobacter phenanthrenivorans* (97.6%). These relatively low sequence similarities may indicate substantial phylogenetic divergence from the currently available reference sequences and warrant further investigation using polyphasic taxonomic approaches. Micrococcaceae was the most frequently represented family in the present study, with *Arthrobacter* being particularly prominent. A total of 27 *Arthrobacter* strains representing seven species, while *Dermacoccus* represented the second most frequently recovered genus, with 12 isolates. The repeated recovery of multiple strains belonging to the same species suggests that these taxa were not merely isolated sporadically but may represent relatively persistent components of the culturable bacterial community. Several Proteobacteria, including *Pseudomonas hunanensis, Sphingomonas sanguinis, Paracoccus acridae, Acinetobacter baumannii, Moraxella osloensis,* and *Stenotrophomonas pavanii*, were also recovered. The coexistence of Actinomycetota and Proteobacteria suggests that the nunatak habitats can accommodate bacteria with different ecological and physiological characteristics. Proteobacterial taxa may exploit spatially heterogeneous nutrient resources and transient organic substrates, whereas many Actinomycetota possess physiological characteristics that can favor persistence under nutrient limitation and desiccation.

The recovery of *Bacillus*, *Lysinibacillus* and other spore-forming or stress-resistant bacteria is also ecologically significant. The study identified *Bacillus altitudinis, B. cereus, B. paramycoides* and *Lysinibacillus sphaericus*. Such taxa may survive unfavorable periods through resistant cellular states and therefore potentially contribute to the long-term persistence of microbial populations in exposed Antarctic soils. The presence of taxa such as *Brevundimonas, Methylobacterium, Sphingomonas, Pseudomonas* and *Paracoccus* demonstrates that the microbial community also contains metabolically versatile bacteria. Their occurrence could reflect exploitation of trace organic compounds and other limited nutrient resources available in Antarctic soils and rock-associated microhabitats. The distribution of bacterial taxa also varied among the sampling locations. Valterkulten showed particularly frequent recovery of *Arthrobacter flavus*, with sequence similarities ranging from 99.5 to 100%. In addition, this site supported representatives of several other bacterial lineages, including *Brevundimonas vesicularis, Dermacoccus nishinomiyaensis, Stenotrophomonas pavanii, Brachybacterium rhamnosum,* and *Kocuria palustris*. The coexistence of these taxa indicates that Valterkulten provides suitable microsites for a phylogenetically diverse culturable bacterial assemblage. Schumacherfjellet exhibited comparatively high culturable diversity, with representatives of *Arthrobacter, Pseudarthrobacter, Pseudomonas, Microbacterium, Dermacoccus, Acinetobacter,* and *Rothia*. The repeated recovery of *A. oxydans, A. ginsengisoli,* and *Microbacterium phyllosphaerae* suggests that these taxa can persist across a range of heterogeneous microhabitats within this exposed terrestrial environment. Detecting multiple strains of the same species suggests that strain-level diversity may be important for survival in Antarctic habitats. The repeated detection of *Dermacoccus nishinomiyaensis* across geographically separated sampling locations was particularly noteworthy. This species was recovered from Valterkulten, drill-hole soils, Flårjuven, Slettfjell, Schumacherfjellet, Lorentzenpiggen, Jutulsessen (Vassdalen) and the Penguin Rookery. Its occurrence across multiple habitats suggests that *D. nishinomiyaensis* is relatively widespread within the sampled WDML environments and may possess physiological characteristics that permit persistence across geographically and environmentally heterogeneous sites.

Several less frequently recovered taxa further increased the taxonomic breadth of the culturable bacterial assemblage, including *Dietzia aerolata, Rhodococcus sovatensis, Knoellia subterranea, Paracoccus acridae, Kytococcus aerolatus, Methylobacterium rhodesianum, Curtobacterium luteum,* and *Janibacter indicus*. Their detection demonstrates that exposed-rock and soil habitats of WDML harbor representatives of diverse bacterial lineages. In Vassdalen, Bacillota included *Bacillus paramycoides, B. altitudinis,* and *B. cereus*, together with *Janibacter indicus, Pontibacter amylolyticus,* and *Dermacoccus nishinomiyaensis*. The occurrence of these phylogenetically diverse taxa indicates that the Vassdalen habitat supports a mixed culturable bacterial assemblage rather than being exclusively dominated by Actinomycetota. In contrast, the Vesleskarvet Northern Buttress soils yielded a comparatively restricted set of cultured taxa, including *Rhodococcus aerolatus, Sphingomonas daechungensis,* and *Kocuria palustris*. Such differences among sampling sites may reflect variation in substrate characteristics, nutrient availability, moisture conditions, exposure history, and other local environmental factors.

The phylogenetic relationships among the bacterial isolates were further examined using four phylogenetic trees (Figures 6A–D). These analyses provide an evolutionary framework for assessing the taxonomic relationships of the recovered isolates and complement the species-level assignments obtained from 16S rRNA gene sequence similarity searches. Collectively, the 16S rRNA gene results demonstrate that the culturable bacterial fraction of WDML nunatak soils encompasses substantial phylogenetic diversity, with repeated recovery of particular taxa suggesting their potential persistence across the extreme and heterogeneous Antarctic terrestrial environment.

**Figure 6:**
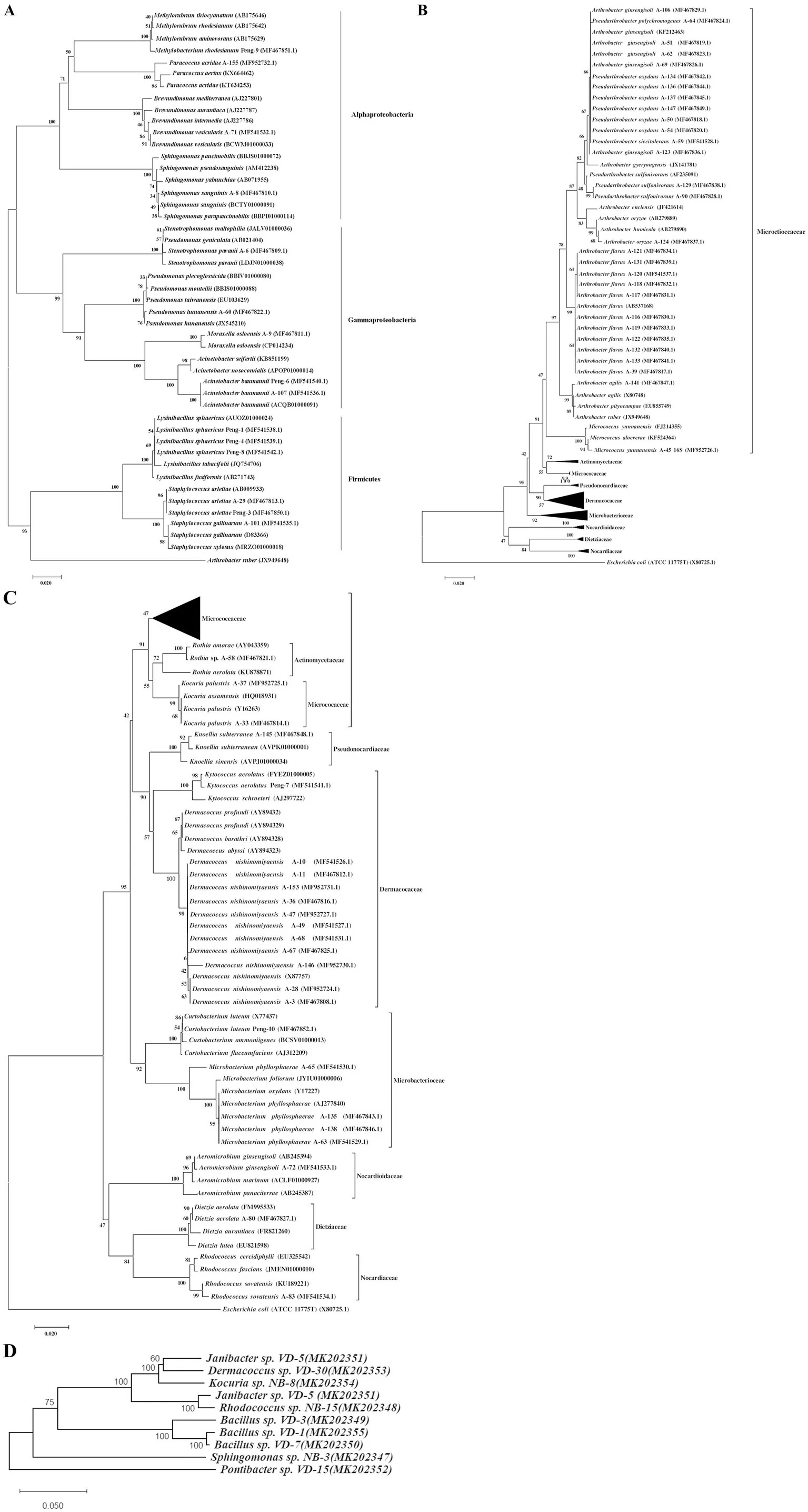
Phylogenetic relationships of bacterial isolates based on 16S rRNA gene sequences. **(A)** Phylogenetic tree showing the relationships among representative bacterial isolates belonging to the classes Alphaproteobacteria and Gammaproteobacteria and the phylum Firmicutes. **(B)** Detailed phylogenetic tree showing members of the family Micrococcaceae within the order Micrococcales, including isolates affiliated with the genera *Arthrobacter* and *Pseudarthrobacter*. Bacterial names and GenBank accession numbers are indicated alongside the branches. Bootstrap values shown at the nodes indicate the level of support for the corresponding branches. Phylogenetic tree **(C)** shows relationships among various bacterial taxa, with family-level clades such as Micrococcaceae and Dermacoccaceae labeled and bootstrap values indicated at nodes. Phylogenetic tree **(D)** presents bacteria isolated from the Vassdalen (Troll station) area, including *Janibacter, Dermacoccus, Bacillus*, and others, along with bootstrap support values. All trees include evolutionary distance scales.

### 3.4 Fungal identification using ITS region sequencing

Analysis of the internal transcribed spacer (ITS) region sequences from 10 fungal isolates revealed five taxonomically distinct fungal taxa with ITS similarities ranging from 95.84 to 100% (Table 3). Sequence-based identification indicated the presence of *Gelatinofungus brunneus* (MF467862), *Leuconeurospora bharatiensis* (MF467858), *Pseudogymnoascus roseus* (MF467855), *Tolypocladium ovalisporum* (MF467857), and *Samsoniella alpina* (MF467853). The identified isolates originated predominantly from Robertskollen, with *G. brunneus* recovered from the Penguin Rookery sample (Table 3). Isolates A-85 and A-86 showed 98.73 and 98.41% ITS sequence similarity, respectively, to *Pseudogymnoascus roseus*. The occurrence of *Pseudogymnoascus* is ecologically relevant because members of this genus have been repeatedly reported from Antarctic soils and other cold terrestrial environments. The recovery of *P. roseus*-affiliated isolates in the present study is therefore consistent with previous observations of this lineage in Antarctic habitats and supports its association with cold terrestrial environments.

**Table 3:** Culturable fungal species isolated from soils in the Jutulsessen and Ahlmannryggen of Western Dronning Maud Land, Antarctica.

Sr. no.	Strain no.	Sample detail	Color of the colony	Medium	Accession no.	Closest homolog	Percent similarity
1	A-75	Roberts kollen	White	1/10PDA	MF467853	*Samsoniella alpina* YFCC 5818 (OQ476469)	99.65
2	A-77	Roberts kollen	Cream	1/10PDA	MF467854	*Samsoniella alpina* YFCC 5818 (OQ476469)	98.97
3	A-85	Roberts kollen	Blackish	PDA	MF467855	*Pseudogymnoascus roseus* CBS:395.65 (OR485022)	98.73
4	A-86	Roberts kollen	Brown	PDA	MF467856	*Pseudogymnoascus roseus* CBS:395.65 (OR485022)	98.41
5	A-87	Roberts kollen	Cream	PDA	MF467857	*Tolypocladium ovalisporum* CBS 700.92 (NR_155019)	96.41
6	A-88	Roberts kollen	Brownish	PDA	MF467858	*Leuconeurospora bharatiensis* isolate MCC-10126 (PQ304642)	100
7	A-108	Roberts kollen	White	1/10PDA	MF467859	*Tolypocladium ovalisporum* CBS 700.92 (NR_155019)	95.90
8	A-125	Roberts kollen	White	R2A	MF467860	*Tolypocladium ovalisporum* CBS 700.92 (NR_155019)	96.38
9	A-127	Roberts kollen	White	R2A	MF467861	*Tolypocladium ovalisporum* CBS 700.92 (NR_155019)	95.84
10	A-152	Penguin-1	Cream	R2A	MF467862	*Gelatinofungus brunneus* TNM GC 1703–31 (NR_175610.1)	99.43

Isolate A-88 exhibited 100% ITS sequence similarity to *Leuconeurospora bharatiensis*, a species previously documented from the Larsemann Hills region of Antarctica. This finding is particularly noteworthy because it indicates the occurrence of a *L. bharatiensis*-affiliated lineage in the geographically distinct Western Dronning Maud Land region. The result may suggest a broader Antarctic distribution of this taxon.

Four isolates (A-87, A-108, A-125 and A-127) were affiliated with *Tolypocladium ovalisporum*, showed ITS similarities ranging from 95.84 to 96.41%. Interestingly, a recent study reported an endolichenic *Tolypocladium* isolate from Fildes Peninsula, Antarctic Peninsula. Isolates A-75 and A-77 showed 99.65 and 98.97% ITS sequence similarity, respectively, to *Samsoniella alpina*, whereas isolate A-152 showed 99.43% similarity to *Gelatinofungus brunneus* (Table 3). These taxa have not been clearly established as characteristic Antarctic fungi. Their occurrence may reflect environmental persistence, dispersal, or association with local organic inputs rather than specific adaptation to Antarctic conditions.

Overall, the ITS-based analysis demonstrates that the culturable fungal assemblage of the investigated Antarctic habitats comprises several phylogenetically distinct lineages, including taxa with established associations with Antarctic and other cold environments. In particular, the recovery of *Pseudogymnoascus roseus, Leuconeurospora bharatiensis* and *Tolypocladium* sp., supports the presence of fungal lineages capable of persisting in cold Antarctic terrestrial habitats. The detection of *Samsoniella* and *Gelatinofungus* further expands the known culturable fungal diversity of the investigated sites. These fungi may contribute to decomposition and nutrient cycling in nutrient-limited WDML soils. The phylogenetic relationships among the fungal isolates were further evaluated using ITS-based phylogenetic analysis, and the resulting phylogenetic tree is presented in Figure 7. The topology therefore indicates that the fungal isolates recovered from the WDML nunataks are phylogenetically affiliated with lineages that occur in cold, polar, or otherwise extreme environments.

**Figure 7:**
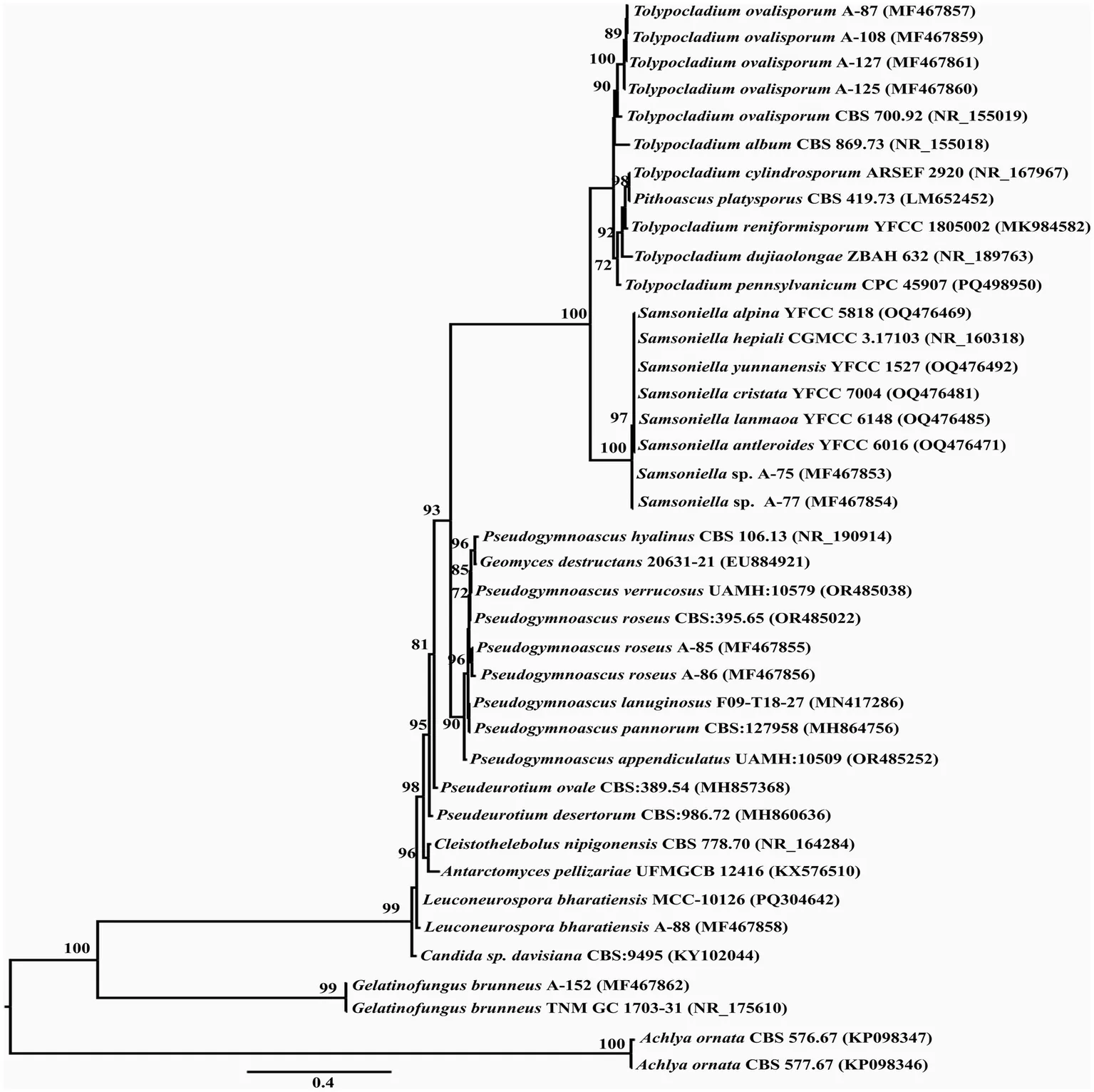
Phylogenetic tree diagram showing evolutionary relationships among various fungal species, with GenBank accession numbers indicated. Bootstrap values at nodes reflect statistical support. The scale bar represents genetic distance. Maximum-likelihood phylogenetic tree inferred using IQ-TREE v3.0.1. The phylogram was reconstructed from an alignment of 39 sequences of ITS gene region comprising 645 nucleotide positions, of which 419 were parsimony-informative, 29 were singleton sites, and 197 were constant sites (428 distinct site patterns). The best-fit nucleotide substitution model was found to be TN + G4, which was selected according to the Bayesian Information Criterion (BIC). The analysis incorporated a gamma-distributed rate variation among sites (shape parameter, α = 0.987) and an estimated proportion of invariable sites of 0.134. The estimated base frequencies were A = 0.231, C = 0.281, G = 0.250, and T = 0.238. Branch lengths are shown as substitutions per site. The log-likelihood of the consensus tree was −4383.252, with a total tree length of 4.645. Node labels show the ultrafast bootstrap support values, with values ≥70%.

### 3.5 Spatial pattern of microbial diversity across different nunataks

Bacterial species richness varied among the investigated nunataks and sampling locations (Figure 8). Based on the 16S rRNA gene-based identifications presented in Table 2, the highest species richness was recorded at Schumacherfjellet, where 10 bacterial species were identified. Valterkulten and Vassdalen each harbored six species, while Lorentzenpiggen contained five species. Eight species were identified from the Penguin rookery, whereas three species each were recorded from the Drill Hole, Flårjuven, Slettfjel, Robertskollen, and Vesleskarvet-Northern Buttress. Grunehogna showed the lowest species richness, with two species. The presence–absence pattern revealed a combination of widely distributed and site-associated bacterial taxa. *Dermacoccus nishinomiyaensis* was the most widely distributed species, occurring at Valterkulten, the Drill Hole, Flårjuven, Slettfjel, Schumacherfjellet, Lorentzenpiggen, Penguin rookery, and Vassdalen. In contrast, it was not detected at Grunehogna, Robertskollen, or Vesleskarvet-Northern Buttress. Only a small number of other taxa were shared among multiple locations. *Kocuria palustris* occurred at Valterkulten, Flårjuven, and Vesleskarvet-Northern Buttress. *Staphylococcus arlettae* was detected at Flårjuven and the Penguin rookery, while *Acinetobacter baumannii* occurred at Schumacherfjellet and the Penguin rookery. *Arthrobacter ginsengisoli* was shared between Schumacherfjellet and Robertskollen, whereas *Pseudarthrobacter phenanthrenivorans* occurred at Schumacherfjellet and Lorentzenpiggen. Several taxa were restricted to individual sampling locations within the present dataset. Valterkulten contained three site-restricted species, namely *Brevundimonas vesicularis*, *Stenotrophomonas pavanii*, and *Brachybacterium rhamnosum*. The Drill Hole contained two restricted taxa, *Sphingomonas sanguinis* and *Moraxella osloensis*. At Slettfjel, *Micrococcus yunnanensis* and *Staphylococcus gallinarum* were recorded only from this location. Schumacherfjellet exhibited the greatest species richness and contained several taxa that were not detected elsewhere in the dataset, including *Arthrobacter oxydans*, *Rothia* sp., *Pseudarthrobacter siccitolerans*, *Pseudomonas hunanensis*, and *Arthrobacter polychromogenes*. Other taxa at this site, including *Arthrobacter ginsengisoli*, *Microbacterium phyllosphaerae*, *Pseudarthrobacter phenanthrenivorans*, *Dermacoccus nishinomiyaensis*, and *Acinetobacter baumannii*, were also detected elsewhere. Grunehogna contained two species, *Aeromicrobium ginsengisoli* and *Arthrobacter agilis*, both of which were not detected at the other investigated locations. Lorentzenpiggen contained three site-restricted taxa, *Dietzia aerolata*, *Rhodococcus sovatensis*, and *Knoellia subterranea*, in addition to *Pseudarthrobacter phenanthrenivorans* and *Dermacoccus nishinomiyaensis*. At Robertskollen, *Arthrobacter sulfonivorans* and *Arthrobacter oryzae* were restricted to this location, whereas *Arthrobacter ginsengisoli* was shared with Schumacherfjellet. The Penguin rookery also harbored a distinct bacterial assemblage. Five taxa—*Paracoccus acridae*, *Lysinibacillus sphaericus*, *Kytococcus aerolatus*, *Methylobacterium rhodesianum*, and *Curtobacterium luteum*—were detected only at this location, while *Staphylococcus arlettae*, *Acinetobacter baumannii*, and *Dermacoccus nishinomiyaensis* were shared with other sampling sites. The Vesleskarvet-Northern Buttress samples contained three species: *Rhodococcus aerolatus*, *Sphingomonas daechungensis*, and *Kocuria palustris*. Of these, *R. aerolatus* and *S. daechungensis* were restricted to this sampling location in the present dataset. Vassdalen contained six species, comprising *Bacillus paramycoides*, *Bacillus altitudinis*, *Janibacter indicus*, *Bacillus cereus*, *Pontibacter amylolyticus*, and *Dermacoccus nishinomiyaensis*. Thus, the Vassdalen assemblage was compositionally distinct rather than species-poor relative to the other sites.

**Figure 8:**
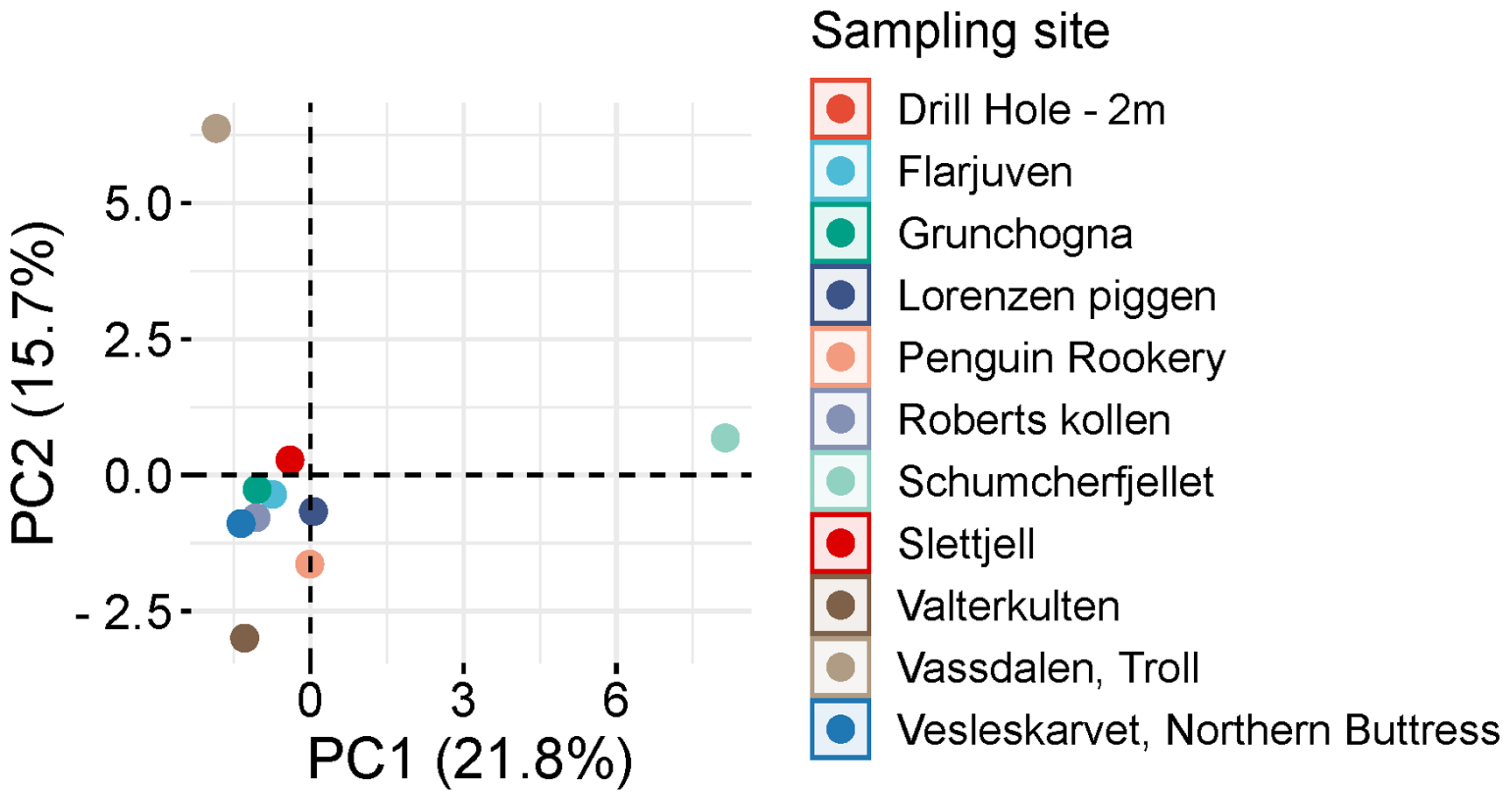
Scatter plot showing principal component analysis of sampling sites, with PC1 on the x-axis (21.8 percent variance) and PC2 on the y-axis (15.7 percent variance). Coloured dots represent ten sampling sites, with the legend identifying each by name and colour.

Overall, the distribution of culturable bacteria demonstrated pronounced spatial heterogeneity across the investigated nunataks. The coexistence of widely distributed taxa, particularly *Dermacoccus nishinomiyaensis*, with numerous location-restricted taxa suggests that bacterial community composition may be influenced by local environmental characteristics. Differences in substrate properties, sediment development, microhabitat conditions, exposure history, nutrient availability, and dispersal processes may contribute to the observed spatial pattern at WDML, Antarctica.

### 3.6 FAME analysis: fatty-acid composition and potential cold adaptation

FAME analysis of representative bacterial isolates revealed substantial variation in cellular fatty-acid composition (Supplementary Table 2). Saturated fatty acids accounted for 0.51–53.66% of the total fatty-acid profile, whereas iso-branched and anteiso-branched fatty acids represented 0.34–73.14% and 0.31–94.59%, respectively. Unsaturated fatty acids constituted 0.46–95.54% of the total profile. Overall, the isolates displayed variable combinations of branched-chain and unsaturated fatty acids, with increased proportions of one class often accompanied by reduced representation of another. The major branched-chain fatty acids detected included anteiso-C15:0, anteiso-C17:0, anteiso-C17:1, iso-C15:0, iso-C16:0 and iso-C16:1. Considerable variation in the composition of unsaturated fatty acids was also observed among the bacterial taxa. The cellular fatty-acid profiles of the 27 bacterial species investigated are presented in Supplementary Figure 2.

The FAME profiles indicated a pronounced contribution of branched-chain fatty acids, particularly anteiso- and iso-fatty acids, in several isolates. Notably, anteiso-C15:0 and anteiso-C17:0 were prominent in several *Arthrobacter* and related actinobacterial isolates, with anteiso-C15:0 accounting for approximately 30–64% of the total fatty-acid profile in several strains. Such enrichment in anteiso-branched fatty acids is consistent with a membrane homeoviscous adaptation strategy because methyl branching reduces the packing efficiency of acyl chains and can lower the temperature at which membrane lipids undergo a transition from a fluid to a more ordered state. Thus, the high abundance of branched-chain fatty acids observed in several WDML isolates may contribute to maintenance of membrane fluidity under low-temperature conditions, although their distribution is also influenced by bacterial taxonomy.

The data further indicate that Antarctic nunatak bacteria employ more than one membrane-modification strategy. In addition to iso/anteiso branching, modulation of unsaturation and fatty-acid chain composition may contribute to maintaining membrane functionality under environmental stress. The detection of C16:1 ω7c/ω6c, abundant anteiso-C17:0 and iso-C15:0 in several isolates provides evidence of variation in membrane lipid composition among taxa. Several *Arthrobacter* isolates exhibited total branched-chain fatty-acid contents of approximately 64–82%, while unsaturated fatty acids, including summed feature 3 [C16:1 ω7c/C16:1 ω6c], were also detected. The simultaneous occurrence of branched and unsaturated fatty acids suggests that different membrane-modification mechanisms may operate concurrently in some Antarctic terrestrial bacteria.

The Gram-negative isolates also showed characteristic differences in fatty-acid composition. The WDML *Pseudomonas* isolate contained appreciable proportions of unsaturated fatty acids, while other Gram-negative isolates showed substantial contributions from C16:0, C18:1 and summed feature 3. These observations are broadly consistent with reported fatty-acid profiles of Antarctic bacteria, in which genera such as *Pseudomonas* and *Polaromonas* commonly contain C16:1, C18:1n7c and C16:0, whereas *Psychrobacter* and *Acinetobacter* may contain substantial proportions of C16:1 and C18:1n9c. Such patterns indicate that cellular fatty-acid composition reflects both environmental selection and phylogenetic constraints.

From an ecological perspective, the observed membrane lipid plasticity may be particularly advantageous in Antarctic nunatak habitats, where microorganisms are exposed not only to low temperatures but also to repeated freeze–thaw cycles, desiccation, nutrient limitation, strong seasonal fluctuations and high irradiance. The capacity to modify membrane fatty-acid composition may therefore help maintain membrane integrity, fluidity and associated transport processes under fluctuating environmental conditions. Collectively, the FAME profiles of the WDML isolates provide evidence consistent with physiological flexibility in membrane composition and suggest that anteiso/iso branching and modulation of unsaturated fatty acids represent important components of the adaptive strategies of culturable Antarctic terrestrial bacteria.

## 4 Discussion

The present study characterized culturable bacterial and fungal communities inhabiting geographically isolated nunataks of Western Dronning Maud Land (WDML), Antarctica. The isolates exhibited a range of colony morphologies and pigmentation, indicating substantial phenotypic diversity. Such morphological and pigmentation diversity is consistent with previous investigations of culturable bacteria from Antarctic soils, rocks, ice and subglacial environments (Siebert and Hirsch, 1988). Most isolates grew between 1 and 22 °C, with an optimum of approximately 15 °C, a pattern closely comparable to Antarctic bacteria previously isolated from Lake Hoare and Taylor Glacier, indicating a predominantly cold-adapted culturable community (Clocksin et al., 2007). The ability of the present isolates to tolerate 1–7% NaCl is also reported for Antarctic bacteria (Goordial et al., 2016). Similarly, the preference of the isolates for near-neutral pH agrees with previous Antarctic studies in which many isolates showed optimal or restricted growth around pH 6–9 (Shivaji et al., 2013).

The physicochemical characteristics of the WDML nunatak soils further indicate a heterogeneous and nutrient-limited environment. Available nitrogen and phosphorus ranged from 16.97–49.98 ppm and 14.32–45.58 ppm, respectively, whereas organic carbon ranged from 0.89–1.83%. Such spatial variability is consistent with the pronounced heterogeneity reported for Antarctic soils at both microscale and landscape scales (Cocks et al., 1999; Chong et al., 2015). The comparatively higher organic-C concentrations in the present study than those reported for mineral soils of the McMurdo Dry Valleys (Barrett et al., 2007) may reflect local biological inputs, including mosses, lichens and bird-derived material, as reported from the Schirmacher Oasis (Singh et al., 2008). The enrichment of phosphorus at Vassdalen and Slettfjell and nitrogen at Grunehogna and Valterkulten further demonstrates strong spatial variation in nutrient availability. Such differences may arise from variations in weathering intensity, landscape age, microbial biomass, substrate characteristics and localized ornithogenic inputs. These observations agree with previous studies identifying organic carbon, moisture, substrate and landscape history as important controls of Antarctic microbial communities and nutrient cycling (Tytgat et al., 2016).

Considerable variation was also observed in the elemental composition of the nunatak soils. Although most elements occurred within the broad ranges reported for Antarctic soils and sediments (Lu et al., 2012), individual sites exhibited distinctive enrichment patterns. Elevated Cr, Ni and Co at Lorentzenpiggen, As at Robertskollen, Cu and Zn at Valterkulten and Robertskollen, and Cd, Tl, Pb, Cs, U, Li and Fe at Vassdalen indicate strong local influences of substrate mineralogy and sediment characteristics. Such geochemical heterogeneity may create localized differences in nutrient availability and metal/metalloid stress, thereby contributing to microbial colonization and community assembly. The contrasting elemental signatures therefore appear to reflect predominantly local geochemical and lithological characteristics, with possible contributions from anthropogenic sources (Trevizani et al., 2022).

Taxonomic analysis demonstrated that the WDML nunataks harbor a diverse culturable bacterial assemblage. MALDI-TOF MS generated reproducible protein fingerprints for many isolates and was particularly informative for closely related Actinobacterial taxa, including *Arthrobacter* and *Dermacoccus*. However, only 13 isolates could be identified to species level using the MALDI BioTyper database, highlighting the limitations of databases developed primarily for clinical microorganisms. Whereas MALDI-TOF MS can show high concordance with 16S rRNA-based identification for clinical bacteria (Alispahic et al., 2010; Sogawa et al., 2011; Timperio et al., 2017), substantially lower concordance has been reported for environmental microorganisms because Antarctic and other environmental taxa are poorly represented in databases (Dieckmann et al., 2005; Viver et al., 2015; Oberbeckmann et al., 2011; Uhlik et al., 2011; Mulec et al., 2015). The present study therefore demonstrates the value of combining MALDI-TOF MS with 16S rRNA sequencing and also contributes additional spectral profiles that may assist future identification of Antarctic environmental bacteria (Kopcakova et al., 2014; Santos et al., 2016). Similar applications of MALDI-TOF MS have been reported for Antarctic bacteria from the Sør Rondane Mountains, James Ross Island and Antarctic freshwater systems (Tahon and Willems, 2017; Pantucek et al., 2018; Sedláček et al., 2021), while recent studies have also highlighted the need for expansion of reference libraries for Antarctic environmental isolates (Sedláček et al., 2022; Snopková et al., 2025). The unidentified MALDI-TOF MS spectra generated in the present study were assigned taxonomic identities based on the corresponding 16S rRNA gene sequence analyses, thereby compensating for the limited representation of environmental bacterial genera and species in the MALDI BioTyper database (Supplementary Figure 1). Thus, the characteristic spectra generated in the present study not only support the taxonomic characterization of the WDML isolates but may also provide valuable reference spectra for future identification of Antarctic bacterial diversity. MALDI-TOF data suggest that, despite the geographical separation between Western Dronning Maud Land and SørRondane Mountains, there is some taxonomic overlap among culturable Antarctic terrestrial bacteria (*Brevundimonas* and *Sphingomonas*). This is consistent with the idea that exposed Antarctic soils and rock surfaces impose strong environmental selection favoring a relatively restricted set of stress-tolerant bacterial lineages.

The 16S rRNA analysis identified 37 bacterial species belonging mainly to Actinobacteria, Firmicutes and Proteobacteria. The predominance of *Arthrobacter* and related Actinobacterial taxa agrees with previous investigations of Antarctic terrestrial environments (Cheng and Foght, 2007; Yu et al., 2010; Karolien et al., 2011; Staebe et al., 2019). Several other genera detected in the present study, including *Acinetobacter, Bacillus, Brevundimonas, Kocuria, Methylobacterium, Pseudomonas, Rhodococcus, Sphingomonas* and *Staphylococcus*, have also been reported from different Antarctic regions (Peeters et al., 2011; Bajerski and Wagner 2013; Zdanowski et al., 2013; Zhao et al., 2016; González-Rocha et al., 2017; Cong et al., 2020; Almela et al., 2021). The overlap in genera among geographically distant Antarctic regions suggests that similar environmental pressures may favor the persistence of a relatively restricted set of stress-tolerant bacterial lineages. However, the occurrence of common taxa does not indicate uniform microbial communities, as Antarctic microbial assemblages remain strongly structured by local environmental conditions (Yergeau et al., 2009; Lee et al., 2012; Almela et al., 2021; Fernández-Martínez et al., 2021).

Importantly, the WDML nunataks also showed clear local differentiation in their culturable bacterial assemblages. Differences among sites may reflect variation in nutrient availability, bedrock and mineralogy, moisture, exposure history, geographic isolation and biological inputs (Savaglia et al., 2024). This interpretation is consistent with previous studies showing differentiation between Jutulsessen/Ahlmannryggen and Sør Rondane despite their occurrence within Dronning Maud Land (Staebe et al., 2019; Obbels et al., 2016). Site-specific patterns further support this ecological interpretation. Valterkulten was characterized by repeated recovery of *Arthrobacter flavus* together with *Brevundimonas, Dermacoccus, Stenotrophomonas, Brachybacterium* and *Kocuria*, whereas Schumacherfjellet contained a broader assemblage including *Arthrobacter, Microbacterium, Pseudarthrobacter, Pseudomonas, Acinetobacter* and *Dermacoccus*. The recovery of *Dietzia*, *Pseudarthrobacter*, *Rhodococcus*, *Knoellia* and *Dermacoccus* from Lorenzenpiggen indicates a strong Actinobacterial signature, while Robertskollen was dominated by *Arthrobacter*-related taxa. In contrast, the Penguin Rookery assemblage included *Paracoccus, Lysinibacillus, Staphylococcus, Acinetobacter, Kytococcus, Methylobacterium* and *Curtobacterium*, potentially reflecting the influence of seabird-derived organic and nutrient inputs. Similar differences associated with penguin and seal influence have been reported from Antarctic coastal environments (Chong et al., 2010). The occurrence of *Bacillus* species at Vassdalen is also ecologically relevant because endospore formation may enhance persistence under desiccation, freeze–thaw cycles and prolonged nutrient limitation.

The fungal component of the study was represented by *Samsoniella, Gelatinofungus, Leuconeurospora, Pseudogymnoascus* and *Tolypocladium*. The recovery of these genera indicates that fungi form an additional component of the culturable microbial assemblage of WDML nunataks. In particular, the identification of *Pseudogymnoascus roseus* is consistent with previous observations from Deception Island, South Shetland Islands, Antarctica. (Rosa et al., 2020). The occurrence of *Leuconeurospora bharatiensis* is also of particular interest because this species has been reported from Larsemann Hills, continental Antarctic environments (Jiya et al., 2024). A recent study reported an endolichenic *Tolypocladium* isolate from Fildes Peninsula, Antarctica (Li et al., 2026). The occurrence of these taxa in the present samples therefore provides further evidence for the persistence of cold-associated fungal lineages in Antarctic terrestrial ecosystems. The other fungal taxa, *Gelatinofungus* and *Samsoniella* have not been clearly established as characteristic components of Antarctic fungal communities based on currently available reports. Nevertheless, their detection in the present study expands the known culturable fungal diversity of the investigated sites. Their occurrence may reflect environmental persistence or passive dispersal.

Finally, cellular fatty-acid profiles provided additional evidence for physiological adaptation among the WDML bacterial isolates. The 27 isolates examined showed substantial proportions of branched-chain fatty acids, particularly anteiso-C15:0, anteiso-C17:0, anteiso-C17:1, iso-C15:0, iso-C16:0 and iso-C16:1. This pattern agrees with previous studies of Antarctic bacteria, including isolates from Heywood Lake sediment (Rotert et al., 1993; Fukunaga and Russel, 1990). The coexistence of branched and unsaturated fatty acids is consistent with homeoviscous adaptation, in which changes in membrane lipid composition help maintain membrane fluidity and function under low-temperature conditions. Similar combinations of increased unsaturation and branched-chain fatty-acid synthesis have been reported in cold-adapted bacteria such as *Flavobacterium* (Králová, 2017) and in other psychrotolerant microorganisms (Nishida and Murata, 1996; Chintalapati et al., 2004; Prakash et al., 2014; Mező et al., 2022; Akulava et al., 2024). The observed fatty-acid profiles therefore support the interpretation that membrane lipid plasticity contributes to bacterial persistence under the low-temperature and fluctuating conditions of Antarctic nunatak environments.

## 5 Conclusion

The present study provides original insights into the culturable microbial diversity inhabiting the nunataks of Western Dronning Maud Land (WDML), Antarctica, a unique and relatively poorly explored terrestrial ecosystem. By combining culture-based isolation with MALDI BioTyper (MALDI-TOF MS), 16S rRNA gene sequencing, ITS sequencing, and fatty acid profiling, this study provides an integrated assessment of bacterial and fungal diversity and physiological characteristics associated with survival in these extreme environments. The investigated nunatak soils exhibited considerable spatial heterogeneity in their physicochemical characteristics, reflecting differences in local environmental conditions and providing a range of ecological niches for microbial colonization.

The culturable bacterial community comprised diverse taxa, with recurrent occurrence of *Arthrobacter*, *Dermacoccus*, *Kocuria*, *Microbacterium*, and related actinobacterial genera. Their frequent detection across the investigated sites suggests that these groups are well suited to the exposed, oligotrophic, and environmentally fluctuating conditions of Antarctic terrestrial habitats. At the same time, the occurrence of *Pseudomonas*, *Sphingomonas*, *Bacillus*, *Rhodococcus*, *Acinetobacter*, *Methylobacterium*, and other bacterial genera demonstrates that microbial persistence in nunatak environments is supported by diverse physiological and ecological strategies. The heterogeneous distribution of taxa among sampling sites further suggests that substrate availability, moisture, nutrient status, microclimate, exposure history, and other local environmental factors contribute to the observed patterns of microbial occurrence. The diverse MALDI-TOF MS protein fingerprints generated in this study represent useful reference data for future identification of Antarctic environmental bacteria and may contribute to improving the representation of Antarctic taxa in MALDI-TOF MS databases. The fungal isolates further expanded the culture-based inventory of microorganisms from Antarctic nunataks, with representatives affiliated with *Samsoniella*, *Tolypocladium*, *Pseudogymnoascus*, *Leuconeurospora*, and *Gelatinofungus*. The detection of *Leuconeurospora* and *Pseudogymnoascus*is particularly noteworthy because this species has previously been described from Antarctica. Their occurrence contributes to the documented culturable fungal diversity of the investigated sites and emphasizes the need for further investigation of fungal dispersal, persistence, and ecological functioning in isolated Antarctic terrestrial habitats.

Physiological characterization and fatty acid profiling provided additional evidence of mechanisms potentially supporting microbial persistence under cold and nutrient-limited conditions. The predominance of branched-chain fatty acids, particularly anteiso-C15:0 and anteiso-C17:0, in several Gram-positive isolates, together with the occurrence of unsaturated fatty acids and summed features in Gram-negative isolates, is consistent with membrane lipid remodeling and homeoviscous regulation under low-temperature conditions. These lipid profiles suggest that membrane flexibility is an important component of the physiological strategies employed by bacteria inhabiting the WDML nunataks.

Overall, the present study establishes a valuable baseline for understanding the culturable microbial ecology of WDML nunataks and demonstrates that these remote and oligotrophic habitats harbor a diverse assemblage of bacteria and fungi with distinct taxonomic and physiological characteristics. The findings extend the available culture-based knowledge of Antarctic nunatak microbiota and provide a foundation for future comparative biogeographical, genomic, and functional studies of microbial adaptation and ecological functioning in extreme environments. The combination of low temperature, aridity, nutrient limitation, high irradiance, and strong environmental fluctuations also makes Antarctic nunataks valuable terrestrial analogs for investigating microbial survival under Mars-like environmental stresses. Future genomic and metabolomics studies will be important for resolving the mechanisms underlying microbial persistence, adaptation, and ecological functioning in these extreme Antarctic ecosystems.

## Data Availability

The datasets presented in this study can be found in online repositories. The names of the repository/repositories and accession number(s) can be found in the article/Supplementary material.
