# Supplementary material for: Microbial diversity and adaptation Strategies in the nunataks of Western Dronning Maud Land, Antarctica
Source: Front Microbiol. 2026 Oct 22;17:1921961. doi: 10.3389/fmicb.2026.1921961 (PMC13736047; doi:10.3389/fmicb.2026.1921961)
Supplement: Supplementary file 1 [file Data_Sheet_1.PDF]

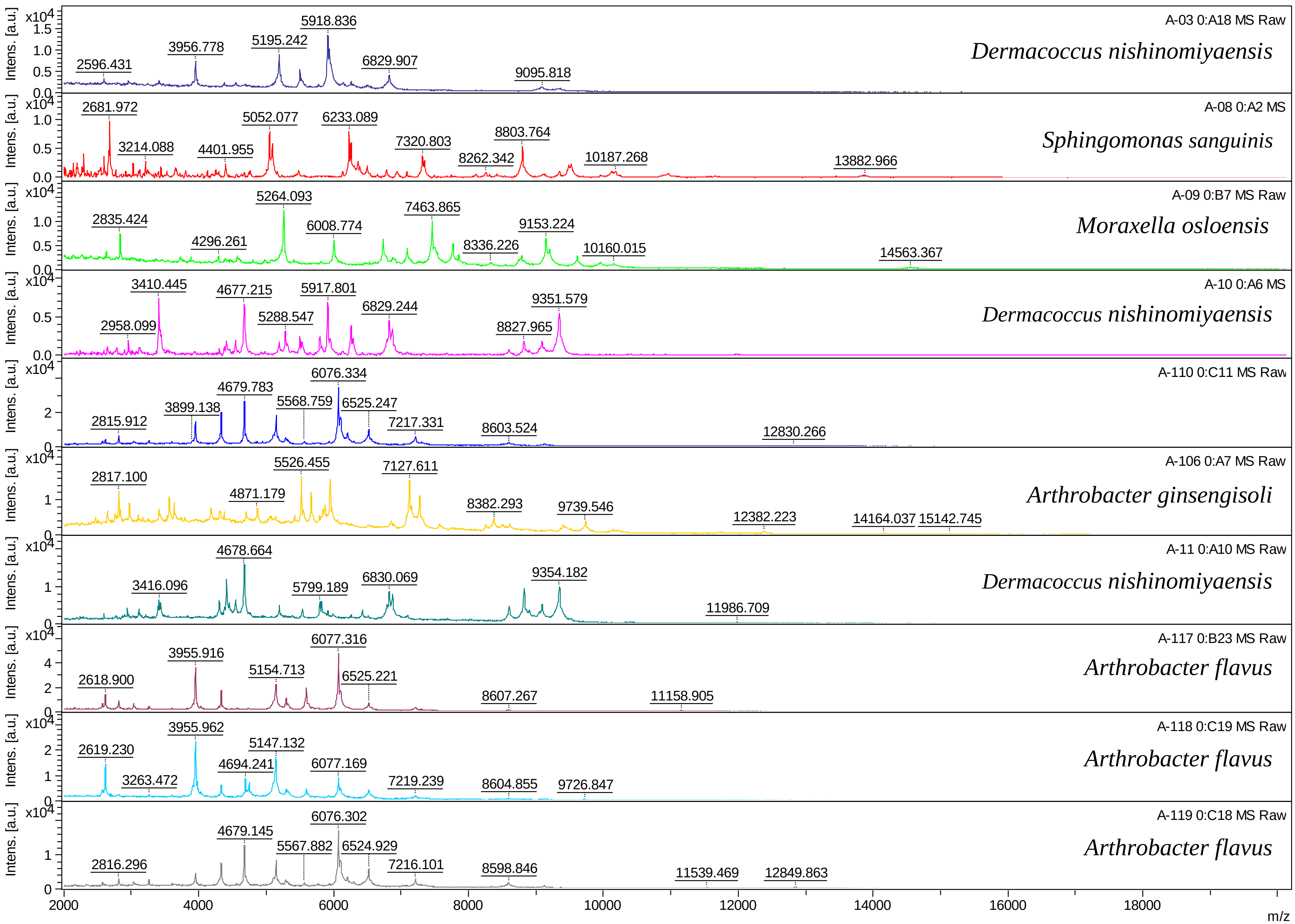

A-03 0:A18 MS Raw
Dermacoccus nishinomiyaensis
2596.431
3956.778
5195.242
5918.836
6829.907
9095.818
A-08 0:A2 MS
Sphingomonas sanguinis
2681.972
3214.088
4401.955
5052.077
6233.089
7320.803
8262.342
8803.764
10187.268
13882.966
A-09 0:B7 MS Raw
Moraxella osloensis
2835.424
4296.261
5264.093
6008.774
7463.865
8336.226
9153.224
10160.015
14563.367
A-10 0:A6 MS
Dermacoccus nishinomiyaensis
2958.099
3410.445
4677.215
5288.547
5917.801
6829.244
8827.965
9351.579
A-110 0:C11 MS Raw
2815.912
3899.138
4679.783
5568.759
6076.334
6525.247
7217.331
8603.524
12830.266
A-106 0:A7 MS Raw
Arthrobacter ginsengisoli
2817.100
4871.179
5526.455
7127.611
8382.293
9739.546
12382.223
14164.037
15142.745
A-11 0:A10 MS Raw
Dermacoccus nishinomiyaensis
3416.096
4678.664
5799.189
6830.069
9354.182
11986.709
A-117 0:B23 MS Raw
Arthrobacter flavus
2618.900
3955.916
5154.713
6077.316
6525.221
8607.267
11158.905
A-118 0:C19 MS Raw
Arthrobacter flavus
2619.230
3263.472
3955.962
4694.241
5147.132
6077.169
7219.239
8604.855
9726.847
A-119 0:C18 MS Raw
Arthrobacter flavus
2816.296
4679.145
5567.882
6076.302
6524.929
7216.101
8598.846
11539.469
12849.863
Intens. [a.u.]
x10^4
m/z
2000
4000
6000
8000
10000
12000
14000
16000
18000

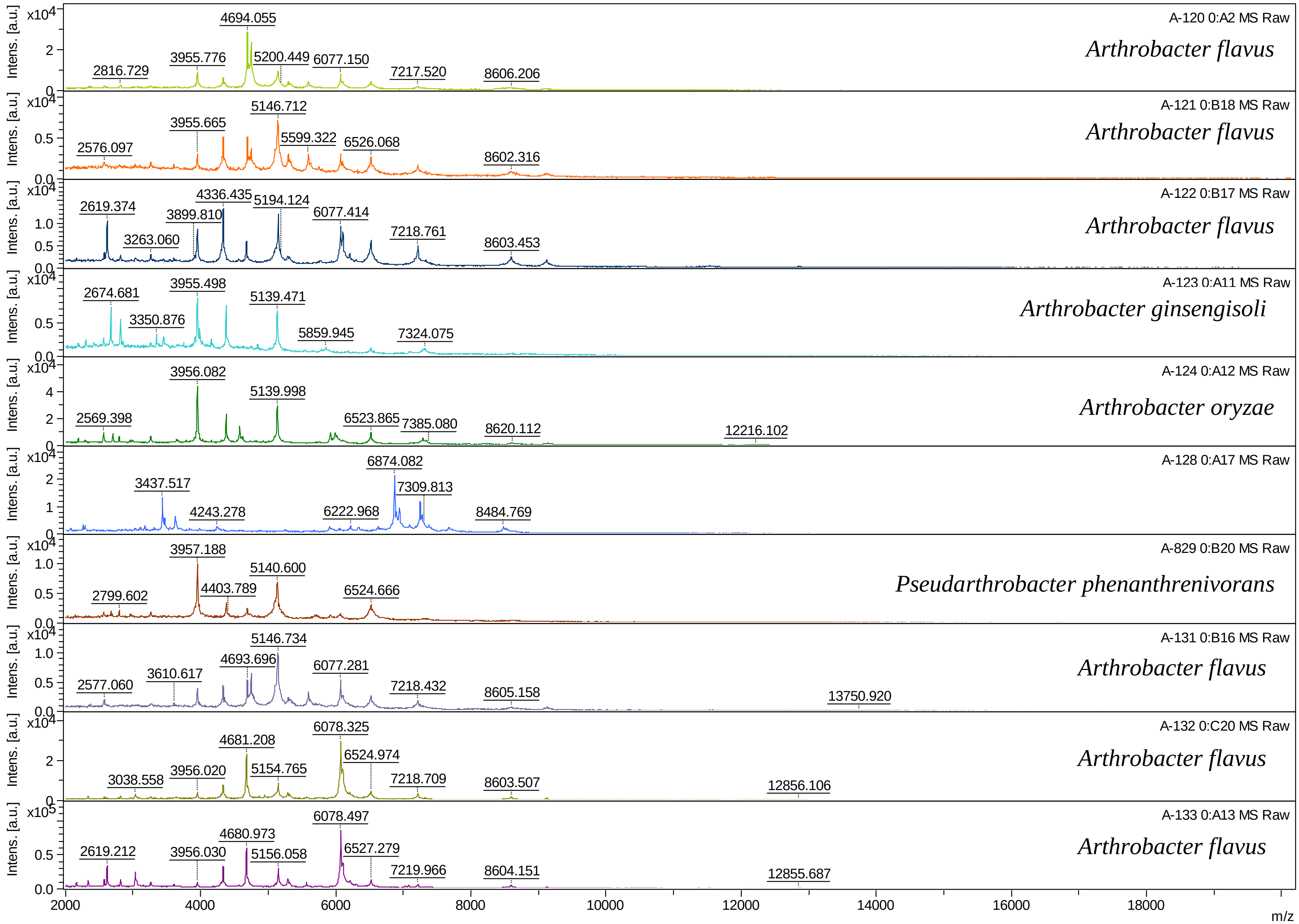

A-120 0:A2 MS Raw
Arthrobacter flavus
4694.055
3955.776
5200.449
6077.150
2816.729
7217.520
8606.206
A-121 0:B18 MS Raw
Arthrobacter flavus
5146.712
3955.665
5599.322
6526.068
2576.097
8602.316
A-122 0:B17 MS Raw
Arthrobacter flavus
4336.435
2619.374
5194.124
3899.810
6077.414
7218.761
3263.060
8603.453
A-123 0:A11 MS Raw
Arthrobacter ginsengisoli
2674.681
3955.498
5139.471
3350.876
5859.945
7324.075
A-124 0:A12 MS Raw
Arthrobacter oryzae
3956.082
5139.998
6523.865
7385.080
2569.398
8620.112
12216.102
A-128 0:A17 MS Raw
6874.082
3437.517
7309.813
4243.278
6222.968
8484.769
A-829 0:B20 MS Raw
Pseudarthrobacter phenanthrenivorans
3957.188
5140.600
4403.789
6524.666
2799.602
A-131 0:B16 MS Raw
Arthrobacter flavus
5146.734
4693.696
6077.281
3610.617
2577.060
7218.432
8605.158
13750.920
A-132 0:C20 MS Raw
Arthrobacter flavus
6078.325
4681.208
6524.974
3956.020
5154.765
3038.558
7218.709
8603.507
12856.106
A-133 0:A13 MS Raw
Arthrobacter flavus
6078.497
4680.973
2619.212
6527.279
3956.030
5156.058
7219.966
8604.151
12855.687
Intens. [a.u.]
x10^4
x10^5
m/z
2000
4000
6000
8000
10000
12000
14000
16000
18000

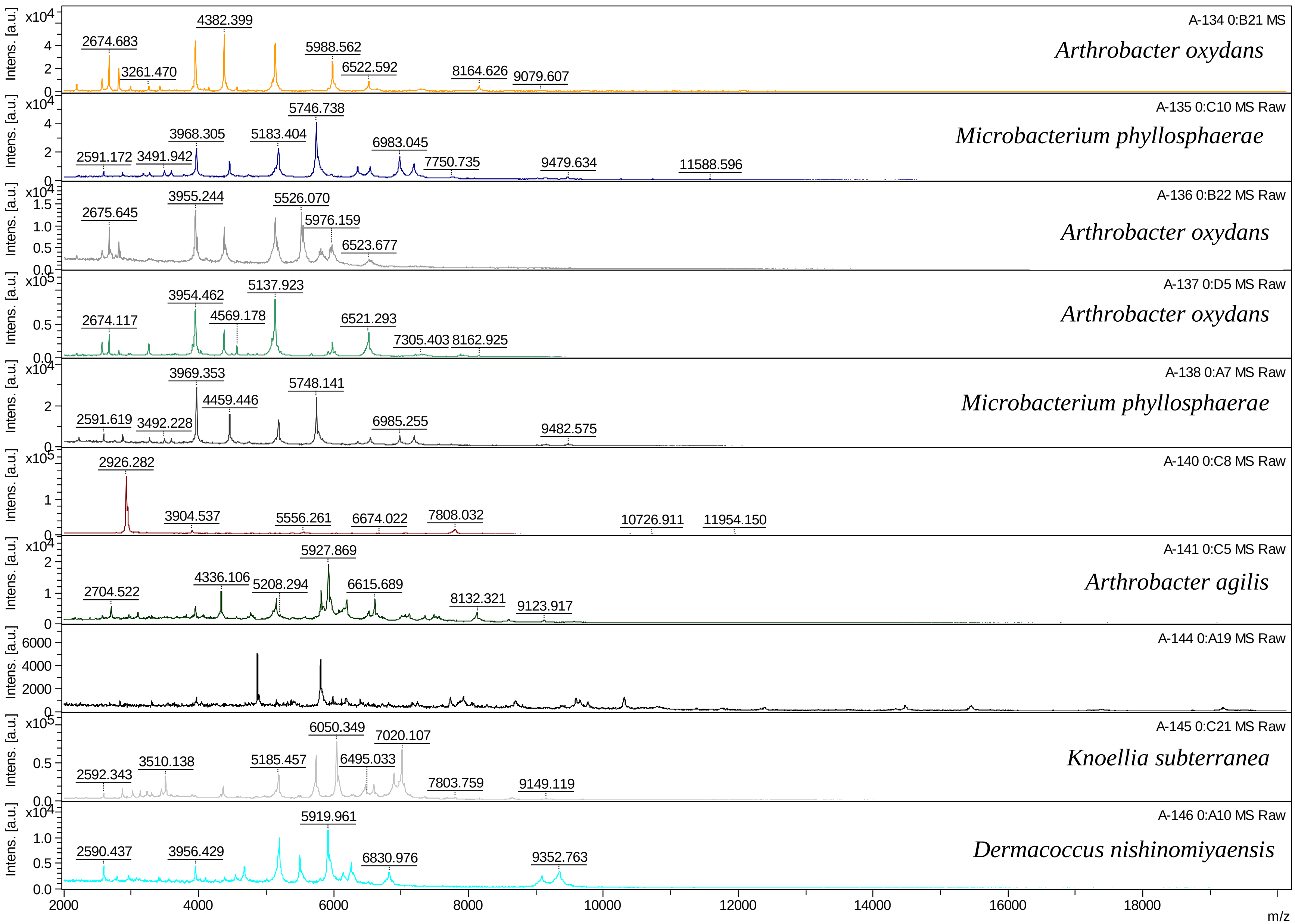

A-134 0:B21 MS
Arthrobacter oxydans
2674.683
3261.470
4382.399
5988.562
6522.592
8164.626
9079.607
A-135 0:C10 MS Raw
Microbacterium phyllosphaerae
2591.172
3491.942
3968.305
5183.404
5746.738
6983.045
7750.735
9479.634
11588.596
A-136 0:B22 MS Raw
Arthrobacter oxydans
2675.645
3955.244
5526.070
5976.159
6523.677
A-137 0:D5 MS Raw
Arthrobacter oxydans
2674.117
3954.462
4569.178
5137.923
6521.293
7305.403
8162.925
A-138 0:A7 MS Raw
Microbacterium phyllosphaerae
2591.619
3492.228
3969.353
4459.446
5748.141
6985.255
9482.575
A-140 0:C8 MS Raw
2926.282
3904.537
5556.261
6674.022
7808.032
10726.911
11954.150
A-141 0:C5 MS Raw
Arthrobacter agilis
2704.522
4336.106
5208.294
5927.869
6615.689
8132.321
9123.917
A-144 0:A19 MS Raw
A-145 0:C21 MS Raw
Knoellia subterranea
2592.343
3510.138
5185.457
6050.349
6495.033
7020.107
7803.759
9149.119
A-146 0:A10 MS Raw
Dermacoccus nishinomiyaensis
2590.437
3956.429
5919.961
6830.976
9352.763
Intens. [a.u.]
m/z
2000
4000
6000
8000
10000
12000
14000
16000
18000

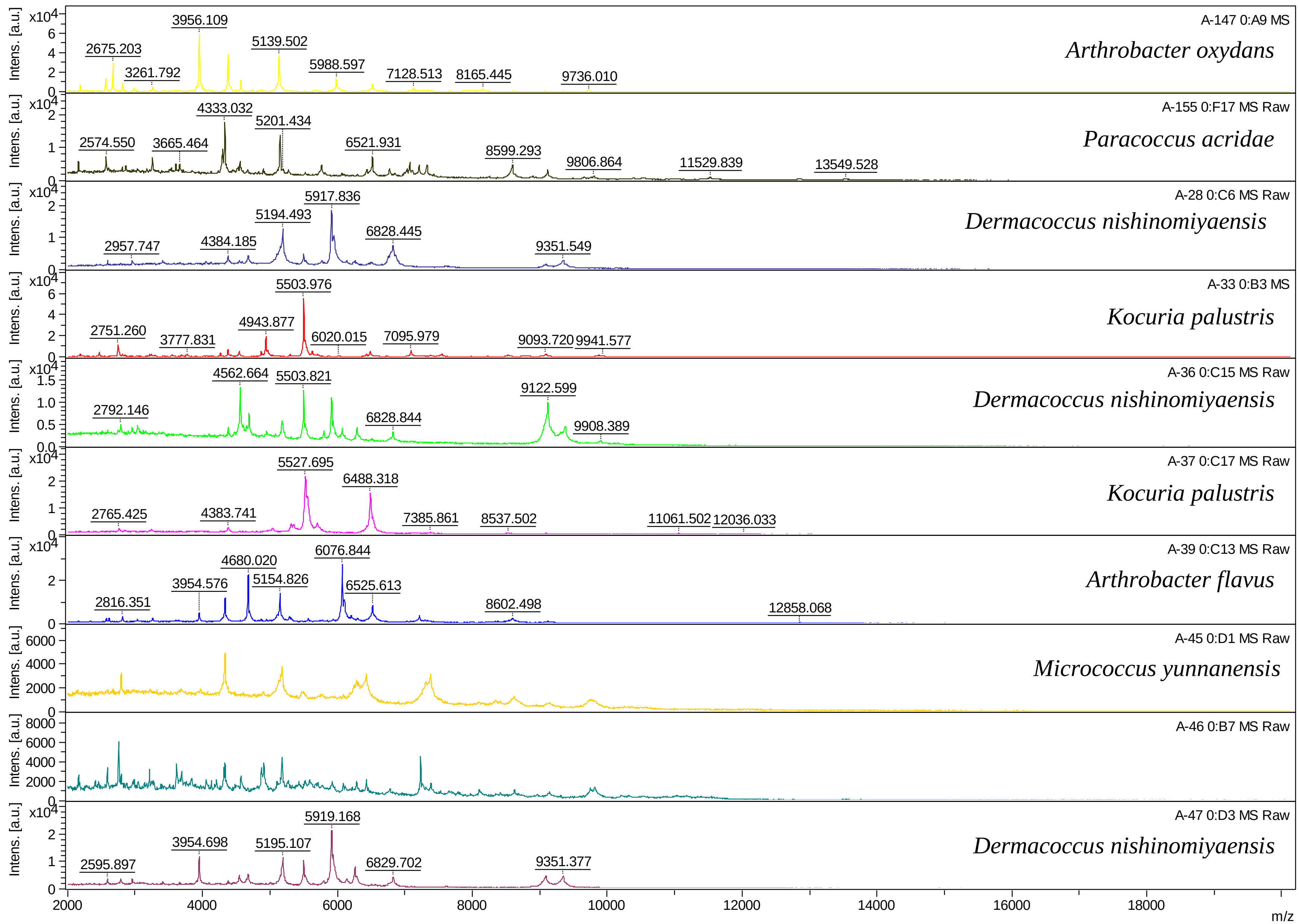

A-147 0:A9 MS
Arthrobacter oxydans
2675.203
3261.792
3956.109
5139.502
5988.597
7128.513
8165.445
9736.010
A-155 0:F17 MS Raw
Paracoccus acridae
2574.550
3665.464
4333.032
5201.434
6521.931
8599.293
9806.864
11529.839
13549.528
A-28 0:C6 MS Raw
Dermacoccus nishinomiyaensis
2957.747
4384.185
5194.493
5917.836
6828.445
9351.549
A-33 0:B3 MS
Kocuria palustris
2751.260
3777.831
4943.877
5503.976
6020.015
7095.979
9093.720
9941.577
A-36 0:C15 MS Raw
Dermacoccus nishinomiyaensis
2792.146
4562.664
5503.821
6828.844
9122.599
9908.389
A-37 0:C17 MS Raw
Kocuria palustris
2765.425
4383.741
5527.695
6488.318
7385.861
8537.502
11061.502
12036.033
A-39 0:C13 MS Raw
Arthrobacter flavus
2816.351
3954.576
4680.020
5154.826
6076.844
6525.613
8602.498
12858.068
A-45 0:D1 MS Raw
Micrococcus yunnanensis
A-46 0:B7 MS Raw
A-47 0:D3 MS Raw
Dermacoccus nishinomiyaensis
2595.897
3954.698
5195.107
5919.168
6829.702
9351.377
Intens. [a.u.]
x10^4
m/z
2000
4000
6000
8000
10000
12000
14000
16000
18000

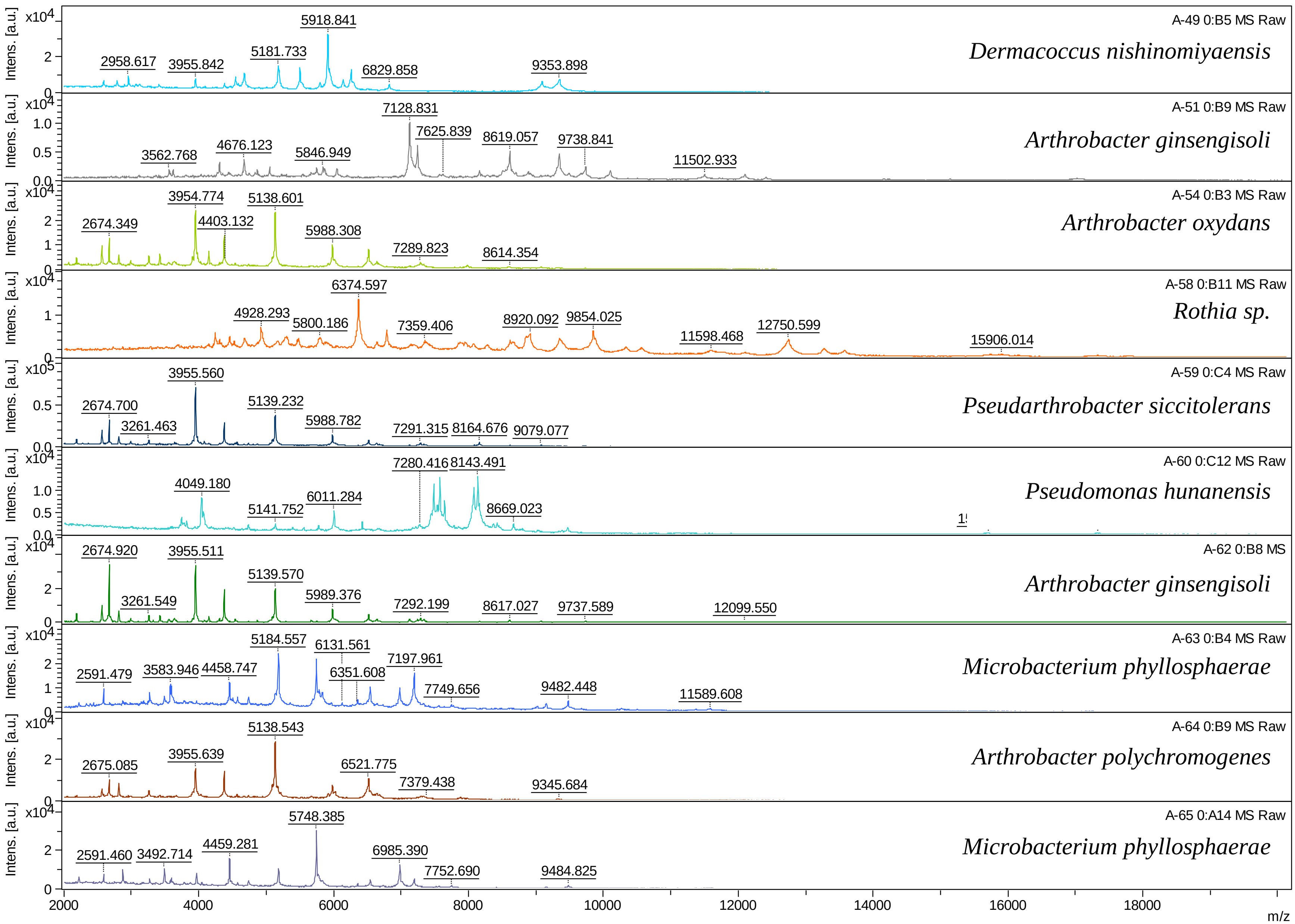

A-49 0:B5 MS Raw
Dermacoccus nishinomiyaensis
2958.617
3955.842
5181.733
5918.841
6829.858
9353.898
A-51 0:B9 MS Raw
Arthrobacter ginsengisoli
3562.768
4676.123
5846.949
7128.831
7625.839
8619.057
9738.841
11502.933
A-54 0:B3 MS Raw
Arthrobacter oxydans
2674.349
3954.774
4403.132
5138.601
5988.308
7289.823
8614.354
A-58 0:B11 MS Raw
Rothia sp.
4928.293
5800.186
6374.597
7359.406
8920.092
9854.025
11598.468
12750.599
15906.014
A-59 0:C4 MS Raw
Pseudarthrobacter siccitolerans
2674.700
3261.463
3955.560
5139.232
5988.782
7291.315
8164.676
9079.077
A-60 0:C12 MS Raw
Pseudomonas hunanensis
4049.180
5141.752
6011.284
7280.416
8143.491
8669.023
A-62 0:B8 MS
Arthrobacter ginsengisoli
2674.920
3261.549
3955.511
5139.570
5989.376
7292.199
8617.027
9737.589
12099.550
A-63 0:B4 MS Raw
Microbacterium phyllosphaerae
2591.479
3583.946
4458.747
5184.557
6131.561
6351.608
7197.961
7749.656
9482.448
11589.608
A-64 0:B9 MS Raw
Arthrobacter polychromogenes
2675.085
3955.639
5138.543
6521.775
7379.438
9345.684
A-65 0:A14 MS Raw
Microbacterium phyllosphaerae
2591.460
3492.714
4459.281
5748.385
6985.390
7752.690
9484.825
Intens. [a.u.]
m/z
2000
4000
6000
8000
10000
12000
14000
16000
18000

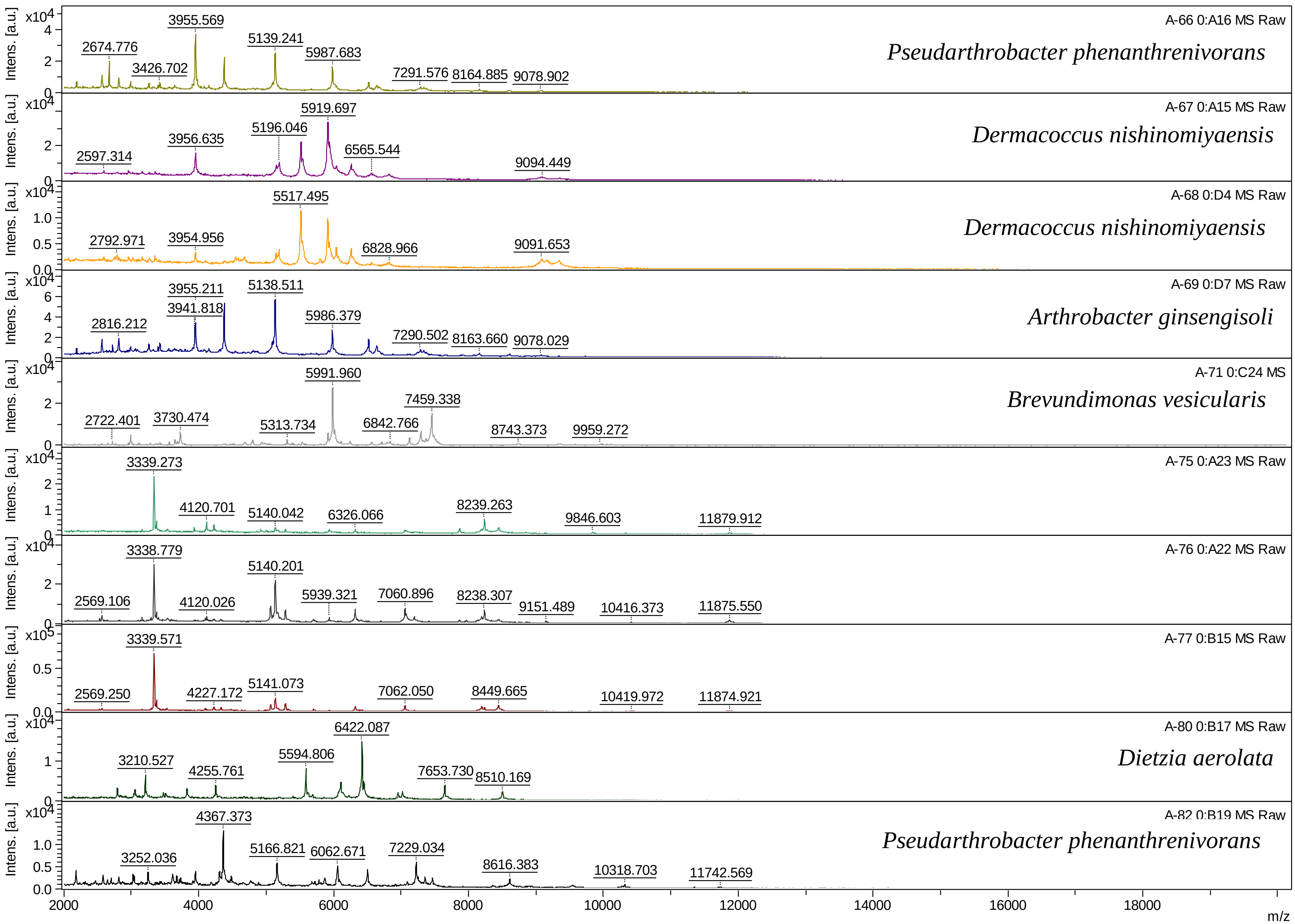
A-66 0:A16 MS Raw
Pseudarthrobacter phenanthrenivorans
2674.776
3955.569
3426.702
5139.241
5987.683
7291.576
8164.885
9078.902
A-67 0:A15 MS Raw
Dermacoccus nishinomiyaensis
5919.697
5196.046
3956.635
2597.314
6565.544
9094.449
A-68 0:D4 MS Raw
Dermacoccus nishinomiyaensis
5517.495
2792.971
3954.956
6828.966
9091.653
A-69 0:D7 MS Raw
Arthrobacter ginsengisoli
3955.211
5138.511
3941.818
2816.212
5986.379
7290.502
8163.660
9078.029
A-71 0:C24 MS
Brevundimonas vesicularis
5991.960
7459.338
2722.401
3730.474
5313.734
6842.766
8743.373
9959.272
A-75 0:A23 MS Raw
3339.273
4120.701
5140.042
6326.066
8239.263
9846.603
11879.912
A-76 0:A22 MS Raw
3338.779
5140.201
2569.106
4120.026
5939.321
7060.896
8238.307
9151.489
10416.373
11875.550
A-77 0:B15 MS Raw
3339.571
2569.250
4227.172
5141.073
7062.050
8449.665
10419.972
11874.921
A-80 0:B17 MS Raw
Dietzia aerolata
6422.087
3210.527
4255.761
5594.806
7653.730
8510.169
A-82 0:B19 MS Raw
Pseudarthrobacter phenanthrenivorans
4367.373
3252.036
5166.821
6062.671
7229.034
8616.383
10318.703
11742.569
Intens. [a.u.]
x10^4
x10^5
m/z
2000
4000
6000
8000
10000
12000
14000
16000
18000

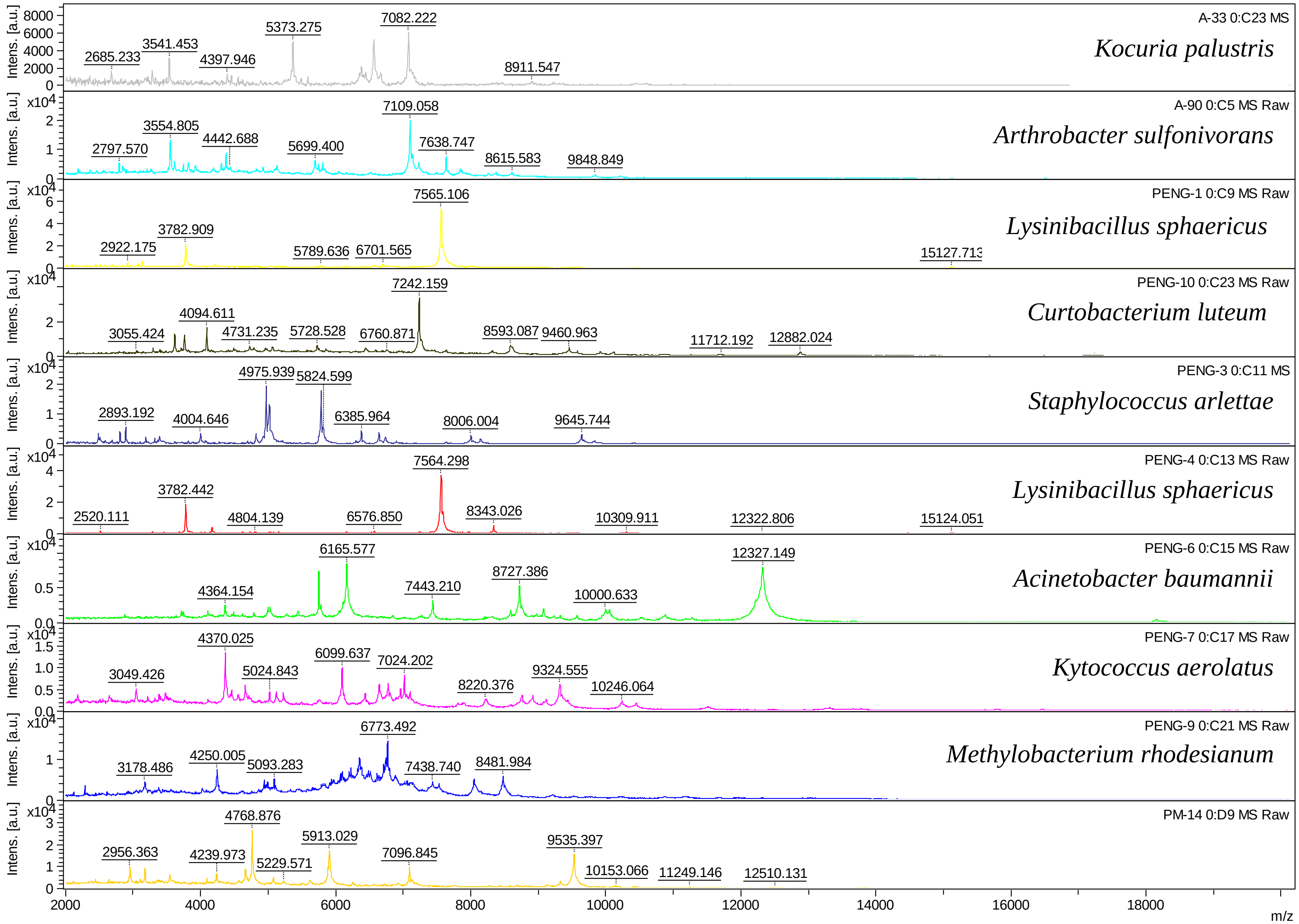

Supplementary Figure 1, showing Characteristic and unique MALDI TOF Mass Spectra of Bacterial species isolated from Western Dronning Maud Land, Antarctica.
