# Supplementary material for: Microbial diversity and adaptation Strategies in the nunataks of Western Dronning Maud Land, Antarctica
Source: Front Microbiol. 2026 Oct 22;17:1921961. doi: 10.3389/fmicb.2026.1921961 (PMC13736047; doi:10.3389/fmicb.2026.1921961)

1. FAME_*Arthrobacter flavus* A-39

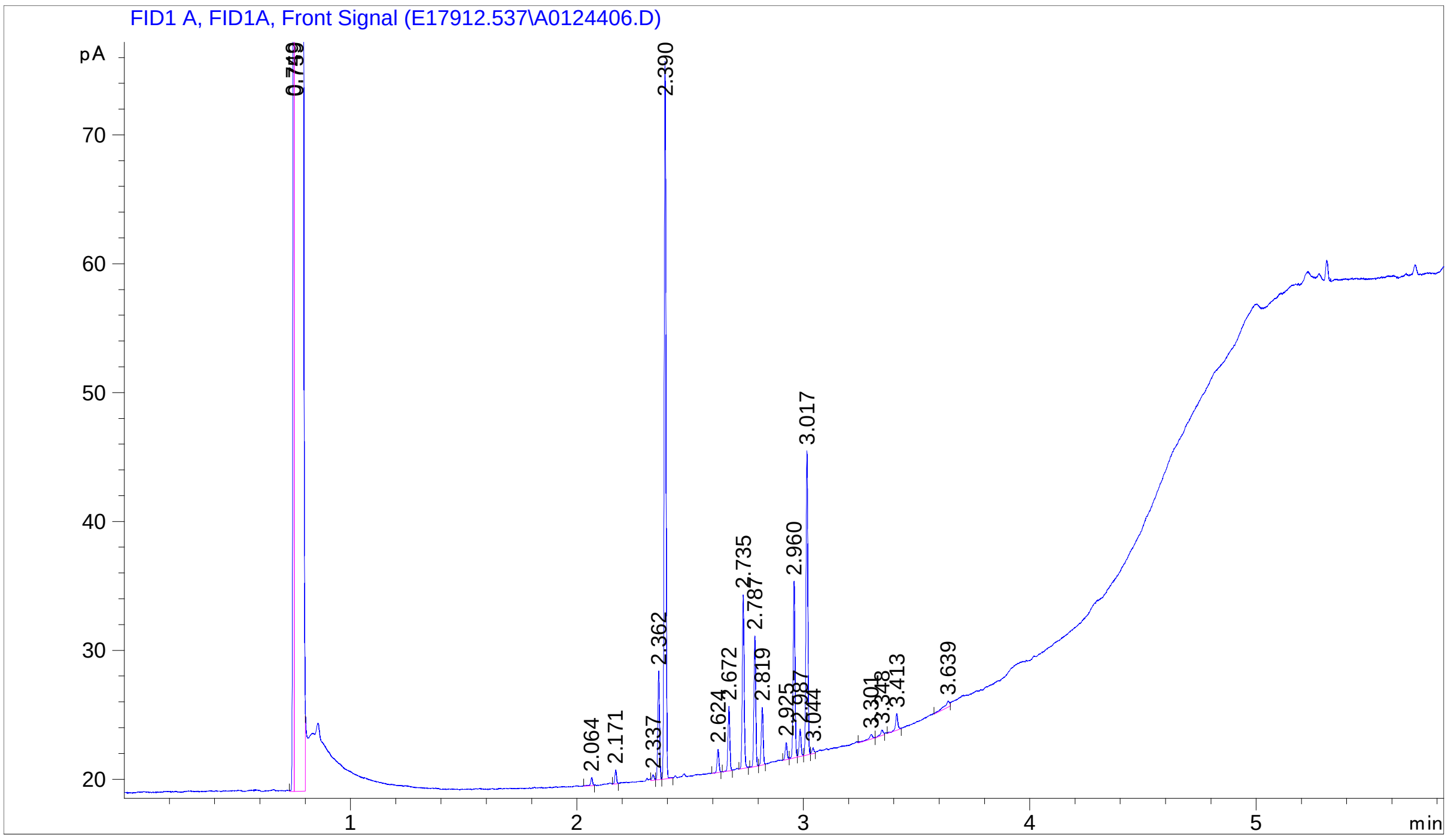

2. FAME_*Arthrobacter oxydans* A-50

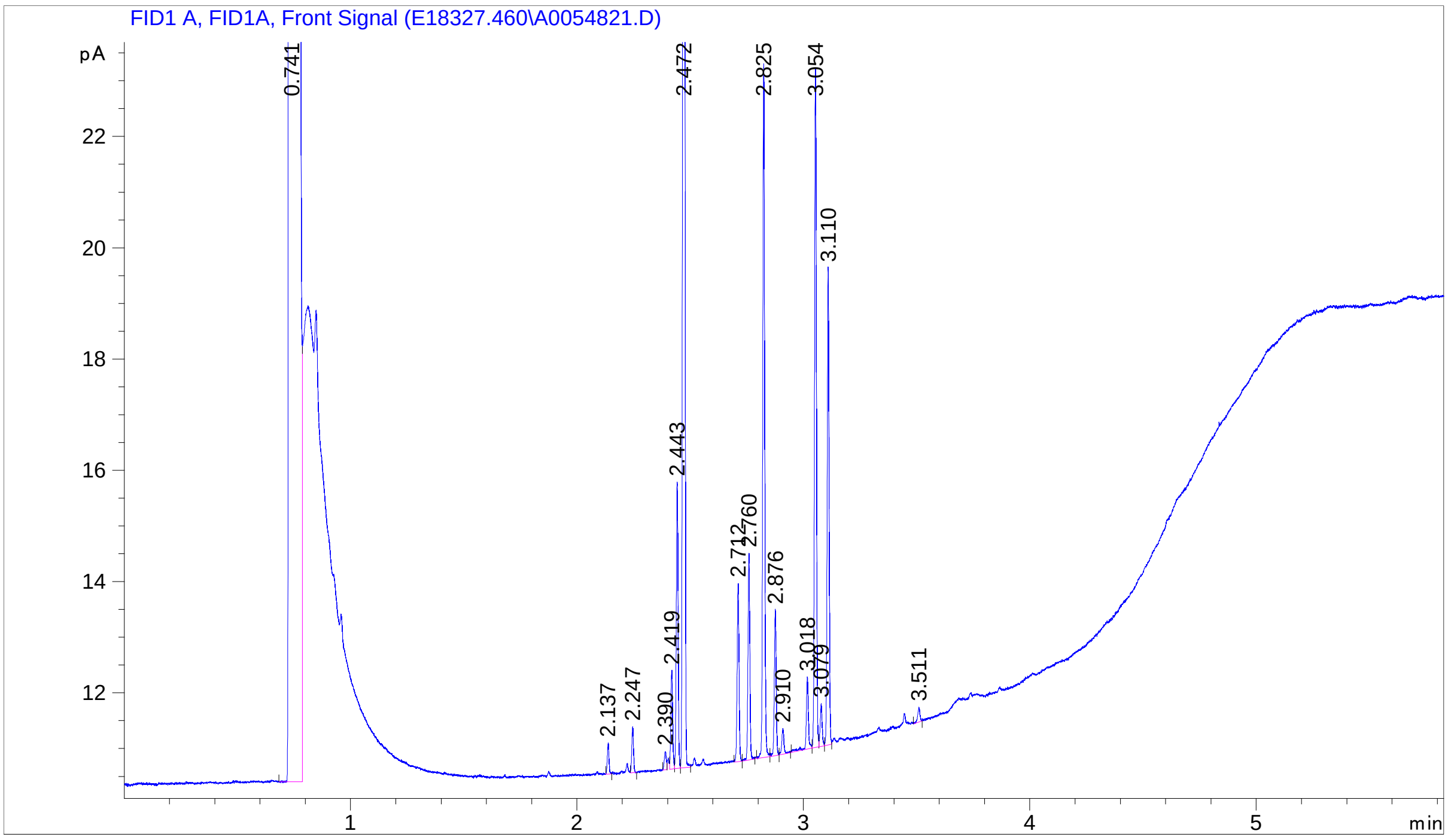

3. FAME_*Arthrobacter ginsengisoli*_A-51

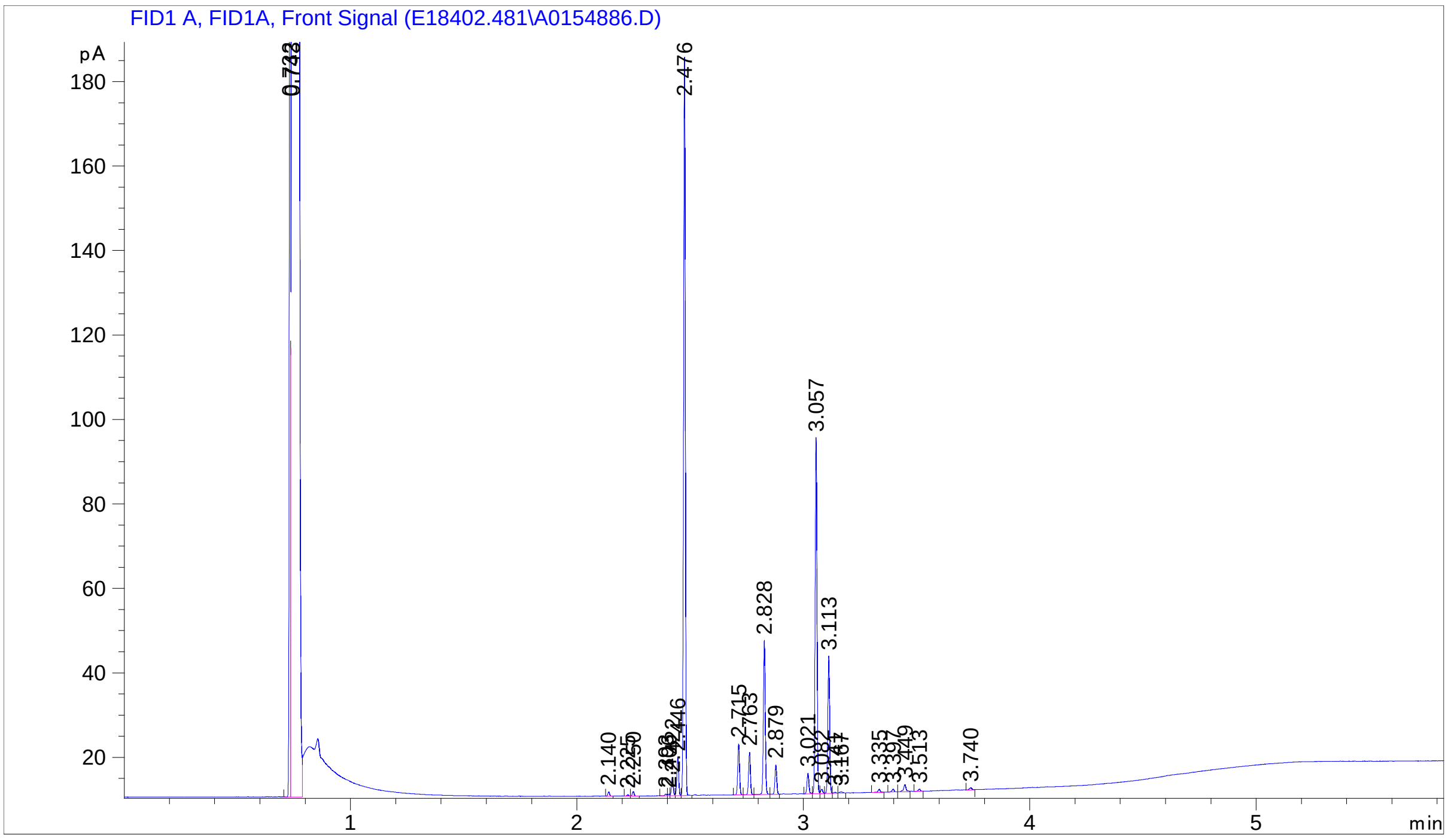

4. FAME_*Arthrobacter sulfonivorans* _A-90

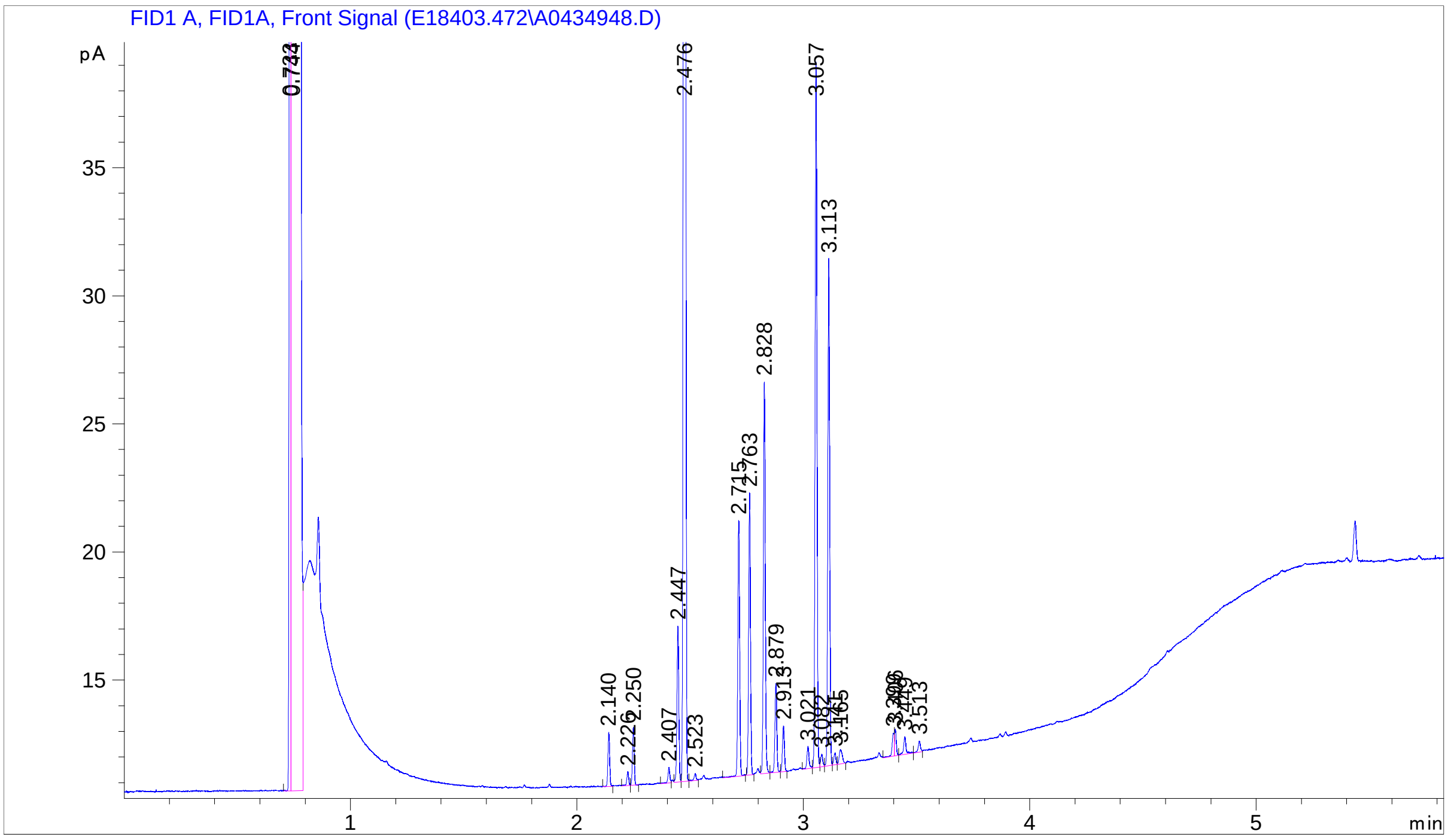

5. FAME_*Arthrobacter agilis* A-141

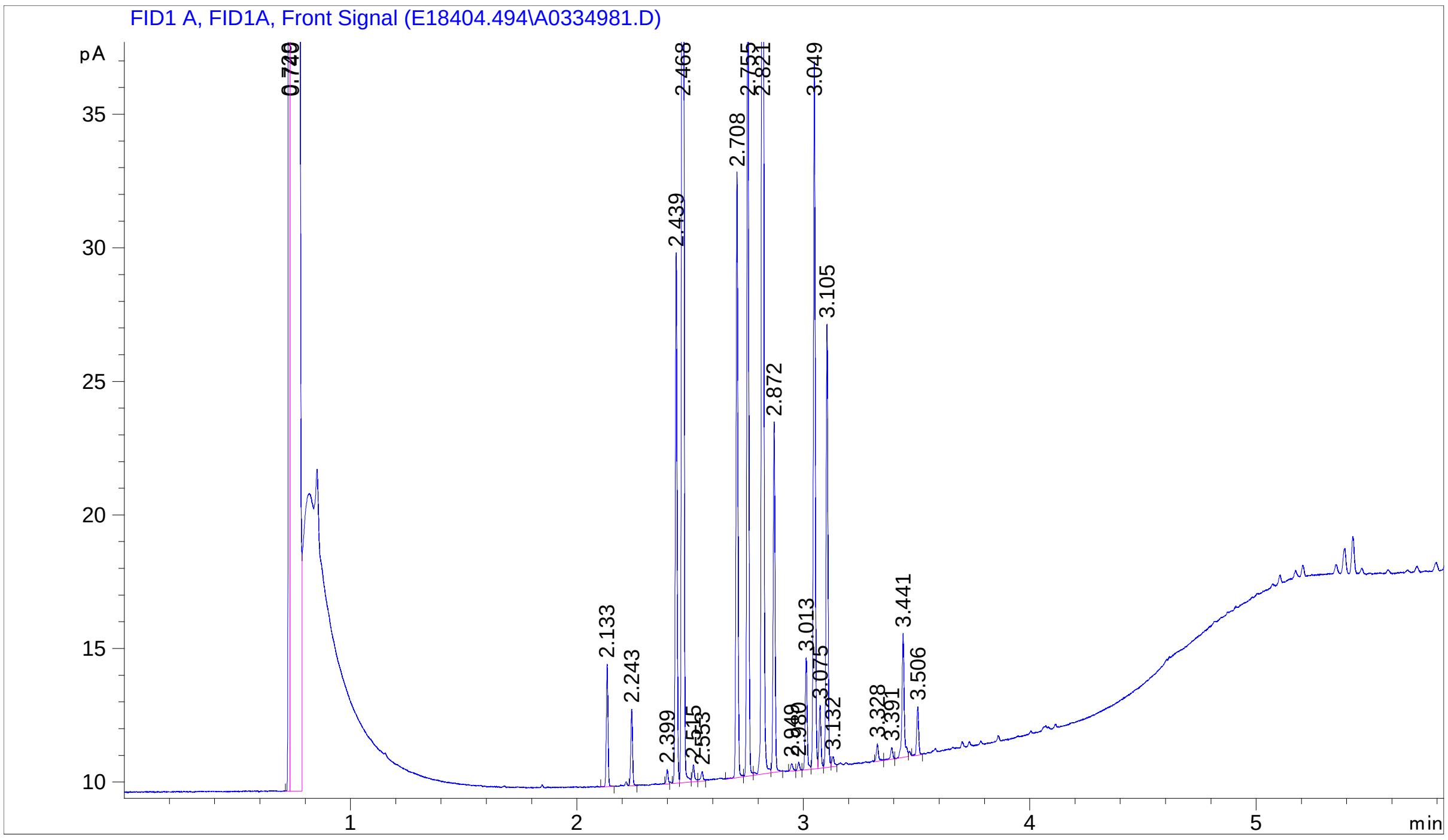

6. FAME_ *Kocuria palustris* A-33

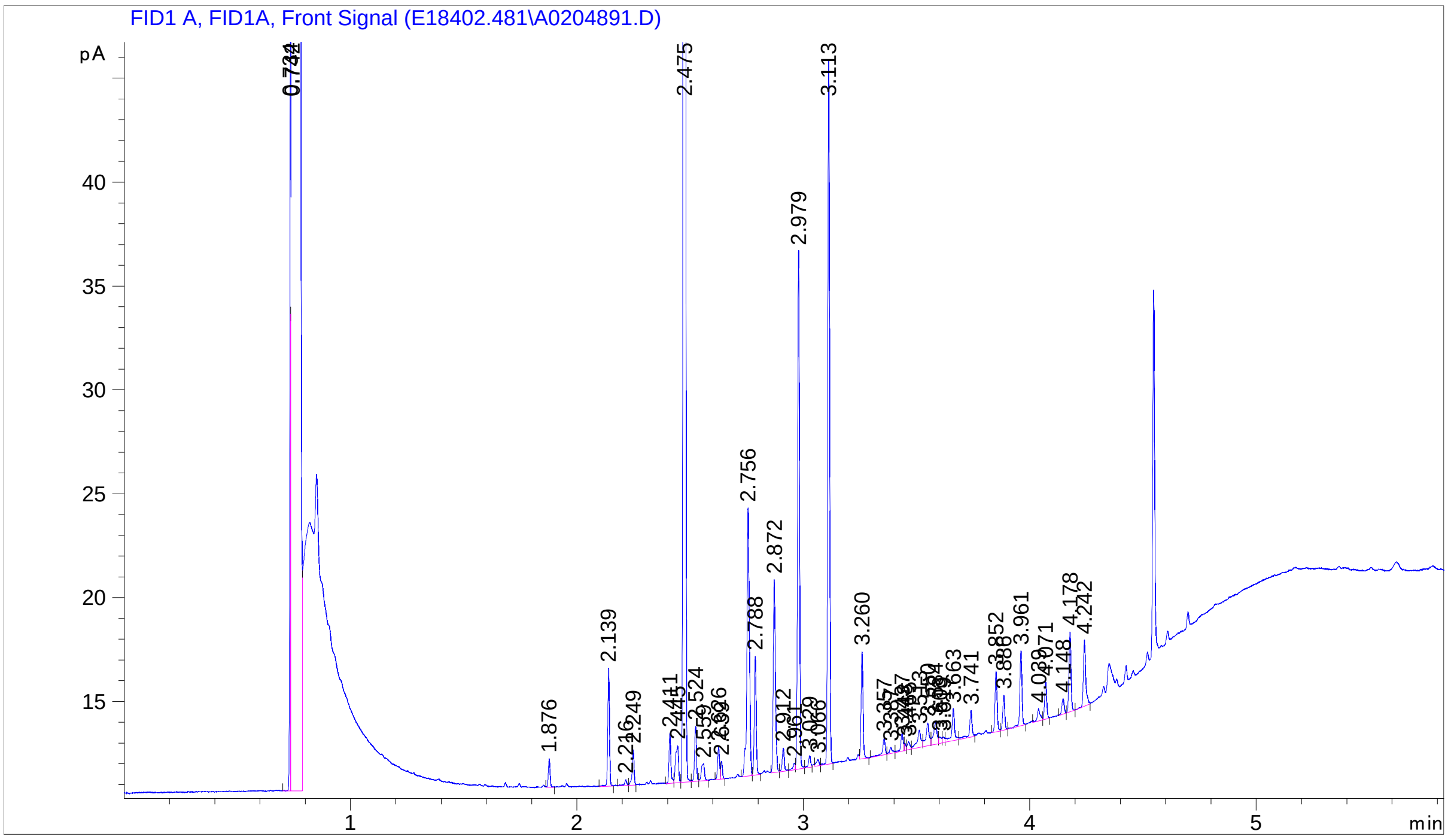

7. FAME_ *Kytococcus aerolatus* Peng-7

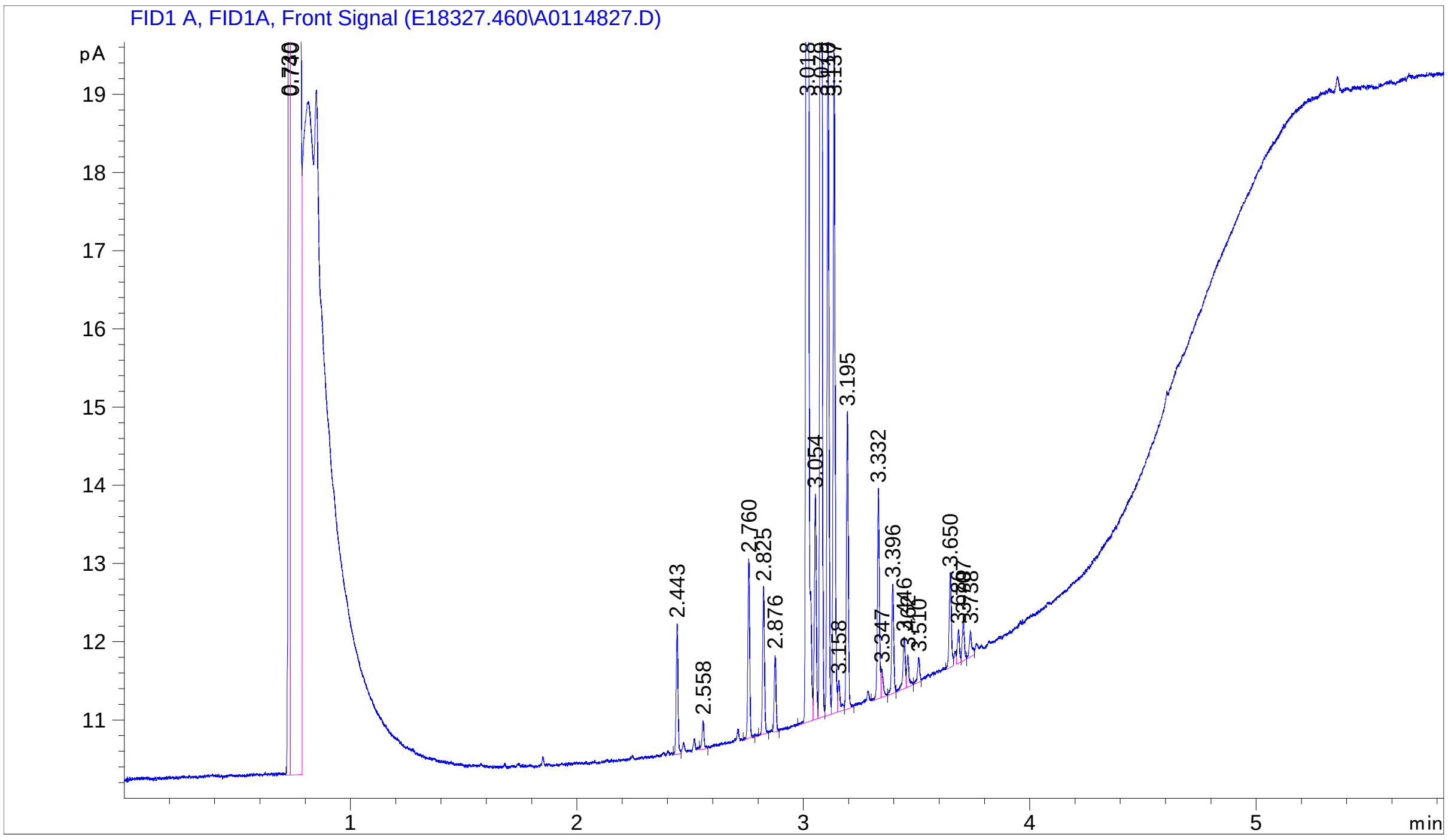

8. FAME_ *Pseudarthrobacter siccitolerans* A-59

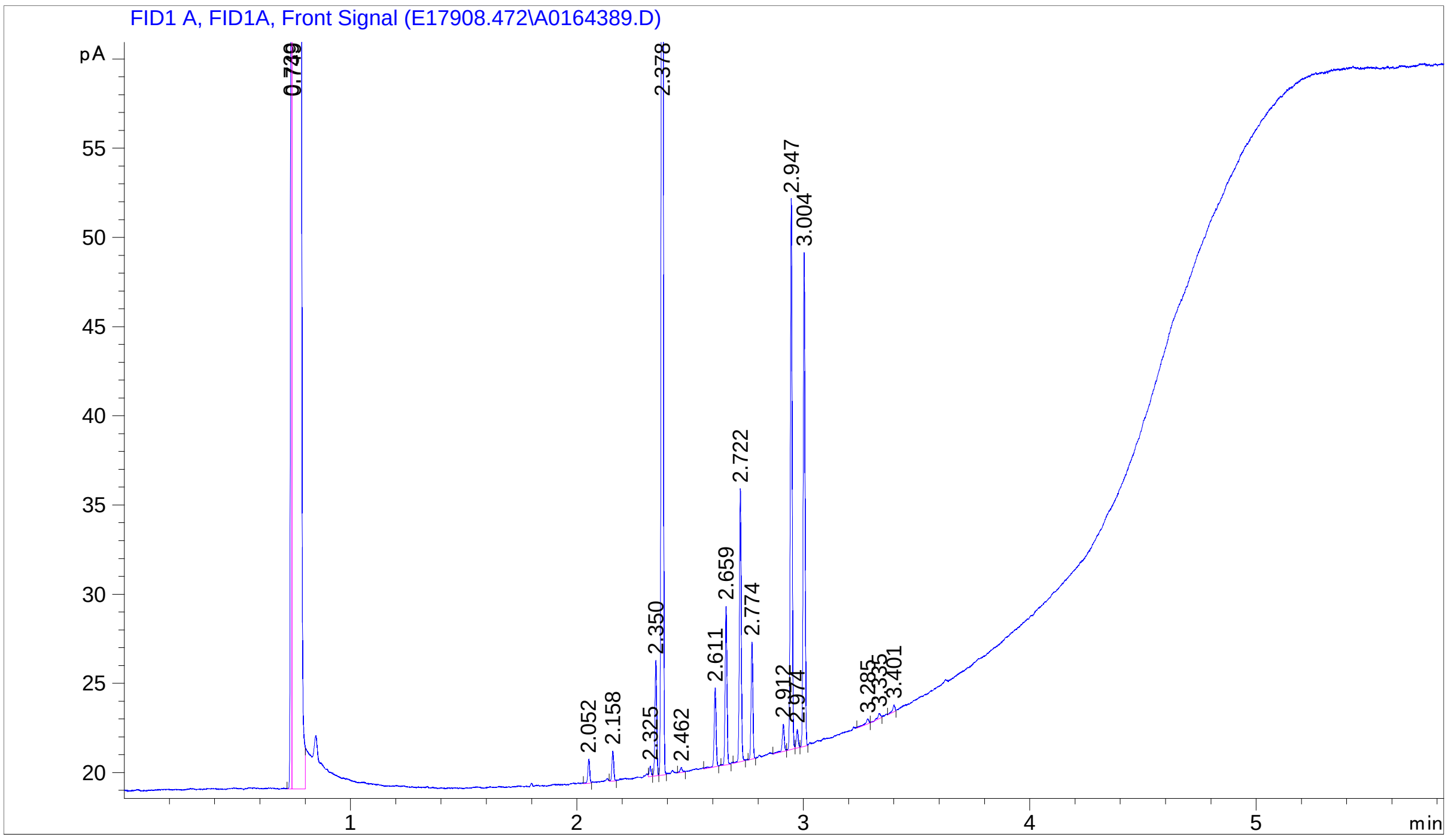

9. FAME_ *Stenotrophomonas pavanii* A-6

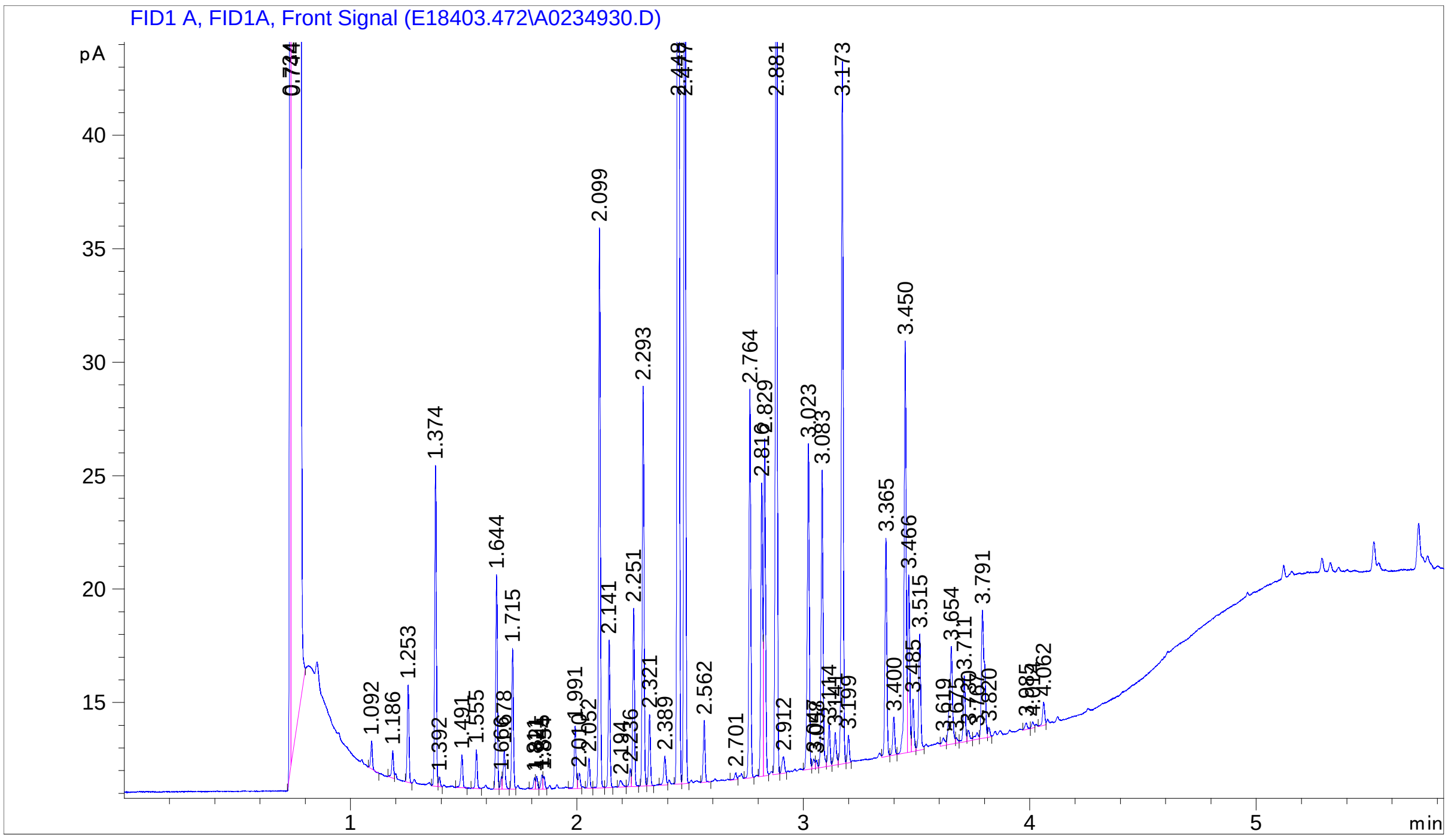

10. FAME_*Staphylococcus arlettae* Peng 3

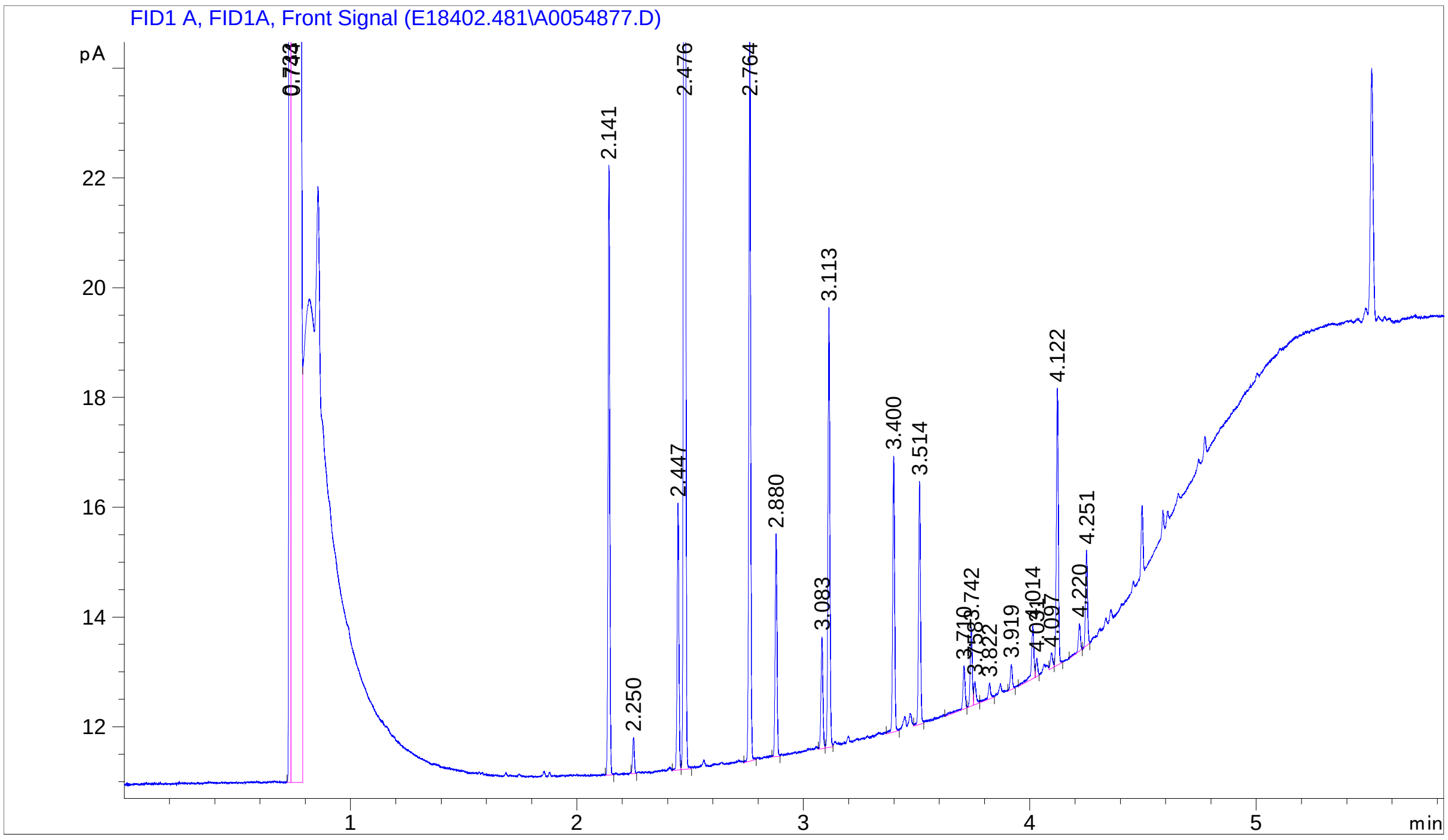

11. FAME_*Aeromicrobium ginsengisoli* A-72

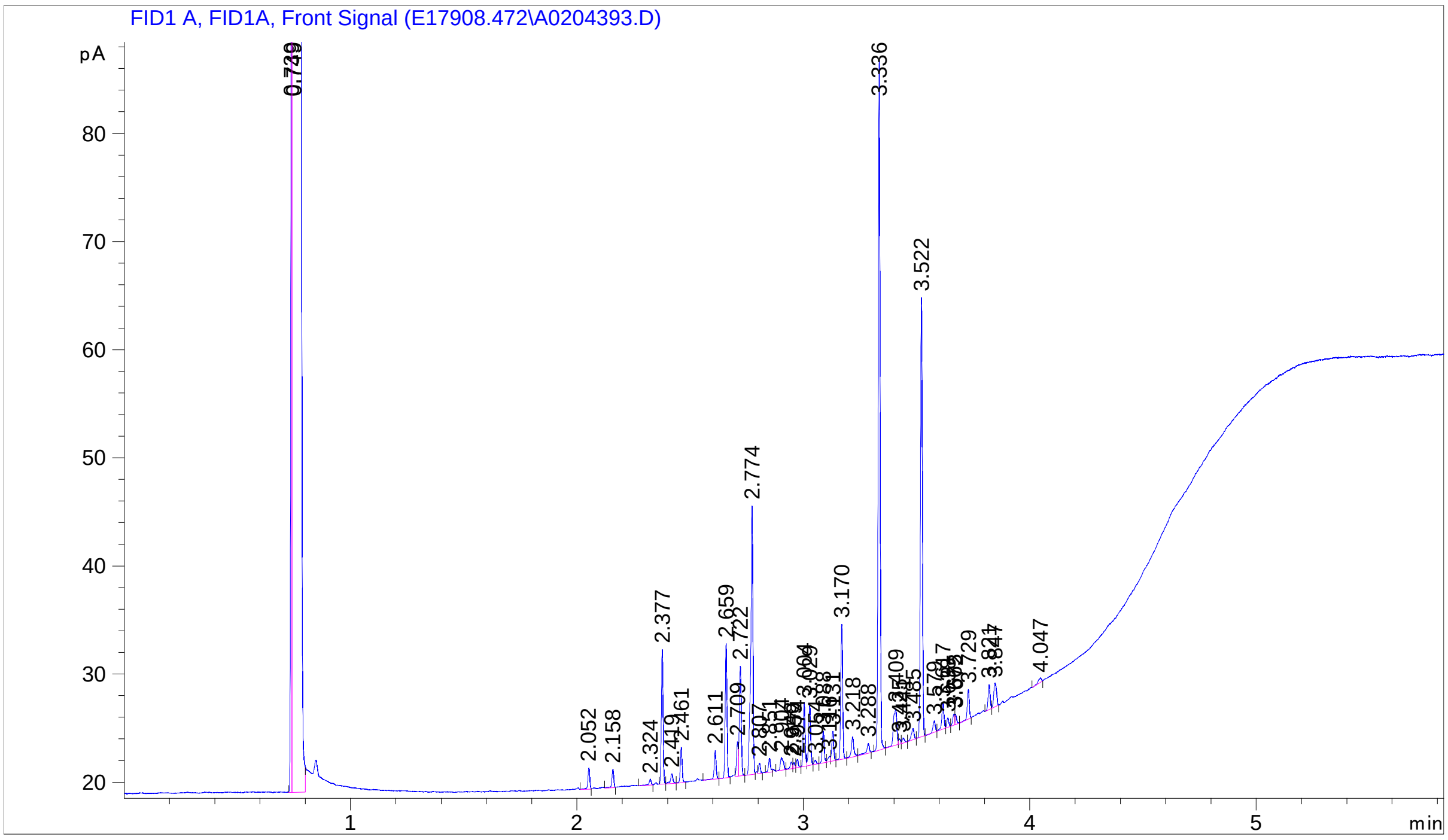

12. FAME_*Arthrobacter phenanthrenivorans* A-66

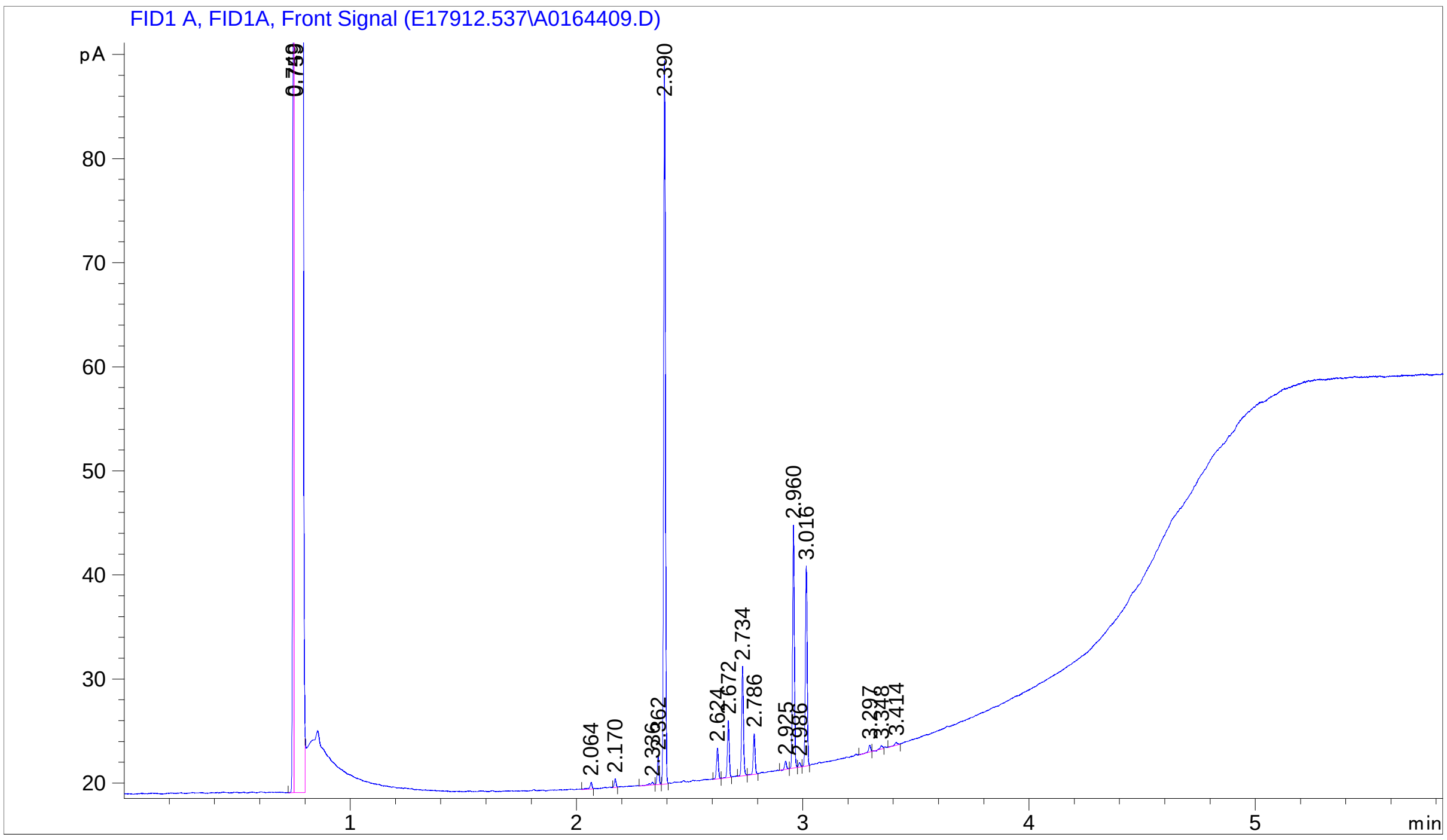

13. FAME_*Brevundimonas vesicularis* A71

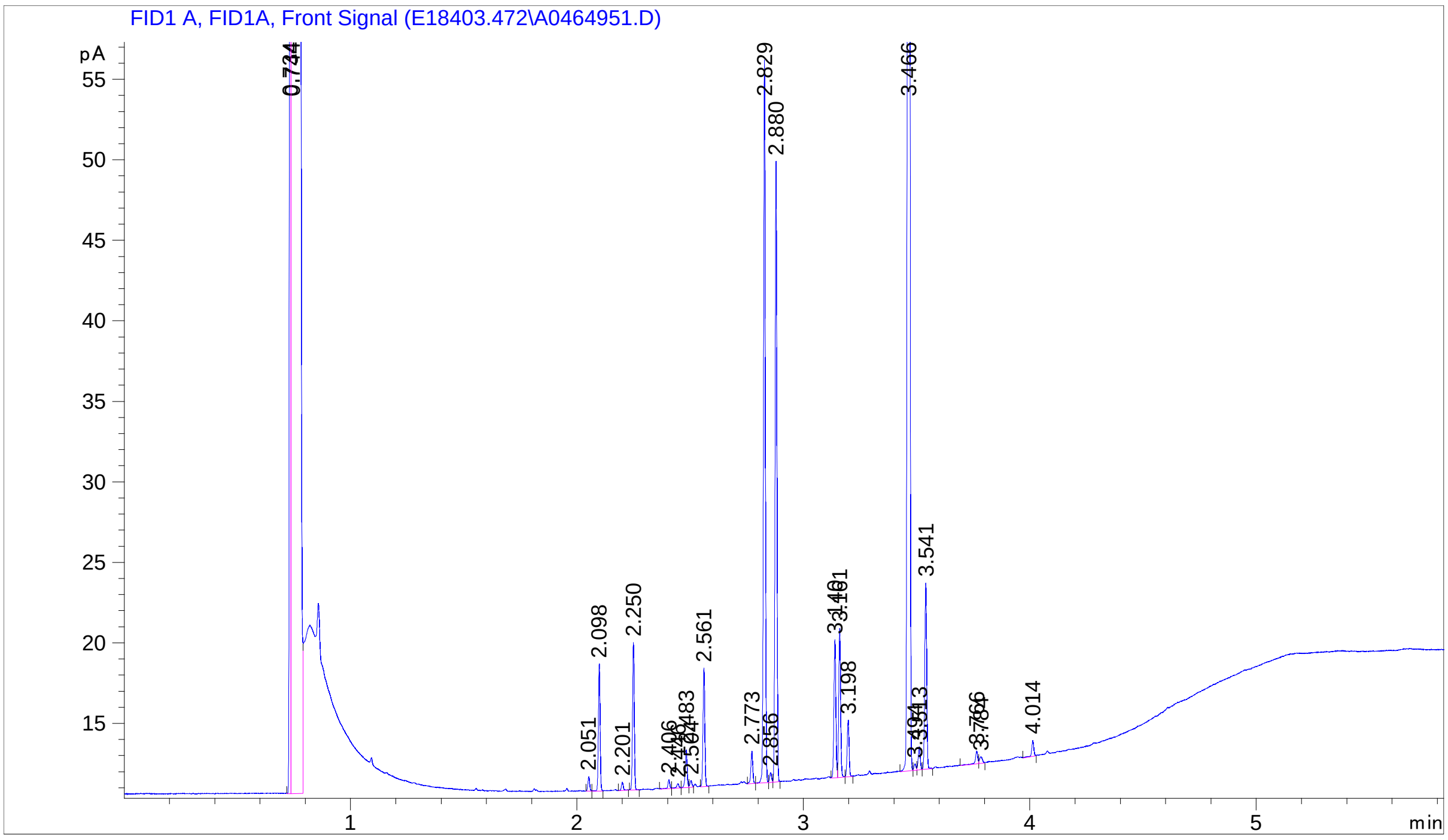

14. FAME_*Microbacterium phyllosphaerae* A135

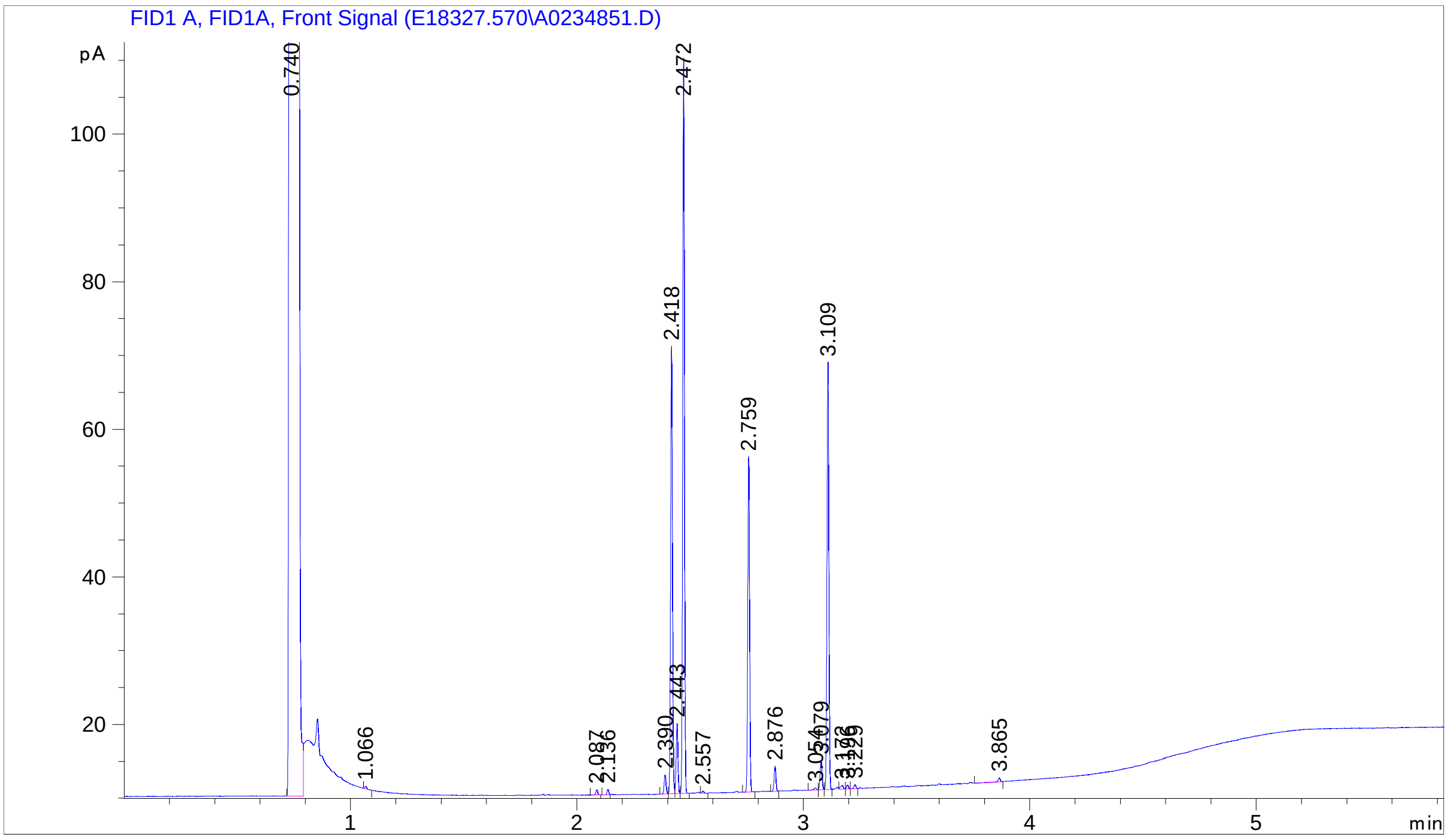

15. FAME_*Moraxella osloensis* A9

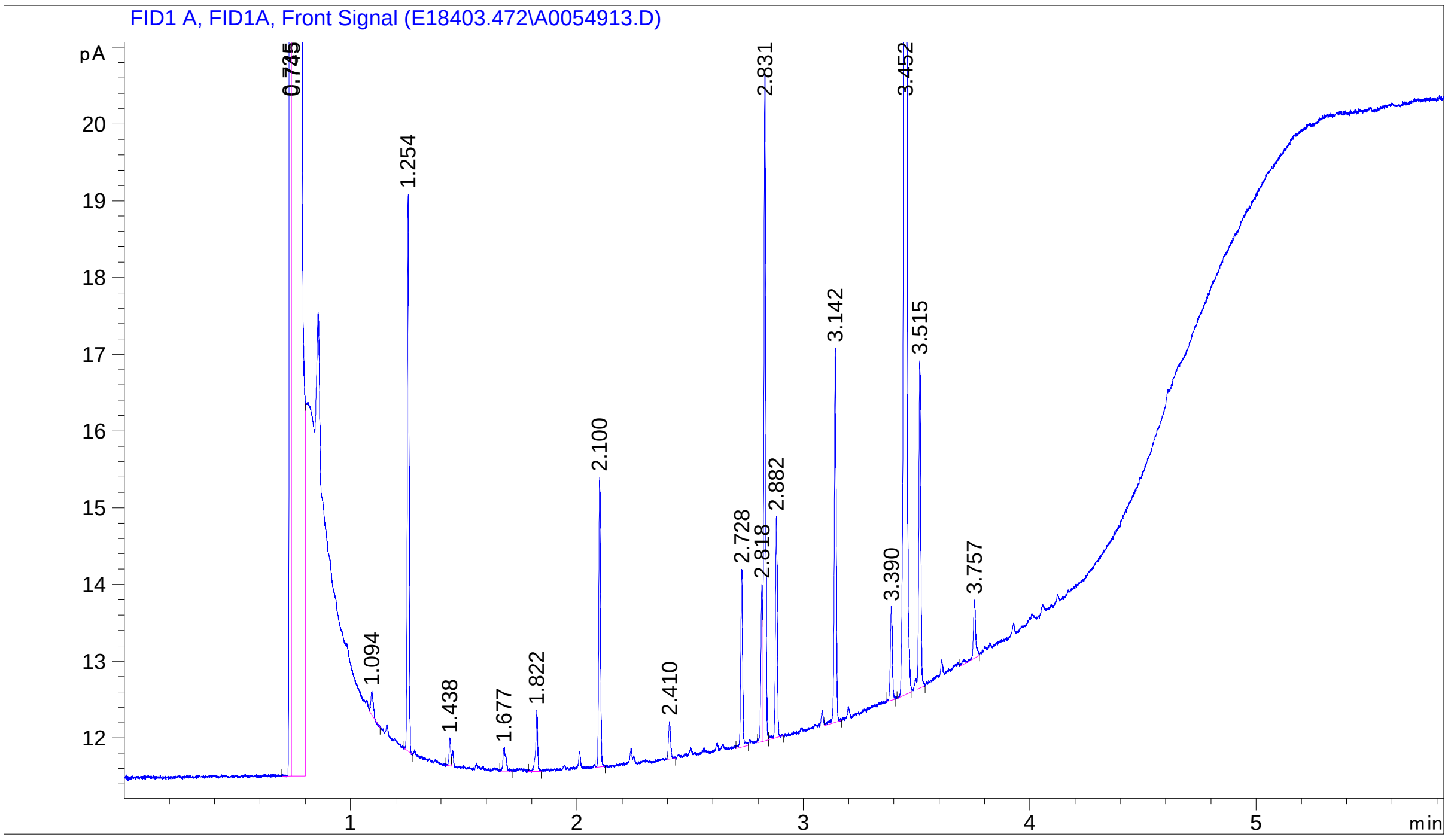

16. FAME_*Pseudomonas hunanensis* A60

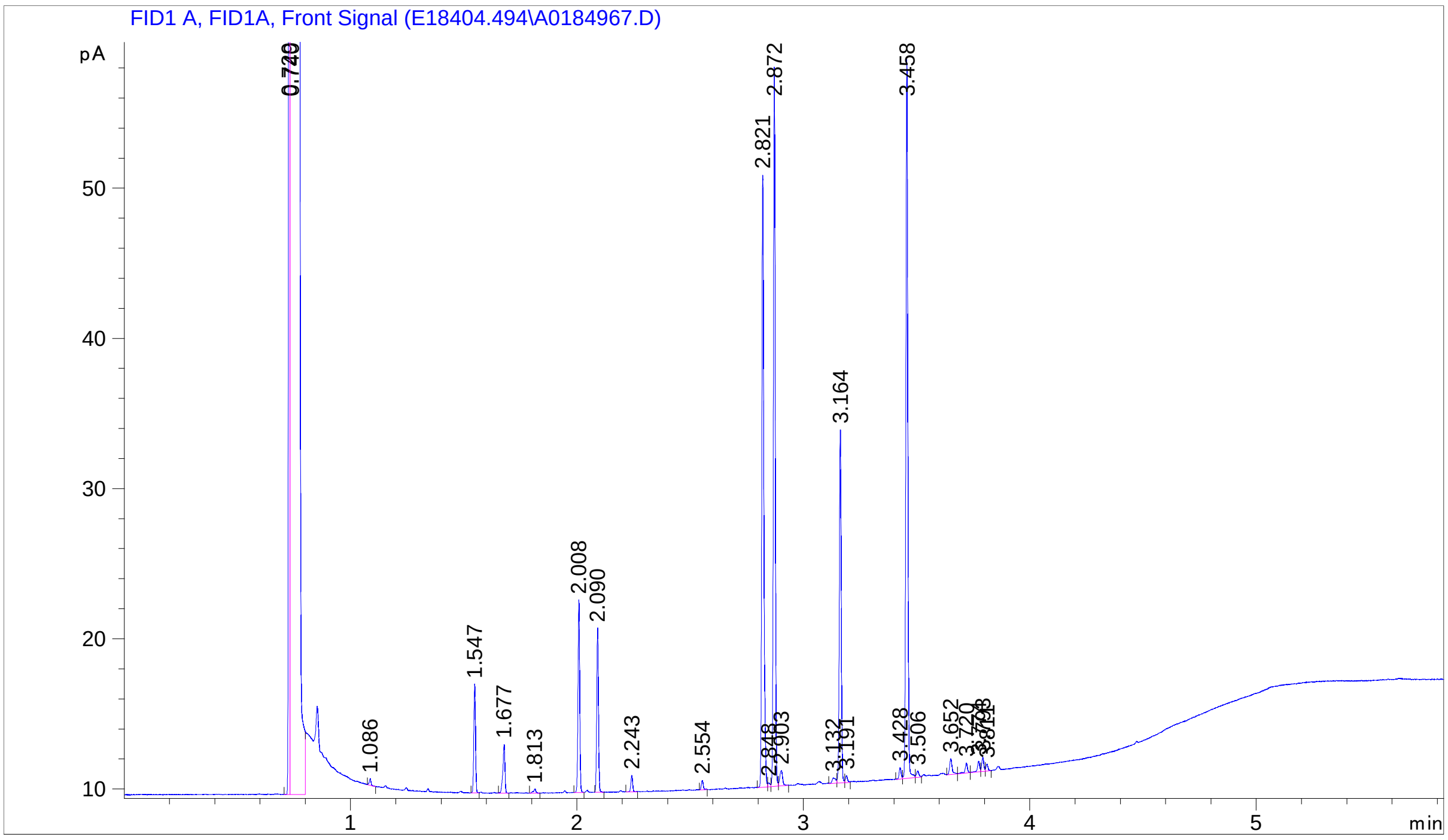

17. FAME_*Rothia* sp A58

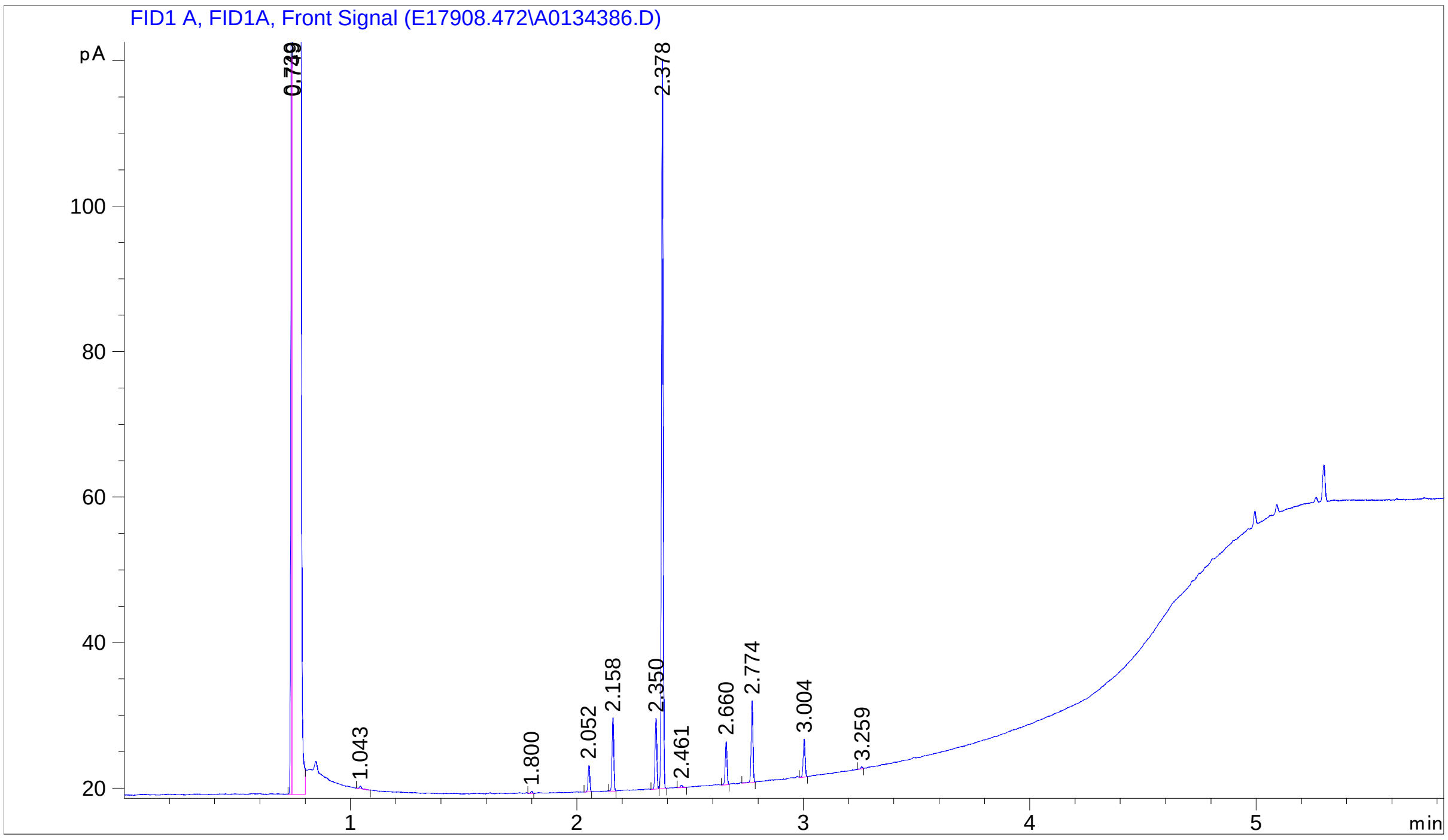

18. FAME_*Knoellia subterranea* A145

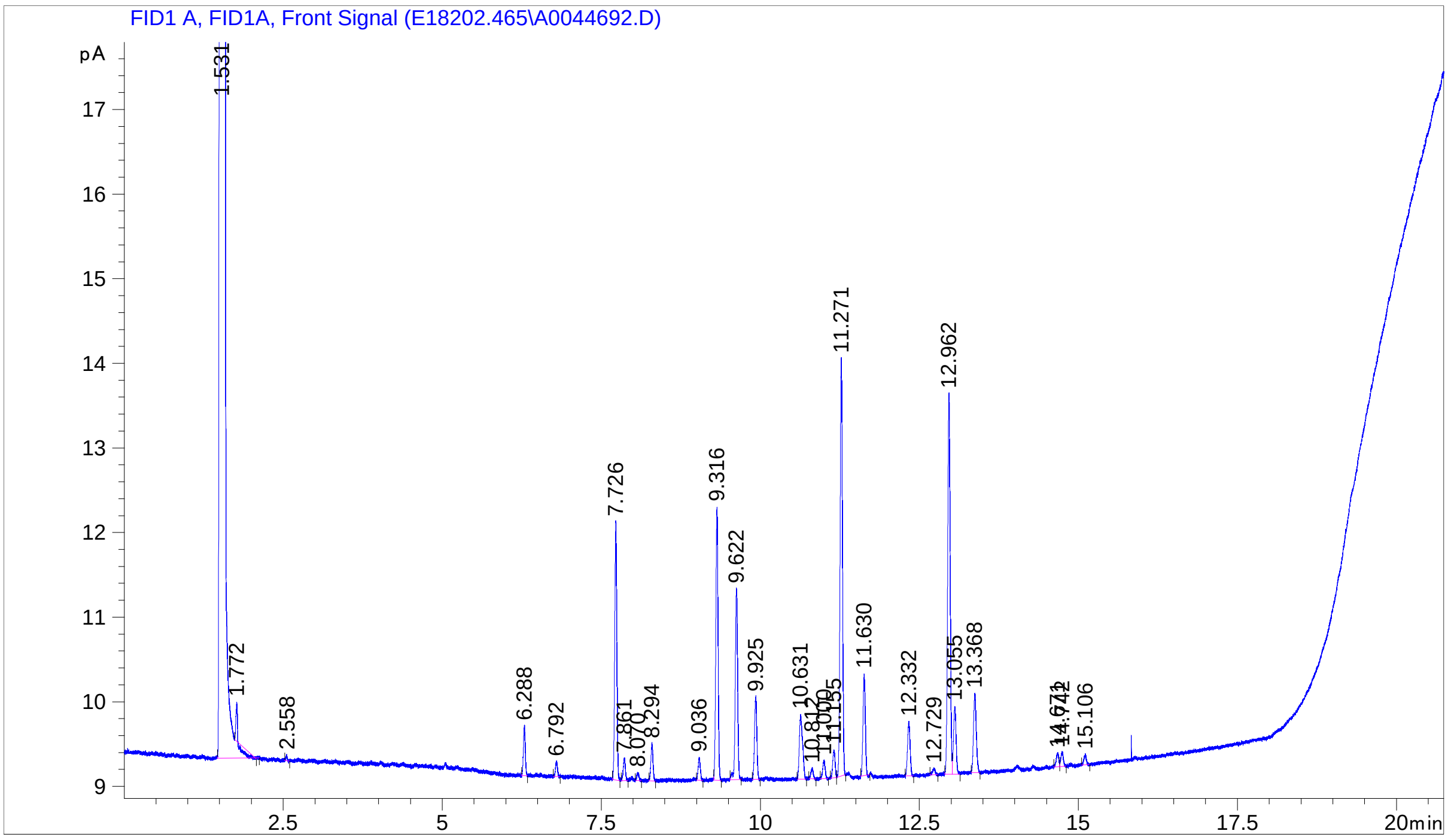

19. FAME_*Paracoccus acridae* A155

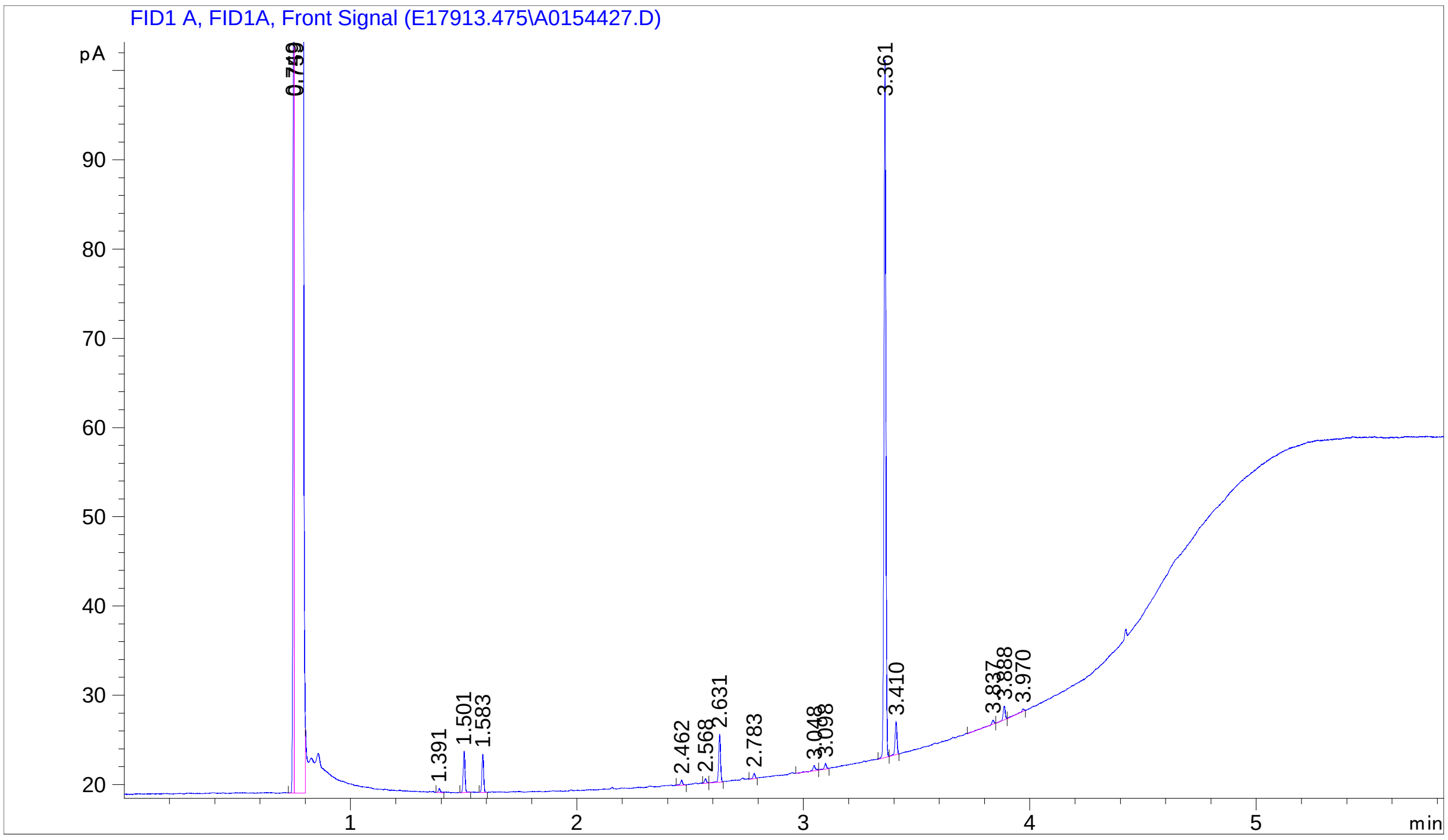

20. FAME_*Methylobacterium rhodesianum* Peng 9

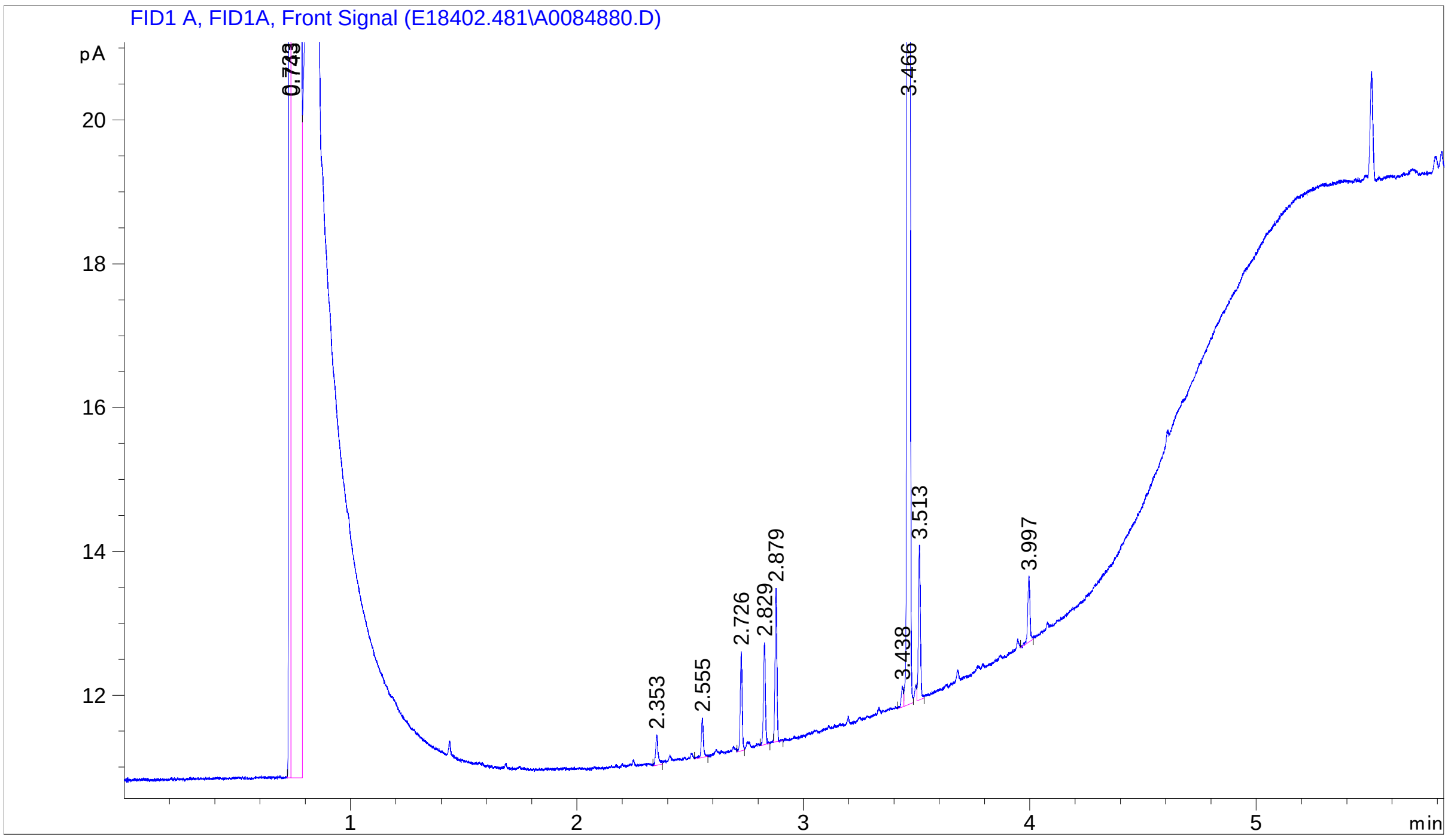

21. FAME_*Acinetobacter baumannii* Peng 6

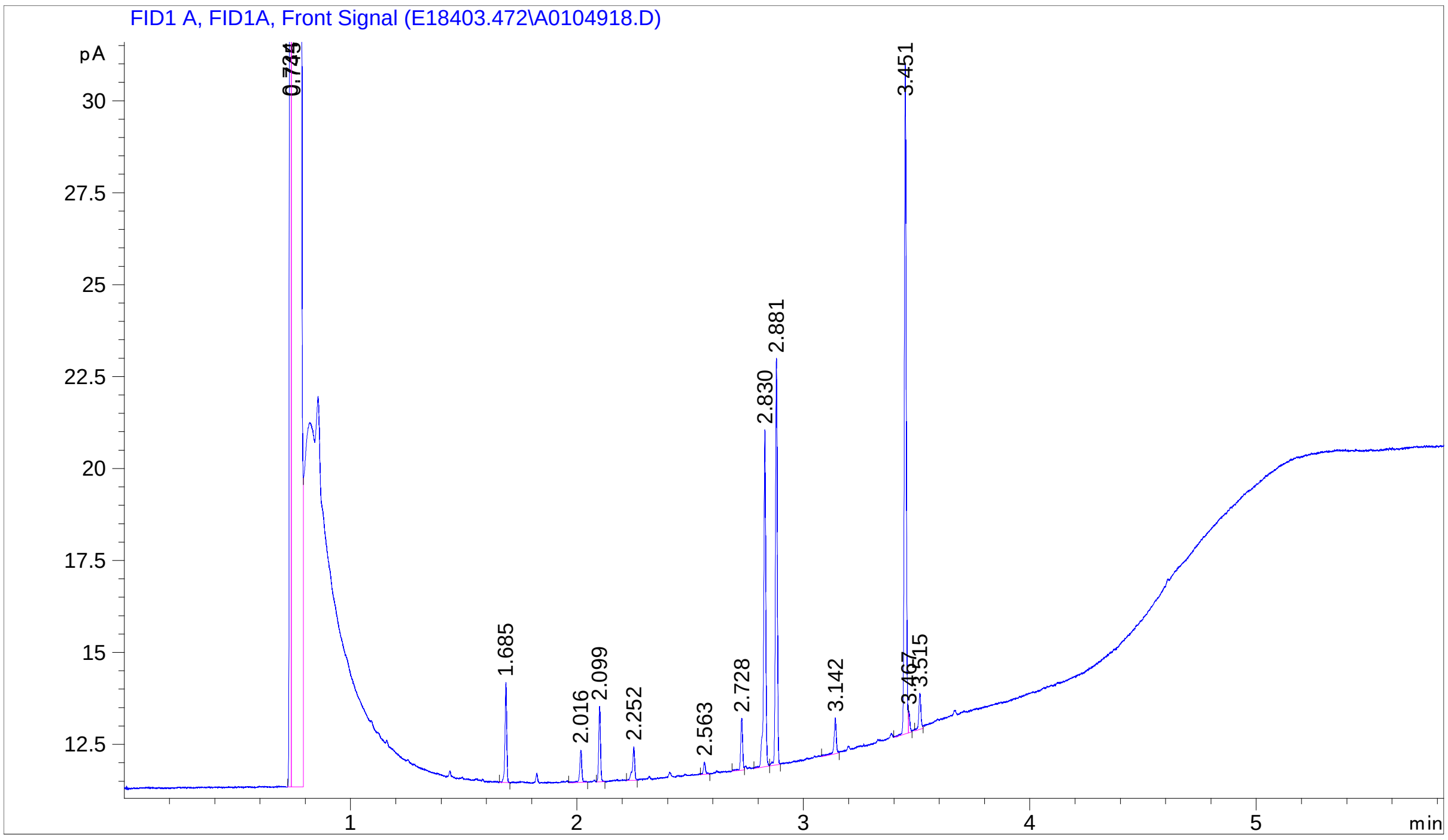

22. FAME_*Brachybacterium rhamnosum* A35

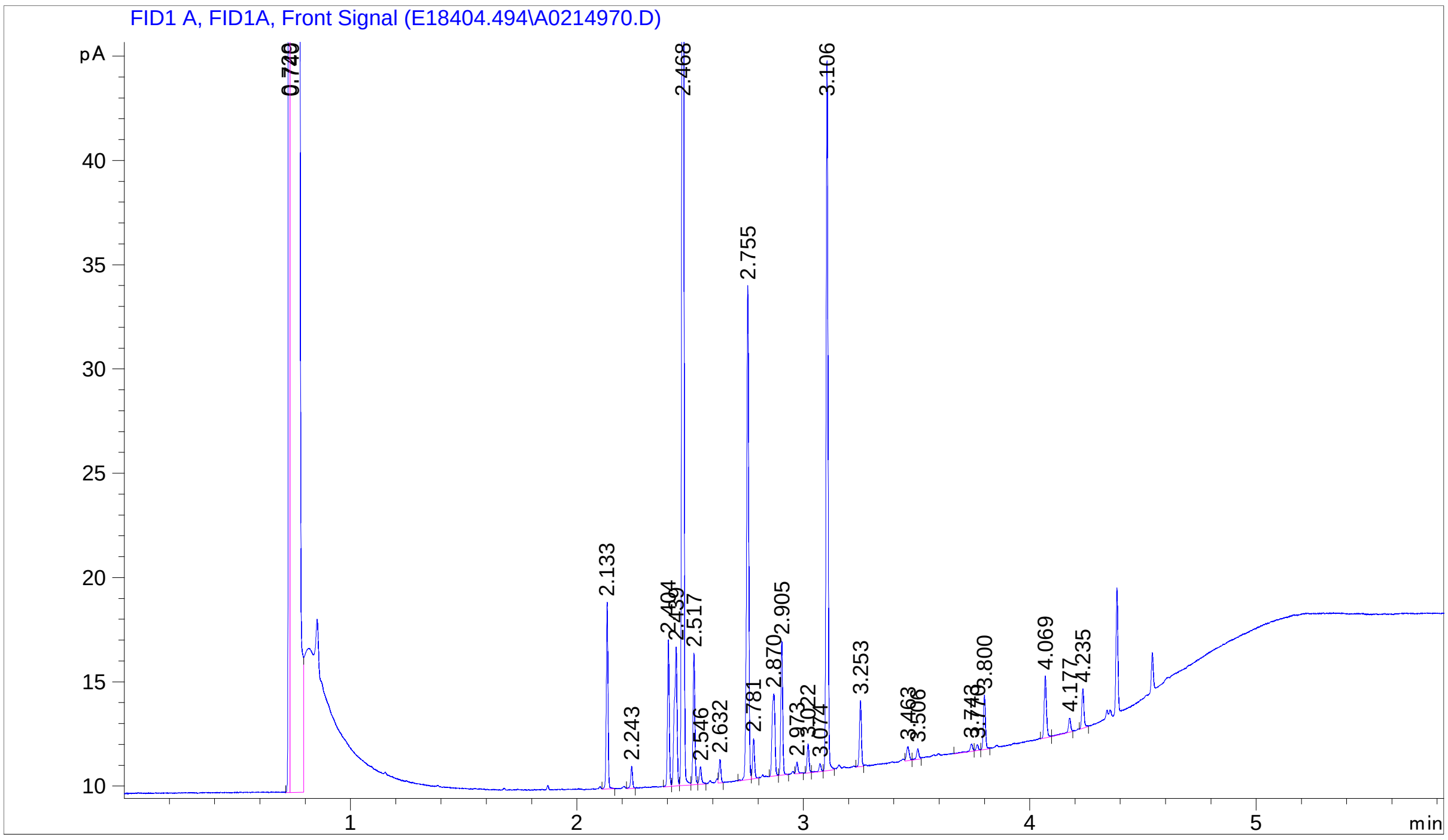

23. FAME_*Arthrobacter polychromogenes* A64

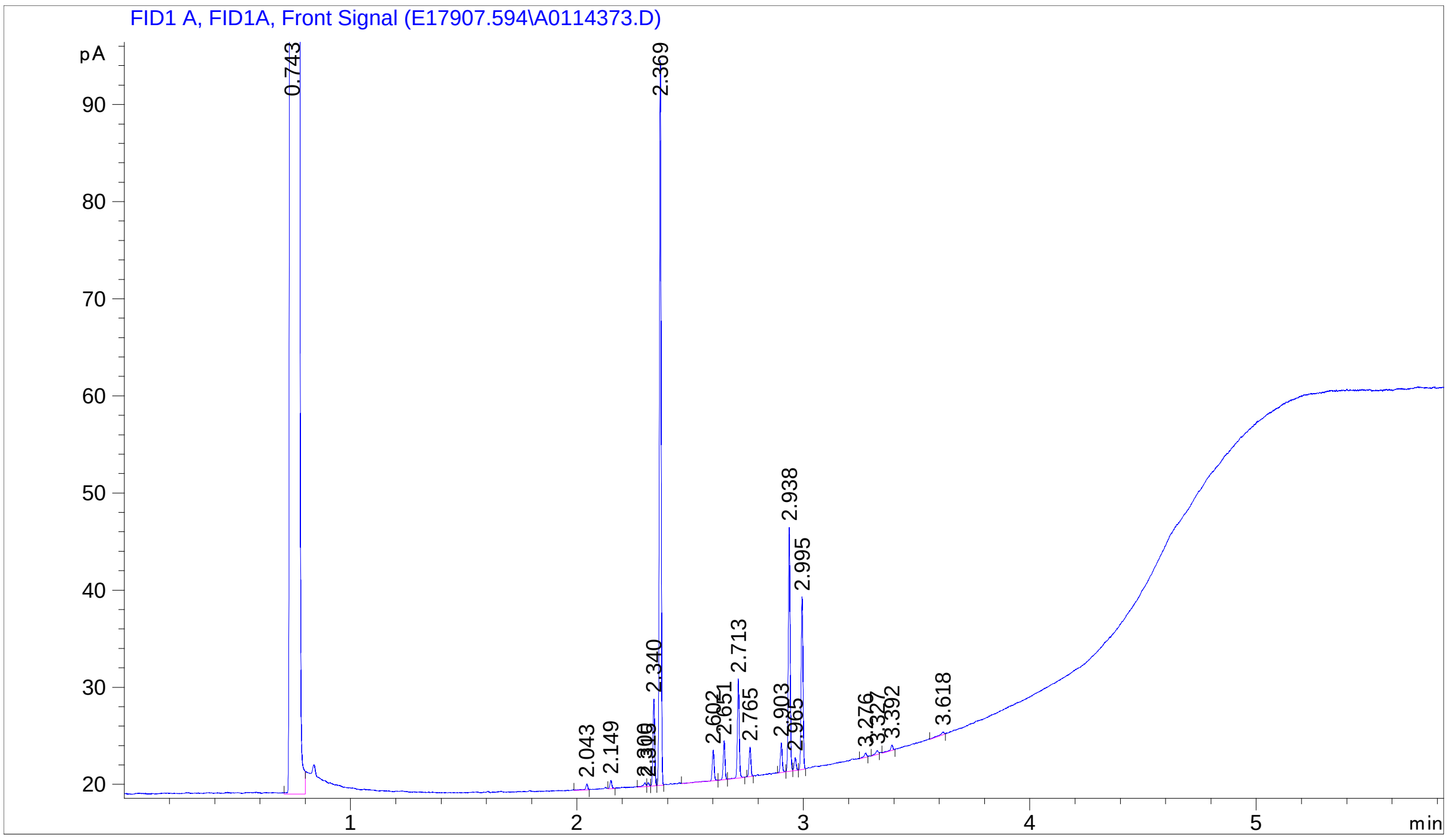

24. FAME_*Lysinibacillus sphaericus* Peng-4

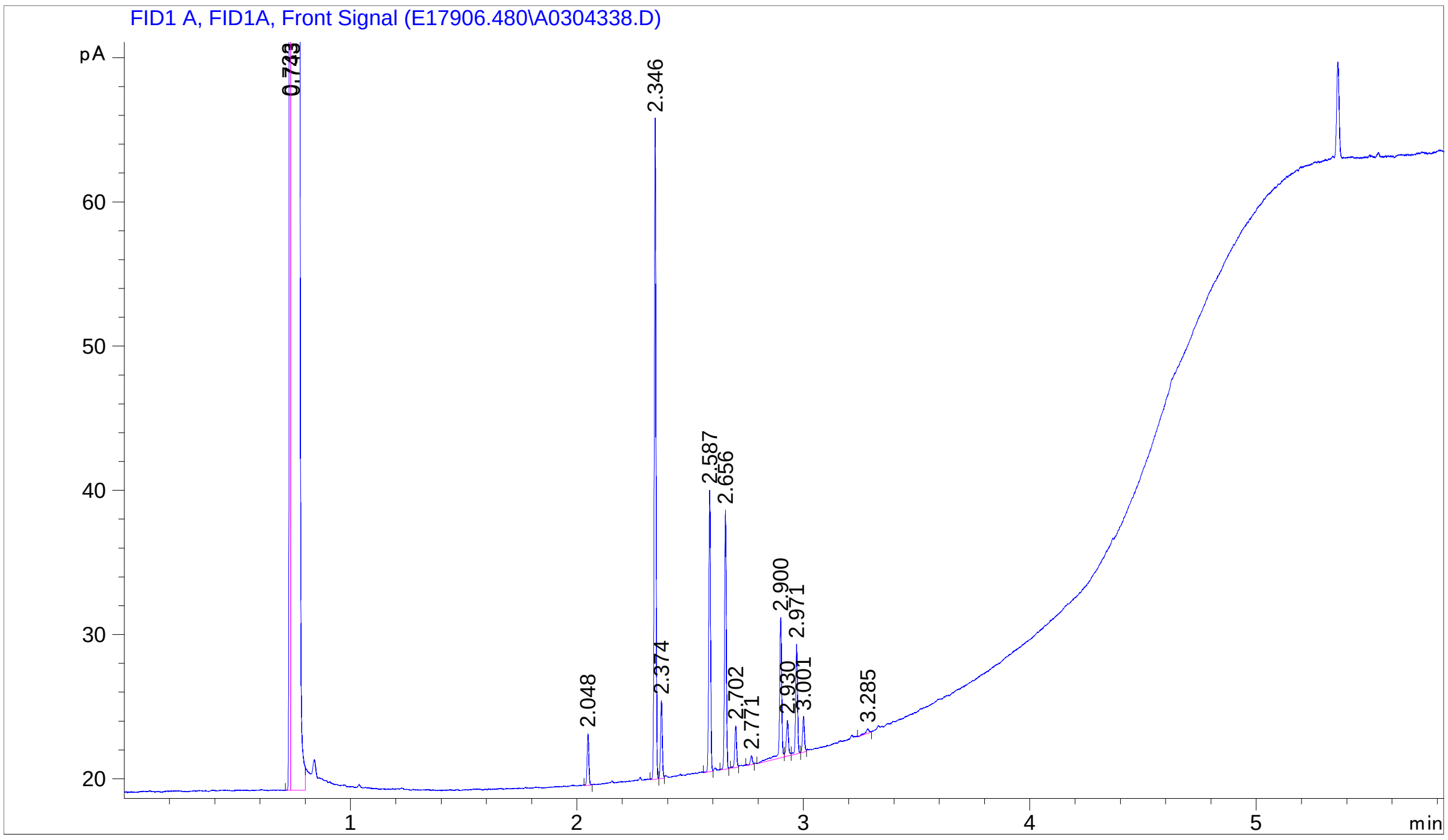

25. FAME_*Micrococcus yunnanensis* A45

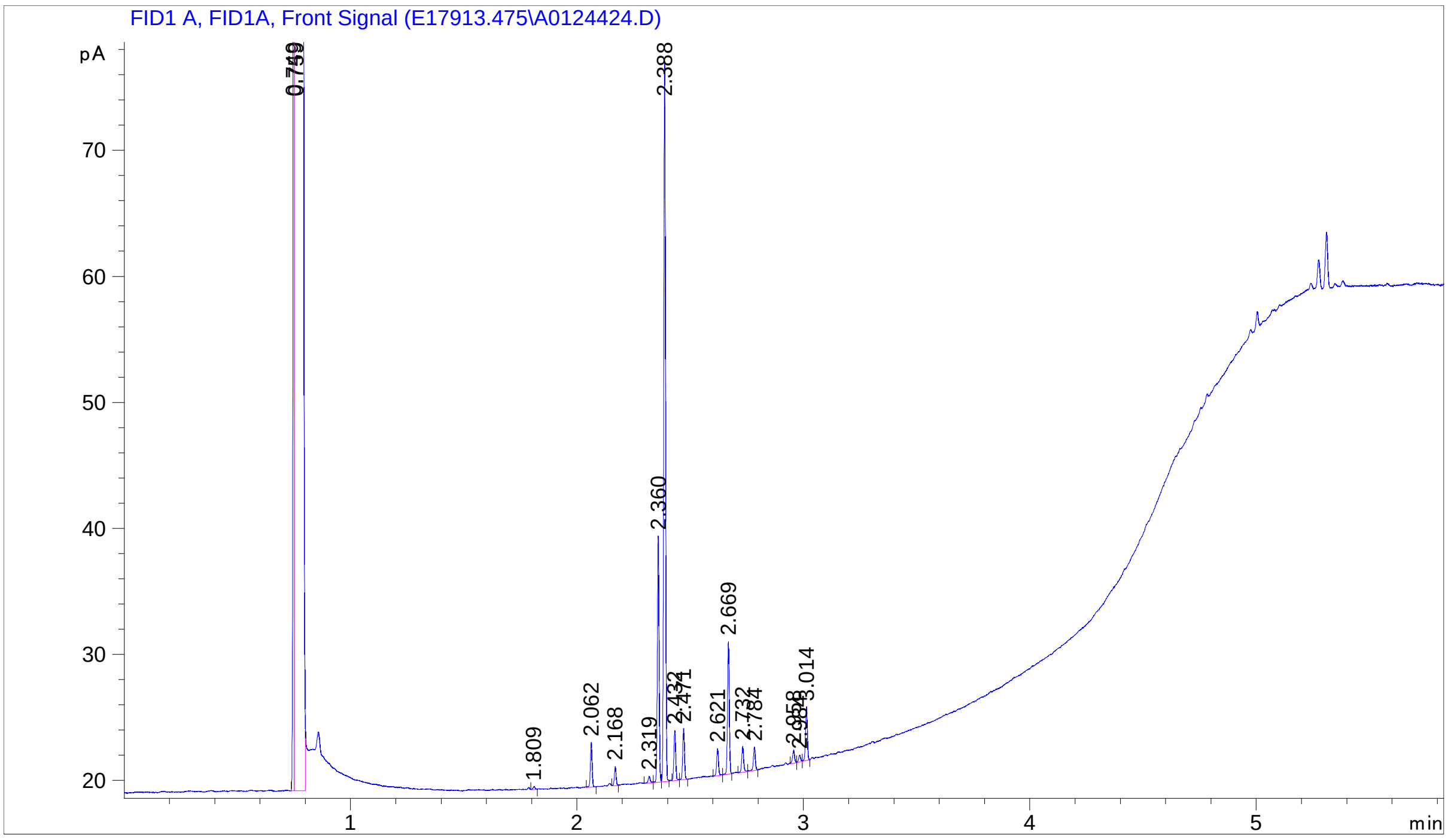

26. FAME_*Rhodococcus sovatensis* A83

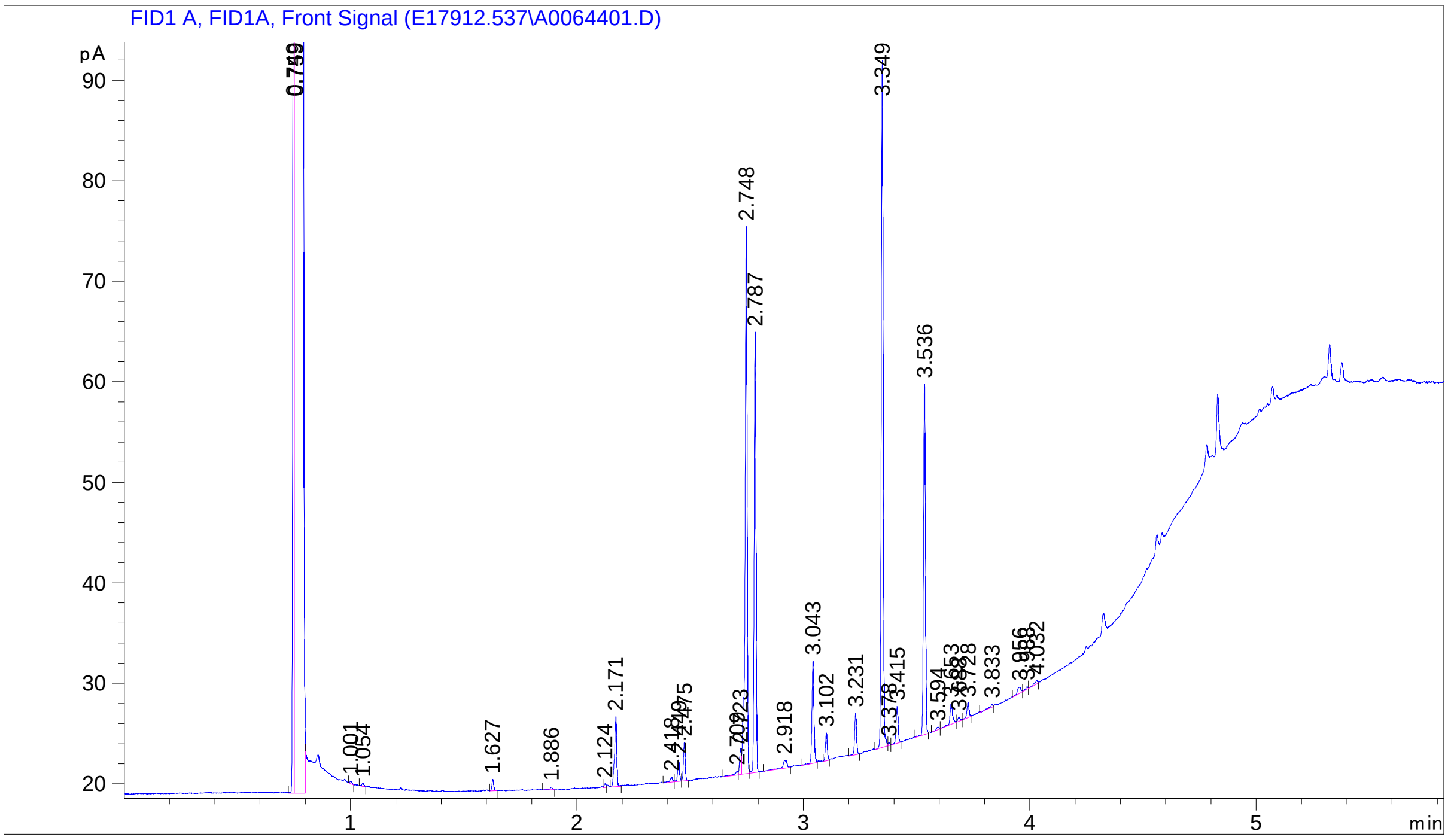

27. FAME_*Staphylococcus gallinarum* A101

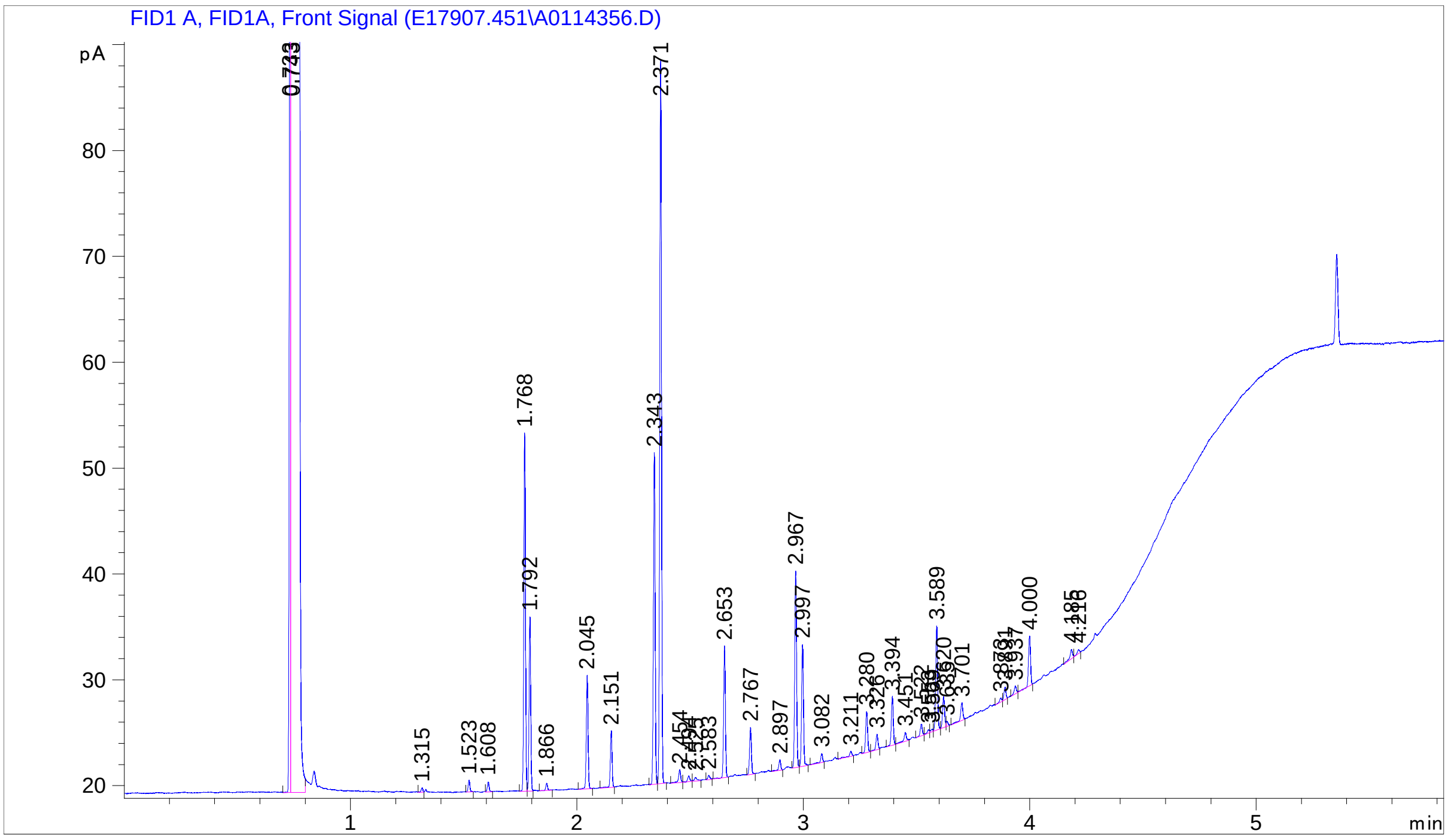

Supplement: Supplementary file 2 [file Data_Sheet_2.PDF]
